# Mass spectrometry‐based top‐down and bottom‐up approaches for proteomic analysis of the Moroccan *Buthus occitanus* scorpion venom

**DOI:** 10.1002/2211-5463.13143

**Published:** 2021-05-28

**Authors:** Khadija Daoudi, Christian Malosse, Ayoub Lafnoune, Bouchra Darkaoui, Salma Chakir, Jean‐Marc Sabatier, Julia Chamot‐Rooke, Rachida Cadi, Naoual Oukkache

**Affiliations:** ^1^ Laboratory of Venoms and Toxins Pasteur Institute of Morocco Casablanca Morocco; ^2^ Laboratory of Molecular Genetics, Physiopathology and Biotechnology Faculty of Sciences Ain Chock Hassan II University of Casablanca Morocco; ^3^ Mass spectrometry for Biology Unit Institut Pasteur CNRS USR 2000 Paris France; ^4^ Laboratory INSERM UMR 1097 University of Aix Marseille France

**Keywords:** bottom‐up, *Buthus occitanus* scorpion, top‐down, toxins, venom, venomic

## Abstract

*Buthus occitanus (B. occitanus)* is one of the most dangerous scorpions in the world. Despite the involvement of *B. occitanus* scorpion in severe cases of envenomation in Morocco, no study has focused yet on the proteomic composition of the Moroccan *B. occitanus* scorpion venom. Mass spectrometry‐based proteomic techniques are commonly used in the study of scorpion venoms. The implementation of top‐down and bottom‐up approaches for proteomic analyses facilitates screening by allowing a global view of the structural aspects of such complex matrices. Here, we provide a partial overview of the venom of *B. occitanus* scorpion, in order to explore the diversity of its toxins and hereafter understand their effects. To this end, a combination of top‐down and bottom‐up approaches was applied using nano‐high liquid chromatography coupled to nano‐electrospray tandem mass spectrometry (nano‐LC‐ESI MS/MS). The LC‐MS results showed that *B. occitanus* venom contains around 200 molecular masses ranging from 1868 to 16 720 Da, the most representative of which are those between 5000 and 8000 Da. Interestingly, combined top‐down and bottom‐up LC‐MS/MS results allowed the identification of several toxins, which were mainly those acting on ion channels, including those targeting sodium (NaScTxs), potassium (KScTxs), chloride (ClScTxs), and calcium channels (CaScTx), as well as antimicrobial peptides (AMPs), amphipathic peptides, myotropic neuropeptides, and hypothetical secreted proteins. This study reveals the molecular diversity of *B. occitanus* scorpion venom and identifies components that may have useful pharmacological activities.

## Graphical abstract

*Buthus occitanus* scorpion possesses highly toxic venom. This article describes the first proteomic screening of the Moroccan *B. occitanus* venom. Our findings highlight the complexity and the toxin diversity of this venom. The most representative compounds are neurotoxins. In addition, antimicrobial peptides, amphipathic peptides, myotropic neuropeptides, and hypothetical secreted proteins were also detected in this venom.

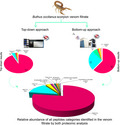

**Abbreviations**
ACN: acetonitrile
AMP: antimicrobial peptides
*B. occitanus*: *Buthus* *occitanus*
CaScTxs: neurotoxins affecting calcium channels
ClScTxs: neurotoxins affecting chloride channels
Da: Dalton
EThcD: Electron‐Transfer/Higher‐Energy Collision Dissociation
FA: formic acid
HCD: higher‐energy C‐trap dissociation
IAA: iodoacetamide
kDa: kilodalton
KScTxs: neurotoxins affecting potassium channels
LC‐MS/MS: liquid chromatography coupled to tandem mass spectrometry
LC‐MS: liquid chromatography coupled to mass spectrometry
MS: mass spectrometry
MW: molecular weight
nano‐LC‐ESI MS/MS: nano‐liquid chromatography coupled to electrospray tandem mass spectrometry
NaScTxs: neurotoxins affecting sodium channels
Q: quadrupole
TIC: total ion chromatogram

Each year, scorpion stings record new cases of envenomation over the world with an incidence of more than 1.5 million and over 2600 deaths, mainly in tropical and subtropical countries of South America, Asia, and North Africa [1]. Most of these envenomation cases were caused by scorpions belonging to the Buthidae family, which contains dangerous species known by their lethal venoms [2]. The venom of these family members contains a heterogeneous cocktail of compounds, including inorganic substances, enzymes, mucopolysaccharides, allergenic compounds, and peptides with high toxicity toward ionic channels of excitable cells [3, 4, 5, 6]. In Morocco, 26 819 cases of scorpion stings were reported in 2019 by the Poison Control and Pharmacovigilance Center of Morocco, with an incidence of 75.3 cases per 100 000 inhabitants [7]. These statistics are due to the diversified scorpion fauna represented by over 50 species, mainly widespread in the middle and southwestern provinces of the kingdom [8]. Among these species, the yellow scorpion *Buthus occitanus (B.occitanus)* seems to be one of the most dangerous scorpions, on account of its toxic venom causing the majority of envenomation cases [9]. Although several studies had been carried out on this venom [10, 11, 12, 13], no study has yet focused on the proteomic composition of the Moroccan *B. occitanus* scorpion venom despite its medical importance. Moreover, there are various strategies to screen scorpion venoms, from using conventional strategies for targeting one single toxin, to applying the most throughput equipment of screening for a detailed view of all toxic components. Nowadays, mass spectrometry‐based proteomic approaches are still one of the most fundamental tools to decrypt the complexity of such matrices, owing to the revolutionary advances in instrumentation and software, in addition to improvement in omics strategies (peptidomic, proteomic, transcriptomic, and genomic) [14, 15, 16, 17, 18, 19]. Among the approaches that have improved significantly the proteomics workflow, there are the top‐down process, which designates a rapid analytical workflow of intact proteins, and the bottom‐up approach, which requires prior proteolytic digestion of proteins before mass spectrometry analysis. These approaches lead to acquiring mass fingerprints, primary structural information, and post‐translational modifications [20, 21, 22, 23]. The application of these approaches, singly or complementary, in several proteomic studies has increased the number of characterized venoms and identified toxins [24, 25, 26, 27, 28, 29]. In this context, this work aimed to ensure an overview of the peptidome of *B. occitanus* scorpion (< 30 kDa), so exploring its toxins arsenal, using a combination of the top‐down and bottom‐up approaches applied on nano‐high liquid chromatography coupled to a nano‐electrospray tandem mass spectrometry (nano‐LC‐ESI MS/MS).

## Materials and methods

### Venom preparation

#### Venom milking

Specimens of *B. occitanus* were collected from the region of Oualidia (32°44′N 9°01′W), in eastern Morocco. The crude venom was milked by electrical stimulation, pooled, centrifuged at 10 000 ***g*** for 20 min, freeze‐dried, and stored at −20 °C until use [30].

#### Venom Reduction/Alkylation

At first, 2 mg of *B. occitanus* crude venom was subjected to a 30 kDa ‘cutoff’ filter (Amicon® Ultra Centrifugal Filters, Merck Millipore, Tulagreen, Ireland), then centrifuged at 16 900 ***g*** for 15 min.

Disulfide‐bridged half‐cysteine residues of this venom filtrate were reduced by 10 mm of DTT in ammonium bicarbonate buffer (50 mm, pH 8.3), for 45 min at a temperature of 56 °C. Cysteine residues were carboxamido‐methylated by incubation with 50 mm iodoacetamide [IAA in ammonium bicarbonate (50 mm, pH 8.3)] for 1 h in the dark. Then, these proteins/peptides were desalted by ZipTip C4 (Millipore Corporation ‐ Billerica, USA) and concentrated on a Savant SpeedVac (Thermo Scientific, San Jose, CA, USA).

### Mass spectrometry‐based proteomic approaches

#### Top‐down proteomics

**Fig. 1:**
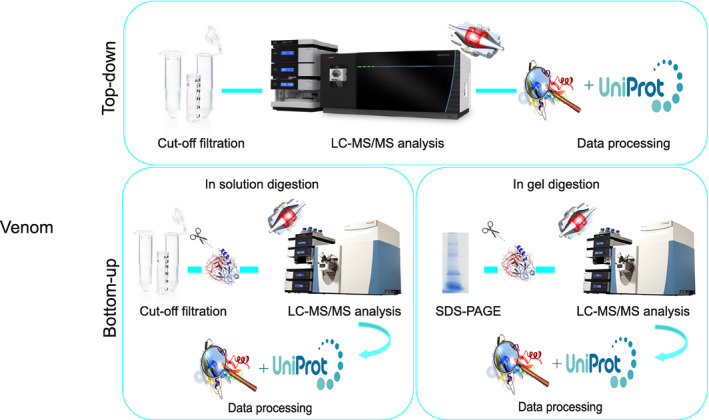
Experimental workflow performed in this study. At first, *B. occitanus* venom was milked by electrical stimulation and applied to a 30 kDa filter. For the top‐down venomic, the flow‐through containing toxins < 30 kDa was analyzed by the Thermo Scientific ™ Orbitrap Fusion Lumos Tribrid Mass Spectrometer. For the bottom‐up approach, two digest methods were achieved: 1) in‐solution digestion, the flow‐through containing toxin < 30 kDa was directly reduced with DTT, alkylated with IAA, and digested with trypsin; and 2) in‐gel digestion, the unstained gel was excised to small cubes, reduced, alkylated, and digested. The digest peptides were then desalted with ZipTip and applied to the Orbitrap Q‐Exactive mass spectrometer.

Intact and reduced/alkylated *B. occitanus* venom filtrates were carried out on an Orbitrap Fusion™ Lumos™ mass spectrometer (Thermo Scientific™ Waltham, MA, USA), equipped with a Dionex HPLC (Fig. 1).

For the online peptide fractionation, 2 µg of samples was loaded to a C4 µ‐precolumn cartridge (300 µm i.d. × 5 mm, C4 PepMap 300 particles with 5 µm size and 300 Å pores); the column was equilibrated with solution A [0.1% (v/v) formic acid (FA)]. The separation was maintained over 120 min at 250 nL·min^−1^, using a linear gradient from 5% to 60% of solution B [acetonitrile (ACN) and 0.1% (v/v) FA].

Proteins/peptides were eluted directly from the column into the mass spectrometer and operated in positive mode with a spray voltage of 1.6 kV. MS spectra were acquired at a resolution setting of 120 000.

MS/MS analysis was performed on data‐dependent acquisition, the top 10 abundant precursor ions were selected for an EThcD fragmentations (Electron‐Transfer/Higher‐Energy Collision Dissociation) with a dynamic exclusion time of 90 s. MS/MS spectra were acquired at a resolution setting of 120 000, and the mass range was set from 150 to 2000 *m/z*.

#### Bottom‐up proteomics

##### In‐solution digestion

Reduced/alkylated venom filtrate was digested overnight at a temperature of 37 °C with 0.1 μg of trypsin (Promega, Madison, WI, USA). Tryptic digests were analyzed on a Q‐Exactive Plus instrument (Thermo Fisher Scientific, Bremen, Germany) coupled to an EASY‐nLC 1200 chromatography system (Thermo Fisher Scientific). Two micrograms was loaded on an in‐house packed 50‐cm nano‐HPLC column (75 μm inner diameter) filled with C18 resin (1.9 μm particles, 100 Å pore size, Reprosil‐Pur Basic C18‐HD resin; Maisch GmbH, Ammerbuch‐Entringen, Germany) and equilibrated in 97% solvent A and 3% solvent B (ACN, 0.1% (v/v) FA).

Peptides were eluted at 250 nL·min^−1^, using 3–22% gradient of solvent B for 112 min, then 22–38% gradient of solvent B for 35 min, and finally 38–60% gradient of solvent B for 15 min. The instrument method for the Q‐Exactive Plus was set up in the data‐dependent acquisition mode. MS and MS‐MS spectra were acquired at a resolution of 60 000, 10 of the most abundant precursor ions were selected for HCD fragmentation with collision energy adjusted to 27. Mono‐charged precursors and those with a charge state of > 7 were excluded.

##### In‐gel digestion

At first, 2 mg of venom filtrate was unfolded for 5 min at 95 °C in sample buffer (LDS sample buffer) and then subjected to a SDS/PAGE using a 4–20% of polyacrylamide gel (SDS Precast Gel RunBlue, 4–20%, 12 well; Expedeon, CA, USA). The electrophoresis was performed, on a Bio‐Rad system, at a constant voltage of 140 V, and the separated proteins were stained with Coomassie Brilliant Blue R (InstantBlue; Expedeon, CA, USA).

Stained bands corresponding to proteins/peptides with masses < 30 kDa (Fig. S1) were manually excised into equal small cubes of 1 mm^3^, then washed with Milli‐Q water, ammonium bicarbonate 50 mm, and ACN 50%. Subsequently, the slices were submitted to an in‐gel reduction with DTT (10 mm) in ammonium bicarbonate buffer (50 mm, pH 8.3) for 45 min at a temperature of 56 °C. Reduced slices were alkylated with IAA (50 mm) in ammonium bicarbonate (50 mm, pH 8.3) buffer for 20 min in the dark, followed by an overnight digestion with 0.1 μg of trypsin (Promega) at a temperature of 37 °C [31]. The enzymatic reaction was stopped by adding 5 µL of FA 5%, and desalted by loading the peptides onto ZipTip C18. After drying, digested peptides were dissolved in 100 μL of 0.1% (v/v) FA and applied on a liquid chromatography coupled to tandem mass spectrometry (LC‐MS/MS) system, composed of a nano‐flow HPLC pump and an Orbitrap Q‐Exactive mass spectrometer (Thermo Scientific) with a nano‐electrospray ion source, as described in the section above.

#### Data analysis

The top‐down liquid chromatography coupled to mass spectrometry (LC‐MS) data analysis of native *B. occitanus* venom filtrate was deconvoluted using the Xtract algorithm within Thermo Scientific xcalibur 2.2 software (Thermo Fisher Scientific).

For protein identification, data from both of the venomic nano‐LC‐MS/MS approaches were processed using the proteome discover 2.2 software (Thermo Fisher Scientific), against the UniProtKB database, downloaded in 2016 10 11, taxon identifier: 6855 and 4309 entries.

Parameters of processing were as follows: a mass tolerance of MS set at 50 p.p.m. and 0.3 Da for MS/MS. One unique peptide was required for protein identification, minimum peptide length was required at five amino acids, and the false discovery rate cutoff was 1%. Trypsin was chosen as the specific enzyme, with a maximum number of two missed cleavages for the bottom‐up analysis. Variable modifications included oxidation of methionine and carbamidomethylation, while no fixed modification was set.

## Results

### Mass spectrometry‐based proteomic approaches

The whole proteomic approaches are based only on the UniProtKB database‐dependent analysis without any manually *de novo* sequence annotation; therefore, the majority of reported peptide annotations are still an approximation. Also, it is important to stress that the relative abundances and the percentages of the described peptides are purely based on total number counts and not concentrations as long as no quantitative analysis was performed.

#### Top‐down proteomics

**Fig. 2:**
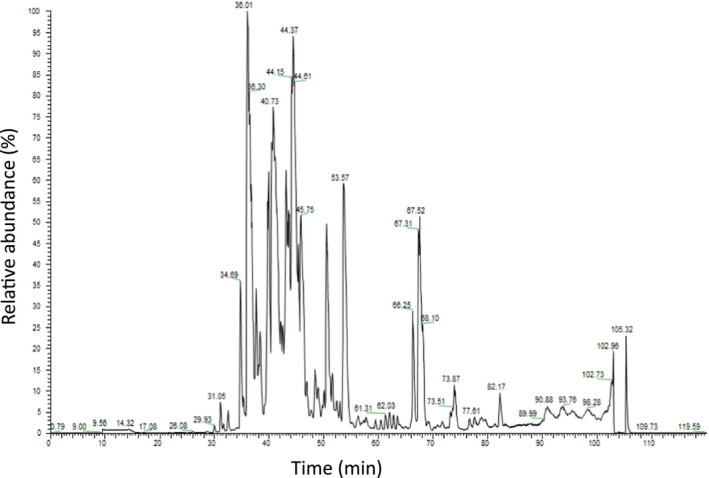
TIC of native *B. occitanus* venom filtrate, generated from top‐down mass spectrometry analysis (MS1). The *x*‐axis represents the relative abundance (%), and the *y*‐axis, the retention time (min). Spectra were deconvoluted, and generated monoisotopic masses were distributed according to their MW.

The total ion chromatogram (TIC) generated from the top‐down LC‐MS analysis of native *B. occitanus* venom filtrate (Fig. 2) gave a partial picture of the venom complexity, with around 60 peaks, most of them detected with high relative abundance.

The mass fingerprint of *B. occitanus* venom was generated from a manual deconvolution of spectra gained by top‐down LC‐MS approach, thus detecting a total of 197 monoisotopic masses ranging from 1868 to 16 720 Da (Table 1). We get one mass less than 2000 Da, 28 molecular masses ranging between 2000 and 5000 Da, 147 mass values from 5000 to 8000 Da, and 21 masses for those over 8000 Da.

**Table 1:** List of the 197 monoisotopic masses detected by the top‐down LC‐MS analysis.

Retention time (min)	MW (Da)
0–10	N.D
10–20	1868.0157
20–30	2208.2634; 2506.4634
30–40	2813.4212; 2851.4287; 2966.3848; 3124.4545; 3219.5691; 3233.4756; 3461.4966; 3486.7774;3538.283; 3550.4334; 3670.8935; 3718.7023; 3823.4412; 3937.8078; 4093.8732; 4321.8654; 4366.9752; 4366.986; 5731.6152; 5919.5155.
40–50	3522.2898;3614.8741; 3807.4466; 3937.7725; 4333.933; 4366.9856; 4568.7172; 4572.9253; 5185.3781; 6148.8879; 6423.7104; 6439.6786; 6527.7246; 6539.6502; 6541.7326; 6595.7719; 6606.8166; 6610.768; 6611.7946; 6635.0442; 6744.712; 6829.8098; 6831.8926; 6832.876; 6860.9183; 6861.9012; 6872.9404; 6876.9037; 6877.9284; 6893.9821; 6940.948; 6952.1809; 6974.2357; 6979.0052; 6995.0399; 6997.024; 7014.2508; 7016.0204; 7022.0148; 7024.0653; 7107.2902; 7152.0763; 7162.3796; 7177.1647; 7218.3026; 7220.0387; 7220.2052; 7243.2414; 7297.2395; 7393.2604.
50–60	6488.9021; 6609.8127; 6611.7977; 6629.8447; 6677.8651; 6749.8876; 6765.9533; 6779.2433;6807.922; 6823.1194; 6836.974; 6837.8837; 6862.9698; 6879.9966; 6907.3347; 6919.9628; 6972.7789; 7007.0404; 7011.1444; 7012.1231; 7020.055; 7028.0976; 7035.2491; 7024.1049; 7051.0799; 7061.1245; 7062.1114; 7069.1111; 7079.1299; 7082.3444; 7115.0302; 7115.2113; 7122.274; 7130.9674; 7143.0368; 7250.1077; 7262.1172; 7266.1721; 7268.152; 7283.1496; 7307.2070; 7328.1353; 7394.3224; 7394.5252; 7400.289; 7416.5358; 7435.2763; 7449.3831; 7468.4297; 7491.1348; 7506.1972; 7534.4067; 7607.5077; 7681.4621; 7777.5363; 7840.6401; 7894.5677; 7912.5297; 7924.5736; 7943.5256; 8174.6428; 8344.5958; 9875.9204; 6896.9694; 6880.9937; 7016.998; 7056.1905; 7074.1478; 7104.0354; 7122.2913; 7115.9848; 7175.0715; 7309.2612; 7414.4224; 7600.5; 7654.5083; 7798.6334; 7817.6424; 7832.6366; 7833.6635; 8140.6441; 8159.4822; 8345.5484; 9959.0054; 11068.3376; 11243.5823;16720.7335.
60–70	6896.9694; 6880.9937; 7016.998; 7056.1905; 7074.1478; 7104.0354; 7122.2913; 7115.9848; 7175.0715; 7309.2612; 7414.4224; 7600.5; 7654.5083; 7798.6334; 7817.6424; 7832.6366; 7833.6635; 8140.6441; 8159.4822; 8345.5484; 9959.0054; 11068.3376; 16720.7335.
70–80	6809.9307; 6857.9428; 6859.9368; 6865.9432; 6875.9565; 6880.9796; 6982.0159; 6913.9378; 7009.0523; 7104.9914; 7172.1987; 7200.1528; 7214.1558; 7316.2804; 7377.2599; 7300.0933; 7394.5084.
80–90	7377.2678; 7301.1747; 9140.1069; 11377.1636; 12971.6074; 13004.7435.
90–100	7390.4025; 7466.4483; 7482.4543; 7500.4753; 7704.4655; 7791.5128; 7792.5813; 8672.6993; 8882.0067; 8978.0645; 14577.4253.
100–110	9302.1043; 12990.2825; 12985.6009.
110–120	N.D

N.D: not determined.

**Fig. 3:**
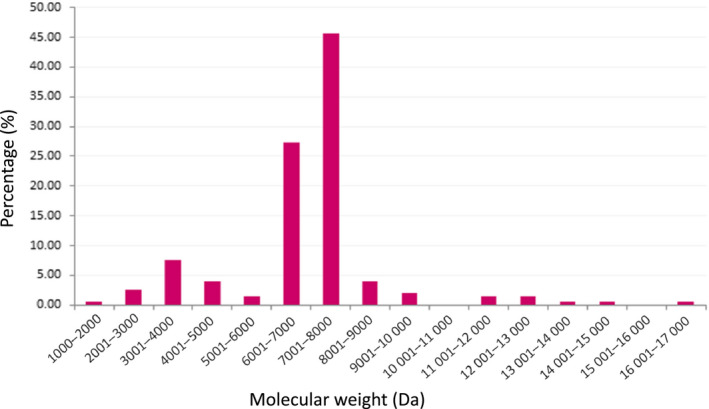
Molecular mass distribution of the monoisotopic masses from MS1 spectra deconvolution. 197 components were detected, with their MW ranging from 1868 to 16 720 Da. These peptides distributed from 1000 to 17 000 Da with 1000 Da mass range windows. The *x*‐axis represents the MW in Da, and the *y*‐axis represents the percentage (%) based on total number counts.

The most representative molecular masses were those from 5000 to 8000 Da, followed by those between 2000 and 5000 Da, which represents respectively 74% and 10% of the total number of measured molecular masses (Fig. 3).

**Fig. 4:**
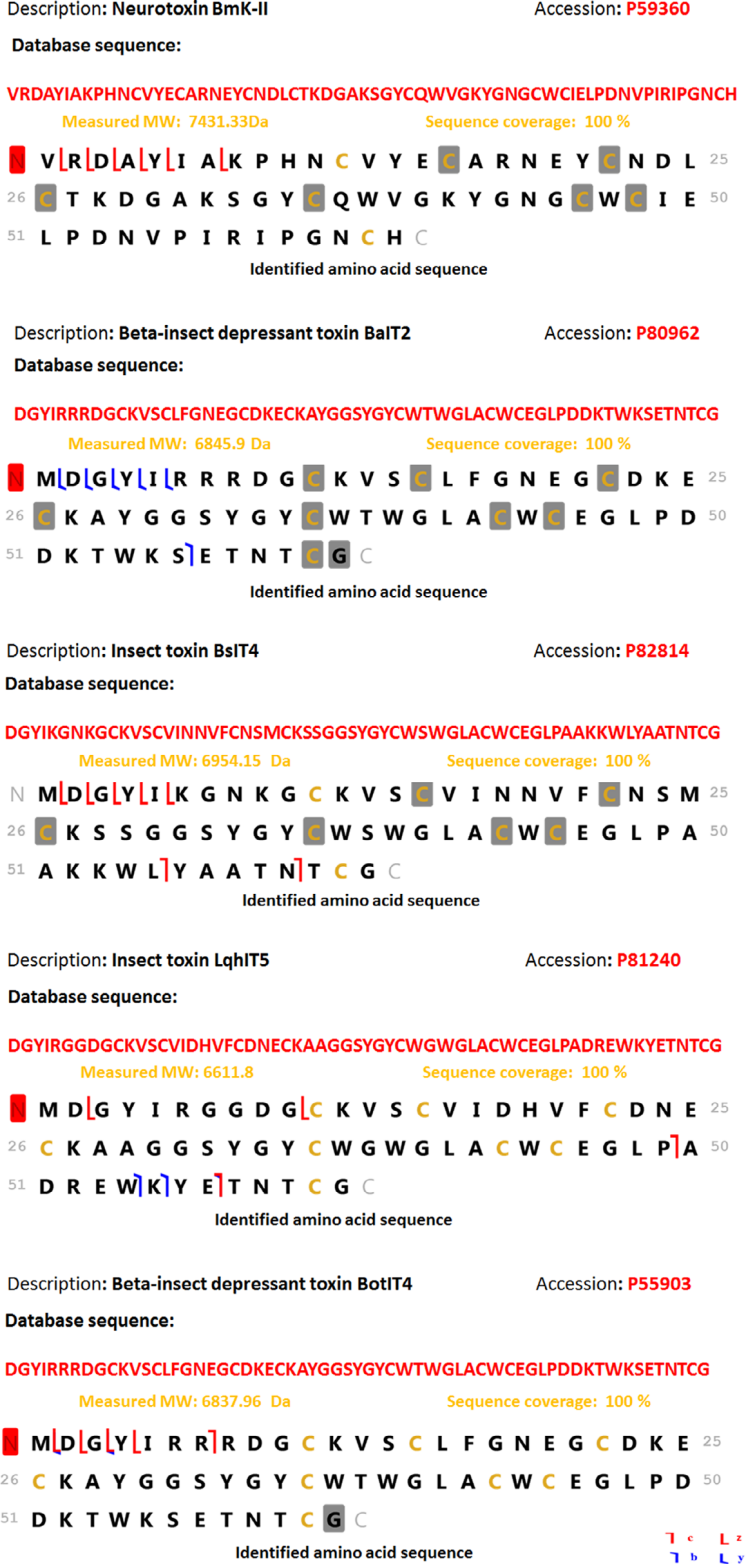
The detected amino acid sequences of the five toxins identified with 100% coverage by the top‐down LC‐MS/MS; neurotoxin BmK‐II (P59360); beta‐insect depressant toxin BaIT2 (P80962); insect toxin BsIT4 (P82814); insect toxin LqhIT5 (P81240); and beta‐insect depressant toxin BotIT4 (P55903).

The analysis of reduced/alkylated *B. occitanus* venom filtrate by tandem mass spectrometry allowed the identification of 68 peptides with a molecular weight (MW) from 1959.13 to 7943.53 Da. The detected experimental sequences are shown in Table 2; five of the entries were identified with 100% sequence coverage: neurotoxin BmK‐II (P59360), beta‐insect depressant toxin BotIT4 (P55903), beta‐insect depressant toxin BaIT2 (P80962), insect toxin LqhIT5 (P81240), and insect toxin BsIT4 (P82814). These toxins were reported for the first time in this Moroccan venom, they corresponded to toxins already identified in other scorpion venom. The determined sequence of the neurotoxin BmK‐II (P59360) showed 100% similarity with the database sequence, whereas the observed sequences of the other toxins showed methylation in the N‐terminal part compared with sequences reported in Uniprot database (Fig. 4). Therefore, the other peptides corresponded approximately to toxins, previously identified in other scorpion species with a sequence identity ranging from 17% to 98% (Fig. S2).

**Table 2:** List of the identified peptides by top‐down analysis of the reduced/alkylated *B. occitanus* venom filtrate. Data sets generated from the mass spectrometer were analyzed by the proteome discover 2.2 software, against UniProtKB/Swiss‐Prot database. The amino acids sequences colored in black were those detected by the analysis. Peptide entries in bold were identified by both top‐down and bottom‐up approaches.

Category	Accession	Description	Identified Sequence	Coverage (%)	Measured MW (Da)	No. of peptides	No. of PSMs	No. of unique peptides	No. of protein groups	No. of AAs	calc.pI
NaScTx	P59356	Alpha‐like toxin Lqh6	MVRDGYIAQPENCVYHCIPDCDTLCKDNGGTGGHCGFKLGHGIACWCNALPDNVGIIV DGVKCHK	98.46	6974.21	1	4	1	1	65	6.48
	**P13488**	Alpha‐like toxin Bom3	MGRDGYIAQPENCVYHCFPGSSGCDTLCKEKGATSGHCGFLPGSGVACWCDNLPNK VPIVVGGEKCH	98.5	7012.14	1	1	1	1	67	6.71
	P56678	Alpha‐like toxin Lqh3	MVRDGYIAQPENCVYHCFPGSSGCDTLCKEKGGTSGHCGFKVGHGLACWCNALPDNV GIIVEGEKCHS	98.52	7215.31	1	22	1	1	68	6.48
	Q9NJC4	Chain (toxin BmKaTx17) [10–73] in toxin BmKaTx17	MLLMTGVESGRDAYIAKNYNCVYHCFRDDYCNGLCTENGADSGYCYLAGKYGNACWC INLPDDKPIRIPGKCHRR	84	7062.13	1	1	1	1	75	7.58
	Q4TUA4	Chain (alpha‐toxin 4) [20–85] in alpha‐toxin 4	MNYLVFFSLALLLMTGVESVRDGYIADDKNCAYFCGRNAYCDDECKKKGAESGYCQWA GVYGNACWCYKLPDKVPIRVPGRCNGG	77.64	7218.31	1	1	0	0	85	7.5
	**P59863**	Beta‐toxin BotIT2	MDGYIKGYKGCKITCVINDDYCDTECKAEGGTYGYCWKWGLACWCEDLPDEKRWKSE TNTC	98.36	6564.78	1	1	1	1	61	4.84
	P60163	Toxin Cg2	MKDGYLVNKSTGCKYSCIENINDSHCNEECISSIRKGSYGYCYKFYCYCIGMPDSTQVYP IPGKTCSTE	88.4	6871.92	1	1	1	1	69	6.92
	P60256	Toxin Boma6b	MVRDAYIAQNYNCVYDCARDAYCNELCTKNGAKSGHCEWFGPHGDACWCIDLPNNVPI KVEGKCHRK	98.5	7307.23	1	4	1	1	67	7.2
	**O77091**	Chain(beta‐insect excitatory toxin BmK IT‐AP) [19–90] in beta‐insect excitatory toxin BmK IT‐AP	MKFFLIFLVIFPIMGVLGKKNGYAVDSSGKVAECLFNNYCNNECTKVYYADKGYCCLLKC YCFGLADDKPVLDIWDSTKNYCDVQIIDLS	80	7943.53	2	6	2	1	90	5.36
	**P21150**	Toxin AaHIT4	MEHGYLLNKYTGCKVWCVINNEECGYLCNKRRGGYYGYCYFWKLACYCQGARKSELW NYKTNKCDL	98.48	7791.58	1	6	1	1	66	8.46
	P80962	Beta‐insect depressant toxin BaIT2	MDGYIRRRDGCKVSCLFGNEGCDKECKAYGGSYGYCWTWGLACWCEGLPDDKTWKS ETNTCG	100	6845.9	1	4	1	1	62	5.31
	**P01485**	Alpha‐mammal toxin Bot3; chain (alpha‐mammal toxin Bot3) [10–73] in alpha‐mammal toxin Bot3	MLVMAGVESVKDGYIVDDRNCTYFCGRNAYCNEECTKLKGESGYCQWASPYGNACYC YKVPDHVRTKGPGRCN	87.67	7289.18	1	5	1	1	73	7.53
	Q86BW9	Chain (Makatoxin‐2) [20–83] in Makatoxin‐2	MNYLIVISFALLLMTSVESGRDAYIADSENCTYFCGSNPYCNDLCTENGAKSGYCQWAG RYGNACWCIDLPDKVPIRIPGPCRGR	75.29	7062.11	1	4	1	1	85	5.25
	G4V3T9	Neurotoxin BmK AGAP‐SYPU2	MVKDGYIVDDKNCAYFCGRNAYCDDECEKNGAESGYCQWAGVYGNACWCYKLPDKV PIRVPGRCNG	98.48	7289.18	1	6	1	1	66	5.31
	P84614	Alpha‐toxin Bs‐Tx28	MGVRDAYIADDKNCVYTCGSNSYCNTECTKNGAESGYCQWFGRWGNGCWCIKLPDKV PIRIPGKCR	98.48	7214.2	1	1	1	1	66	8.12
	Q9BLM4	Toxin AahP1005; Chain (toxin AahP1005) [20–83] in toxin AahP1005	MNYLVMISLALLFMTGVESKKDGYIVDDKNCTFFCGRNAYCNDECKKKGAESGYCQWA SPYGNACYCYKLPDRVSTKKKGGCNGR	75.29	7316.26	1	3	1	1	85	8.46
	P86408	Neurotoxin MeuNaTx‐1	MVRDGYIADDKNCAYFCGRNAYCDEECKKKGAESGYCQWAGQYGNACWCYKLPDK VPIKVSGKCN	98.46	7218.31	1	6	1	1	65	7.85
	**P60255**	Toxin Boma6a	MVRDAYIAQNYNCVYDCARDAYCNDLCTKNGAKSGYCEWFGPHGDACWCIDLPNNV PIKVEGKCHRK	98.5	7221.18	1	12	1	1	67	7.09
	P15225	Neurotoxin Os3	MGVRDGYIAQPHNCVYHCFPGSGGCDTLCKENGATQGSSCFILGRGTACWCKDLPDR VGVIVDGEKCH	98.52	6957.15	2	6	2	1	68	6.71
	P45697	Alpha‐like toxin BmK‐M1; Chain (alpha‐like toxin BmK‐M1) [20–83] in alpha‐like toxin BmK‐M1	MNYLVMISFALLLMTGVESVRDAYIAKPHNCVYECARNEYCNDLCTKNGAKSGYCQWV GKYGNGCWCIELPDNVPIRVPGKCHR	76.19	7429.4	1	4	1	1	84	7.88
	E4VP24	Chain [20–85] in sodium channel neurotoxin MeuNaTxalpha‐1	MNSLVMISLALLVMTGVESVRDGYIADDKNCAYFCGRNAYCDEECKKKGAESGYCQW AGQYGNACWCYKLPDKVPIKVSGKCNGR	77.64	7336.32	1	1	1	1	85	7.85
	**P55902**	Alpha‐insect toxin BotIT1	MVRDAYIAQNYNCVYFCMKDDYCNDLCTKNGASSGYCQWAGKYGNACWCYALPDNV PIRIPGKCHS	98.48	7345.15	1	2	1	1	66	7.55
	E7CAU3	Chain (neurotoxin BmK AGP‐SYPU1) [2–65] in neurotoxin BmK AGP‐SYPU1	MGRDAYIAQNYNCVYHCFRDDYCNGLCTENGADSGYCYLAGKYGHACW CINLPDDKPIRIPGKCHRR	98.5	7488.32	2	8	2	1	67	7.61
	Q1I178	Toxin Td9	MIGMVAECKDGYLVGDDGCKMHCFTRPGHYCASECSRVKGKDGYCYAW LACYCYNMPNWAPIWNSATNSCGKGK	86.48	7076.01	1	2	1	1	74	7.84
	A0A146CJ90	Chain [20–87] in Venom toxin meuNa32	MNYLILISFALLVITGVESARDAYIAQNYNCVYFCLNPWSSYCDDLCTKNGAK SGYCQIFGKYGNACWCIDLPDKVPIRIPGKCHFA	78.16	7690.37	1	1	1	1	87	7.53
	P68410	Alpha‐mammal toxin Ts2	MKEGYAMDHEGCKFSCFIRPAGFCDGYCKTHLKASSGYCAWPACYCYGV PDHIKVWDYATNKC	98.41	6655.84	1	6	1	1	63	7.61
	P68726	Chain (Insect toxin 2–53) [22–82] in Insect toxin 2–53	MKLLLLLIVSASMLIESLVNADGYIKRRDGCKVACLVGNEGCDKECKAYGGSY GYCWTWGLACWCEGLPDDKTWKSETNTCGGKK	71.76	6739.87	1	1	1	1	85	7.5
	Q1I163	Toxin Td8; chain (toxin Td8) [21–83] in toxin Td8	MTRFVLFLSCFFLIGMVVECKDGYLVGDDGCKMHCFTRPGHYCASECSRVK GKDGYCYAWLACYCYNMPNWAPIWNSATNRCRGRK	73.25	6986.05	1	3	1	1	86	8.34
	P56569	Makatoxin‐1	MGRDAYIADSENCTYTCALNPYCNDLCTKNGAKSGYCQWAGRYGNACWCI DLPDKVPIRISGSCR	98.46	7240.24	1	2	0	0	65	7.5
	D8UWD3	Sodium channel neurotoxin MeuNaTxalpha‐7	MARDGYIADDKNCAYFCGRNAYCDEECKKKGAESGYCQWAGQYGNACWC YKLPDKVPIKVSGKCNGR	98.5	7295.24	1	1	1	1	67	8.1
	P0DMH9	Chain (alpha‐toxin BmalphaTx47) [20–83] in alpha‐toxin BmalphaTx47	MNYLIVISFALLLMTGVQSGRDAYIADSENCTYTCALNPYCNDLCTKNGAKSG YCQWAGRYGNACWCIDLPDKVPIRISGSCRGR	75.29	7240.24	1	1	0	0	85	7.87
	P01483	Neurotoxin Bot2	MGRDAYIAQPENCVYECAKNSYCNDLCTKNGAKSGYCQWLGRWGNACYC IDLPDKVPIRIEGKCHF	98.48	7240.24	1	19	1	1	66	7.55
	**P17728**	Chain (alpha‐insect toxin LqhaIT) [20–85] in alpha‐insect toxin LqhaIT	MNHLVMISLALLLLLGVESVRDAYIAKNYNCVYECFRDAYCNELCTKNGASS GYCQWAGKYGNACWCYALPDNVPIRVPGKCHRK	77.64	7173.2	1	12	1	1	85	8.12
	P01496	Chain (toxin‐3) [15–76] in toxin‐5	MLVVVCLLTAGTEGKKDGYPVEYDNCAYICWNYDNAYCDKLCKDKKADSGY CYWVHILCYCYGLPDSEPTKTNGKCKSGKK	76.54	7105.03	1	20	1	1	81	7.49
	Q1EG64	Chain [20–85] in sodium toxin peptide BmKTb'	MNYLVMISFAFLLMTGVESARDAYIAQNYNCVYHCARDAYCNELCTKNGAKS GSCPYLGEHKFACYCKDLPDNVPIRVPGKCNGG	77.64	7321.09	1	2	1	1	85	7.58
	**P01488**	alpha‐toxin Bot1	MGRDAYIAQPENCVYECAQNSYCNDLCTKNGATSGYCQWLGKYGNACWC KDLPDNVPIRIPGKCHF	98.48	7074.14	1	3	1	1	66	6.92
	**P45698**	Chain (neurotoxin BmK‐M9) [15–78] in neurotoxin BmK‐M9	MISFALLLMTGVESVRDAYIAKPENCVYHCATNEGCNKLCTDNGAESGYCQW GGRYGNACWCIKLPDRVPIRVPGKCHR	81.01	7015.19	1	1	1	1	79	7.88
	**P83644**	Toxin Lqh4	MGVRDAYIADDKNCVYTCGANSYCNTECTKNGAESGYCQWFGKYGNACWC IKLPDKVPIRIPGKCR	98.48	7155.25	1	3	1	1	66	8.1
	P01487	Alpha‐insect toxin Lqq3	MVRDAYIAKNYNCVYECFRDSYCNDLCTKNGASSGYCQWAGKYGNACWC YALPDNVPIRVPGKCH	98.5	6980.01	2	12	2	1	65	7.87
	H1ZZI7	Toxin Tpa6	MSIFPIALALLLIGLEEGEAARDGYPLSKNNNCKIYCPDTDVCKDTCKNRASAP DGKCDGWNSCYCFKVPDHIPVWGDPGTKPCMT	74.41	7059.12	1	2	1	1	86	5.38
	B8XGY6	Chain [20–85] in Putative alpha‐toxin Tx17	MNYLILISLAVLLTSGVESVRDAYIAQNYNCVYTCFKDAYCNDLCTKNGATSGY CQWVGKYGNGCWCYALPDNVPIRVPGKCHSR	77.64	7313.2	1	2	1	1	85	7.87
	**P81504**	Insect toxin AaHIT5	MDGYIKRHDGCKVTCLINDNYCDTECKREGGSYGYCYSVGFACWCEGLPDD KAWKSETNTCD	98.38	6894.89	1	8	1	1	62	4.83
	P68722	Chain (beta‐insect excitatory toxin LqhIT1b) [19–88] in beta‐insect excitatory toxin LqhIT1b	MKFFLLFLVVLPIMGVLGKKNGYAVDSKGKAPECFLSNYCNNECTKVHYADK GYCCLLSCYCFGLNDDKKVLEISDTTKKYCDFTIIN	79.54	7924.56	1	1	1	1	88	7.87
	P60257	Toxin Boma6c	MVRDAYIAQNYNCVYTCFKDAHCNDLCTKNGASSGYCQWAGKYGNACWCY ALPDNVPIRIPGKCHRK	98.5	7308.21	2	14	2	1	67	8.31
	M1J7U4	Putative sodium channel alpha‐toxin Acra5	MVRDGYIMIKDTNCKFSCNIFKKWEYCSPLCQSKGAETGYCYNFGCWCLDL PDDVPVYGDRGVICRTR	98.52	7741.51	1	1	1	1	68	7.5
	Q9N682	Chain (neurotoxin BmK‐M11) [20–83] in neurotoxin BmK‐M11	MNYLVMISFALLLMTGVESVRDAYIAKPENCVYHCATNEGCNKLCTDNGAESG YCQWGGKYGNACWCIKLPDDVPIRVPGKCHR	77.38	7179.21	2	2	2	1	84	7.09
	P55903	beta‐insect depressant toxin BotIT4	MDGYIRRRDGCKVSCLFGNEGCDKECKAYGGSYGYCWTWGLACWCEGLPDD KTWKSETNTCG	100	6837.96	1	4	1	1	62	5.31
	A0A0K0LBU9	Chain [20–83] in sodium channel blocker AbNaTx26	MRAALLLAFSSLILTGVLTKKSGYPTQHDGCKIWCVFNHFCSNYCETYGGSGYCYT WKLACWCDNIHDWVPTWSYATTKCRAK	77.1	7505.2	1	1	1	1	83	8.31
	P0C910	Alpha‐toxin Amm3	MGRDGYIVDTKNCVYHCYPPCDGLCKKNQAKSGSCGFLYPSGLACWCVALPENV PIKDPNDDCHK	98.46	7011.14	1	1	1	1	65	7.09
	**P59360**	Neurotoxin BmK‐II	VRDAYIAKPHNCVYECARNEYCNDLCTKDGAKSGYCQWVGKYGNGCWCIELPDNV PIRIPGNCH	100	7431.33	2	14	2	1	65	7.09
	P81240	Insect toxin LqhIT5	MDGYIRGGDGCKVSCVIDHVFCDNECKAAGGSYGYCWGWGLACWCEGLPADREWK YETNTCG	100	6611,8	1	3	1	1	62	4.72
	P01497	Chain (beta‐insect excitatory toxin 1) [19–88] in beta‐insect excitatory toxin 1	MKFLLLFLVVLPIMGVFGKKNGYAVDSSGKAPECLLSNYCNNECTKVHYADKGYCCLL SCYCFGLNDDKKVLEISDTRKSYCDTTIIN	79.54	7928.54	1	10	1	1	88	7.53
	V9P3B8	Chain [23–82] in Chain [23–82] in Meutoxin‐3	MKILTVFMIFIANFLSMTQVFSLKDRFLLINGSYELCLYEENLDEDCERLCKEQNASDG FCRQPHCFCADMPDDYPTRPTTR	73.17	7074.13	1	1	1	1	82	4.75
	Q8T3T0	Depressant insect toxin BmK ITa1	MKLFLLLLISASMLIDGLVNADGYIRGSNGCKVSCLWGNEGCNKECGAYGASYGYCW TWGLACWCEGLPDDKTWKSESNTCGGKK	71.76	6632.71	1	18	1	1	85	6.38
	Q9GQW3	Chain (toxin BmKaIT1) [20–83] in toxin BmKaIT1	MNYLVMISFAFLLMTGVESVRDAYIAQNYNCVYHCARDAYCNELCTKNGAKSGSCPY LGEHKFACYCKDLPDNVPIRVPGKCHRR	75.29	7012.23	1	3	1	1	85	8.12
	Q95WX6	Beta‐insect depressant toxin BmKITb	MKLFLLLVISASMLIDGLVNADGYIRGSNGCKVSCLWGNEGCNKECKAFGAYYGYCW TWGLACWCQGLPDDKTWKSESNTCGGKK	71.76	6775.93	1	4	1	1	85	7.85
	P0C5H1	Beta‐toxin Isom1	MKKNGYAVDSSGKAPECLLSNYCNNECTKVHYADKGYCCLLSCYCFGLSDDKKVLEIS DTRKKYCDYTIIN	98.59	7895.47	1	35	1	1	71	7.53
	Q9GNG8	Toxin BmKaTX15	MNYLVFFSLALLVMTGVESVRDGYIADDKNCAYFCGRNAYCDDECKKNGAESGYCQW AGVYGNACWCYKLPDKVPIRVPGKCNGG	77.64	7211.14	1	1	1	1	85	6.4
	**M1JMR8**	Sodium channel alpha‐toxin Acra8	MVRDGYIVDDKNCTFFCGRNAYCNDECKKKGGESGYCQWASPYGNACWCYKLPDRV PIKEKGRCNGR	98.5	7218.3	1	1	1	1	67	8.29
	A0A0U4RDS7	Chain [20–87] in sodium channel toxin NaTx4	MNHLVMISLAFLFMTGVASVRDGYIAQPETCAYHCIPGSSGCYTLCKEKKGESGHCGWK SGHGSAWWCNDLPDKEGIIVDGKGCTRR	78.16	7243.29	2	4	2	1	87	7.66
	P82814	Insect toxin BsIT4	MDGYIKGNKGCKVSCVINNVFCNSMCKSSGGSYGYCWSWGLACWCEGLPAAKKWLY AATNTCG	100	6954.15	1	1	1	1	63	8.31
	B8XGX9	Chain [20–87] in Putative alpha‐toxin Tx2	MNYLIMISLALLLMTGVESGTGVRDAYIADDKNCVYTCALNSYCNTECTKNGAESGYCQ WLGQYGNACWCIKLPDRVPIRIPGKCRG	78.16	7394.28	1	3	1	1	87	7.5
	**Q17254**	Alpha‐insect toxin Bot14	MSSLMISTAMKGKAPYRQVRDGYIAQPHNCAYHCLKISSGCDTLCKENGATSGHCGH KSGHGSACWCKDLPDKVGIIVHGEKCHR	78.82	7184.3	1	5	1	1	85	8.5
KScTx	A0A059UI30	Chain (potassium channel toxin Meg‐beta‐KTx1) [28–91] in potassium channel toxin Meg‐beta‐KTx1	MQRNLVVLLFLGMVALSSCGLREKHFQKLVKYAVPEGTLRTIIQTAVHKLGKTQFGCPA YQGYCDDHCQDIKKQEGFCHGFKCKCGIPMGF	70.32	6889.3	1	9	1	1	91	8.76
	**Q9N661**	Potassium channel toxin BmTXK‐beta‐2	MQRNLVVLLFLGMVALSSCGLREKHFQKLVKYAVPEGTLRTIIQTAVHKLGKTQFGCP AYQGYCDDHCQDIKKEEGFCHGFKCKCGIPMGF	25.27	2506.46	1	1	1	1	91	8.57
AMP	A0A0A1I6E7	AMP AcrAP1	MEIKYLLTVFLVLLIVSDHCQAFLFSLIPHAISGLISAFKGRRKRDLDGQIDRFRNFRKRD AELEELLSKLPIY	24.32	1959.13	1	1	1	1	74	9.31
Myotropic neuropeptide	F8THJ9	Putative orcokinin	MMFGIWILCGTAFFFCHVDAYLEYSNMAPGYNALVRRRSMKQPSEGRMFDNLGYNQE SLVKRNFDEIDNVGFNDFGPASRPGSGRSWFPKRNWELARYNLRRLVKRATQD ELMENKRQELDEIDKSGFGGFHKRNFDEIDRSGFNDFGKRSFDRFKLVRRADFNN	16.96	3112.45	1	1	1	1	165	9.29
Hypothetical secreted protein	F1CIZ9	Hypothetical secreted protein	MQNIFWILIGVGICITAVQCDSEMESSIRDILTKRRYLKYARSVLDDLNNQLDTLHKRSC VLNLPGMDCEYGDITGSGKDQDYWTSGRTPGKKRRSYCSLGIGNSEECLTKQLKDDM TDFNSWNDKFRPGKK	25.75	3939.79	1	1	1	1	132	7.99

**Fig. 5:**
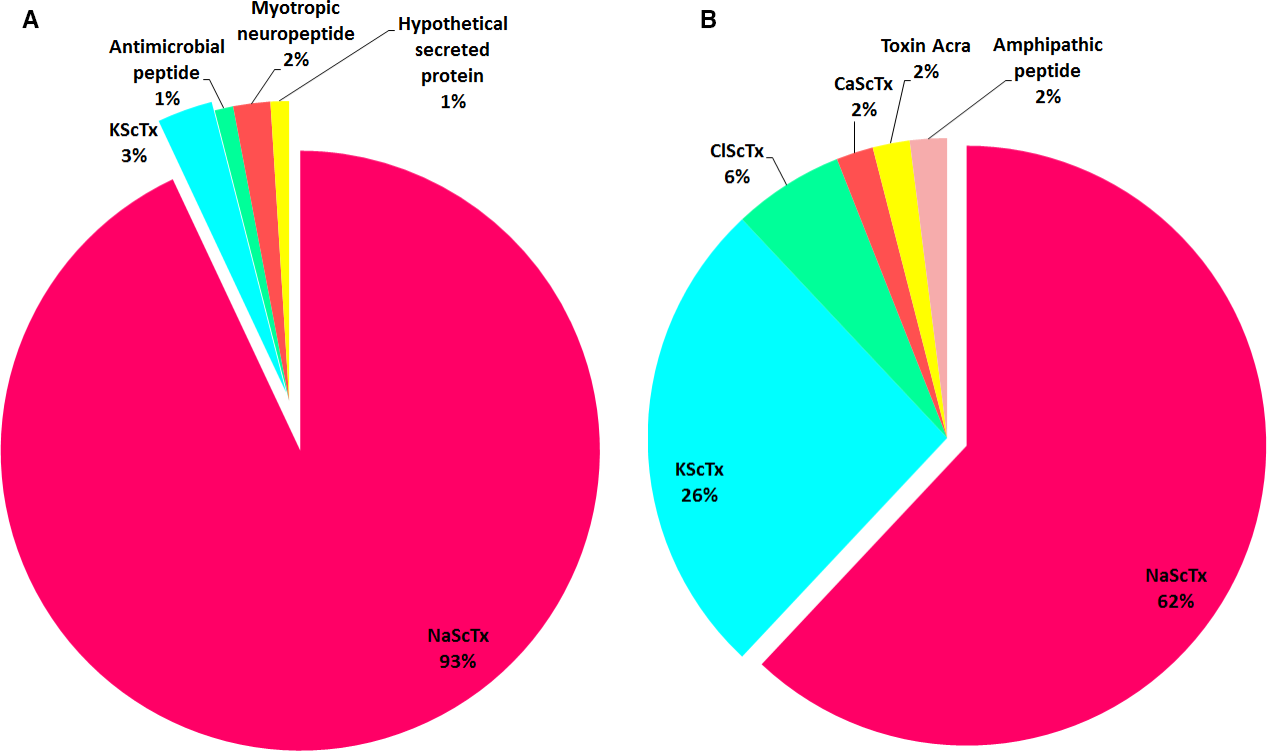
(A) Relative abundance of the different peptide categories identified in reduced/alkylated *B. occitanus* venom filtrate by the top‐down LC‐MS/MS analysis. Peptides were divided on the basis of their molecular functions into: neurotoxins active on sodium channels (NaScTxs), neurotoxins active on potassium channels (KScTxs), myotropic neuropeptide, AMP, and hypothetical secreted protein. (B) Relative abundance of the different peptide categories identified in reduced/alkylated and digested *B. occitanus* venom by bottom‐up LC‐MS/MS analysis. The peptides were divided on the basis of their molecular functions into: neurotoxins active on sodium channels (NaScTxs), neurotoxins active on potassium channels (KScTxs), neurotoxins active on chloride channels (ClScTxs), neurotoxins active on calcium channels (CaScTx), toxin Acra, and amphipathic peptide.

Therefore, the detected peptides were divided into five categories on the basis of their molecular functions according to the UniProtKB database (https://www.uniprot.org); 63 neurotoxins acting on sodium channels (NaScTxs), constitute 93% of the components and represent a MW from 6564.78 to 7943.53 Da; two neurotoxins acting on potassium channels (KScTxs) (2.94%, 2506.46–6889.3 Da); one antimicrobial peptide (AMP) (1.47%, 1959.13 Da); one myotropic neuropeptide (1.47%, 3112.45 Da); and one hypothetical secreted protein (1.47%, 3939.79 Da) (Fig. 5A).

Additionally, we have observed, that between these 68 peptides, 27 of them (40%) were detected as chains or fragments, for example, venom toxin meuNa32 (A0A146CJ90); potassium channel toxin Meg‐beta‐KTx1 (A0A059UI30); putative alpha‐toxin Tx2 (B8XGX9); sodium channel toxin NaTx4 (A0A0U4RDS7); toxin BmKaIT1(Q9GQW3); sodium channel blocker AbNaTx26 (A0A0K0LBU9); neurotoxin BmK‐M11 (Q9N682); beta‐insect excitatory toxin LqhIT1b (P68722); toxin‐5 (P01496); toxin Td8 (Q1I163); alpha‐like toxin BmK‐M1 (P45697); toxin AahP1005 (Q9BLM4); makatoxin‐2 (Q86BW9); and alpha‐mammal toxin Bot3 (P01485) (Table 2).

#### Bottom‐up proteomics

For the bottom‐up workflow, two digest methods were performed: (a) in‐solution digestion, the flow‐through containing toxin < 30 kDa was directly reduced with DTT, alkylated with IAA, and digested with trypsin; and (b) in‐gel digestion, the gel spot corresponding to peptides under 30 kDa (Fig. S1) was excised to small cubes, which after series of washings, were reduced, alkylated, and digested.

The results generated by the bottom‐up approach using the in‐gel digestion yielded the identification of 36 peptides, whereas 37 was the total of the identified peptide by in‐solution digestion. The detected peptides showed similarity of sequences with peptides from other scorpion species, and with their sequence coverage ranging from 10.23% (P68721) to 86.15% (P01489) and from 8.75% (P0C294) to 92.86% (P80669) for the in‐gel and in‐solution digestions, respectively.

The identified categories of peptides using the in‐gel digestion were as follows: 27 NaScTxs; seven KscTxs; and two ClTxs (Table 3). While, through the in‐solution digestion, we identified in addition to 24 NaScTxs, eight KScTxs and three ClScTxs, one entry that shares 60% of similarity with neurotoxin Tx‐2 (P83406) purified from *Hottentotta judaicus*, could correspond to a calcium channel activator ‘CaScTx’ scorpion. Besides neurotoxins, one amphipathic peptide was detected by this digestion method (Table 4).

**Table 3:** Bottom‐up data generated from in‐gel digestion of *B. occitanus* venom filtrate using nano‐LC‐MS/MS. Data sets generated from the mass spectrometer were analyzed by the proteome discover 2.2 software, against UniProtKB/Swiss‐Prot database.

Category	Accession	Description	Score	Coverage	No. of proteins	No. of unique peptides	No. of peptides	No. of PSMs	No. of AAs	MW [kDa]	calc. pI
NaScTx	Q86SE0	Toxin Aam2 OS = *Androctonus amoreuxi* PE = 1 SV = 1 ‐ [SCX2_ANDAM]	198.74	24.42%	9	2	3	6	86	9.3	7.87
	P21150	Toxin AaHIT4 OS = *Androctonus australis* PE = 1 SV = 1 ‐ [SIX4_ANDAU]	85.81	29.23%	2	1	2	5	65	7.8	8.46
	P13488	Alpha‐like toxin Bom3 OS = *Buthus occitanus mardochei* PE = 1 SV = 1 ‐ [SCX3_BUTOM]	169.75	56.06%	2	3	3	15	66	6.9	6.71
	P68721	Beta‐insect excitatory toxin LqhIT1a OS = *Leiurus quinquestriatus hebraeus* PE = 3 SV = 1 ‐ [SIX1A_LEIQH]	54.81	10.23%	2	1	2	3	88	9.9	8.09
	P0DJH8	Alpha‐toxin Bu1 OS = *Buthacus macrocentrus* PE = 1 SV = 1 ‐ [SCX1_BUTMA]	346.32	71.64%	1	3	5	7	67	7.5	8.48
	P86406	Neurotoxin MeuNaTx‐6 OS = *Mesobuthus eupeus* PE = 1 SV = 1 ‐ [SCXN6_MESEU]	134.56	15.15%	3	1	1	4	66	7.8	7.87
	P83644	Toxin Lqh4 OS = *Leiurus quinquestriatus hebraeus* PE = 1 SV = 1 ‐ [SCX4_LEIQH]	305.53	46.15%	8	1	3	7	65	7.2	8.1
	P01489	Alpha‐toxin Lqq4 OS = *Leiurus quinquestriatus quinquestriatus* PE = 1 SV = 1 ‐ [SCX4_LEIQU]	531.95	86.15%	9	2	5	11	65	7.2	8.1
	P01486	Alpha‐toxin Bot11 OS = *Buthus occitanus tunetanus* PE = 1 SV = 1 ‐ [SCXB_BUTOC]	106.19	35.38%	7	1	3	7	65	7.5	7.87
	P60255	Toxin Boma6a OS = *Buthus occitanus mardochei* PE = 3 SV = 1 ‐ [SCXA_BUTOM]	65.84	15.15%	2	1	1	2	66	7.5	7.09
	P17728	Alpha‐insect toxin LqhaIT OS = *Leiurus quinquestriatus hebraeus* PE = 1 SV = 2 ‐ [SCXA_LEIQH]	174.28	31.76%	4	1	2	3	85	9.6	8.12
	P04098	Neurotoxin 8 (Fragment) OS = *Buthus occitanus tunetanus* PE = 1 SV = 1 ‐ [SCX8_BUTOC]	202.83	72.22%	2	2	2	4	36	4.1	6.24
	P55902	Alpha‐insect toxin BotIT1 OS = *Buthus occitanus tunetanus* PE = 1 SV = 1 ‐ [SIX1_BUTOC]	211.59	41.54%	2	1	2	4	65	7.3	7.55
	P01488	Alpha‐toxin Bot1 OS = *Buthus occitanus tunetanus* PE = 1 SV = 2 ‐ [SCX1_BUTOC]	136.52	20.00%	1	1	1	2	65	7.3	6.92
	P81504	Insect toxin AaHIT5 OS = *Androctonus australis* PE = 1 SV = 1 ‐ [SIX5_ANDAU]	51.44	24.59%	1	1	1	2	61	6.9	4.83
	P59863	Beta‐toxin BotIT2 OS = *Buthus occitanus tunetanus* PE = 1 SV = 1 ‐ [SIX2_BUTOC]	109.66	43.33%	1	2	2	3	60	6.9	4.84
	Q17254	Alpha‐insect toxin Bot14 OS = *Buthus occitanus tunetanus* PE = 2 SV = 1 ‐ [SCXE_BUTOC]	44.99	18.82%	1	1	1	3	85	9.2	8.5
	D5HR52	Alpha‐toxin Ac3 (Fragment) OS = *Androctonus crassicauda* PE = 3 SV = 1 ‐ [SCX3A_ANDCR]	139.86	63.77%	10	2	4	10	69	7.8	7.87
	P55904	Beta‐insect depressant toxin BotIT5 OS = *Buthus occitanus tunetanus* PE = 1 SV = 1 ‐ [SIX5_BUTOC]	64.67	27.87%	21	2	2	9	61	6.8	5.31
	O77091	Beta‐insect excitatory toxin BmK IT‐AP OS = *Mesobuthus martensii* GN = IT‐AP PE = 1 SV = 1 ‐ [SIXP_MESMA]	126.26	17.78%	8	2	2	4	90	10.2	5.36
	P59864	Beta‐insect depressant toxin BotIT6 OS = *Buthus occitanus tunetanus* PE = 1 SV = 1 ‐ [SIX6_BUTOC]	32.37	11.29%	1	1	1	1	62	7.3	8.1
	P68723	Beta‐insect excitatory toxin LqhIT1c OS = *Leiurus quinquestriatus hebraeus* PE = 1 SV = 1 ‐ [SIX1C_LEIQH]	182.31	11.36%	1	2	3	8	88	9.9	8.1
	P59360	Neurotoxin BmK‐II OS = *Mesobuthus martensii* PE = 1 SV = 1 ‐ [SCX2_MESMA]	48.26	15.63%	3	1	1	1	64	7.2	7.09
	P15224	Toxin Os1 OS = *Orthochirus scrobiculosus* PE = 1 SV = 1 ‐ [SCX1_ORTSC]	39.14	19.70%	1	1	1	1	66	7.6	7.88
	D5HR50	Alpha‐toxin Ac1 (Fragment) OS = *Androctonus crassicauda* PE = 2 SV = 1 ‐ [SCX1A_ANDCR]	37.66	11.11%	2	1	1	2	81	8.7	7.55
	M1JMR8	Sodium channel alpha‐toxin Acra8 OS = *Androctonus crassicauda* PE = 3 SV = 1 ‐ [SCX8_ANDCR]	66.82	40.91%	3	2	3	5	66	7.5	8.29
	M1JBC0	Sodium channel alpha‐toxin Acra4 OS = *Androctonus crassicauda* PE = 1 SV = 1 ‐ [SCX4_ANDCR]	37.39	29.23%	1	1	2	4	65	7.1	8.31
KScTx	P0C161	Potassium channel toxin alpha‐KTx 2.8 OS = *Centruroides elegans* PE = 1 SV = 1 ‐ [KAX28_CENEL]	45.57	17.95%	2	1	1	1	39	4.3	8.94
	Q9NJC6	Potassium channel toxin BmTXK‐beta OS = *Mesobuthus martensii* PE = 2 SV = 1 ‐ [KBX2_MESMA]	264.78	23.33%	2	1	2	6	90	10.4	8.82
	P59869	Potassium channel toxin alpha‐KTx 5.4 OS = *Mesobuthus tamulus* PE = 1 SV = 1 ‐ [KAX54_MESTA]	40.7	22.58%	2	1	2	2	31	3.5	8.02
	B8XH40	Potassium channel toxin BuTXK‐beta OS = *Buthus occitanus israelis* PE = 2 SV = 1 ‐ [KBX1_BUTOS]	298.65	42.86%	2	2	5	18	91	10.2	8.57
	Q9N661	Potassium channel toxin BmTXK‐beta‐2 OS = *Mesobuthus martensii* PE = 2 SV = 1 ‐ [KBX1_MESMA]	230.62	42.86%	2	1	4	13	91	10.2	8.57
	B3EWX9	Potassium channel toxin alpha‐KTx 9.11 OS = *Mesobuthus gibbosus* PE = 1 SV = 1 ‐ [KAX9B_MESGB]	85.33	40.74%	4	1	1	2	27	2.9	5.01
	B8XH42	Potassium channel toxin alpha‐KTx 16.6 OS = *Buthus occitanus israelis* PE = 2 SV = 1 ‐ [KA166_BUTOS]	23.25	12.07%	1	1	1	1	58	6.5	8.12
ClScTx	P45639	Chlorotoxin OS = *Leiurus quinquestriatus quinquestriatus* PE = 1 SV = 1 ‐ [CTXL_LEIQU]	41.56	38.89%	1	1	1	3	36	4	8.13
	P86436	Chlorotoxin‐like peptide OS = *Androctonus australis* PE = 1 SV = 1 ‐ [CTXL_ANDAU]	290.9	44.12%	1	1	1	7	34	3.6	8.34

Underlined peptide entries were identified by in‐gel and in‐solution digestion methods.

**Table 4:** Bottom‐up data generated from in‐solution digestion of *B. occitanus* venom filtrate using nano‐LC‐MS/MS. Data sets generated from the mass spectrometer were analyzed by the proteome discover 2.2 software, against UniProtKB/Swiss‐Prot database.

Category	Accession	Description	Score	Coverage	No. of proteins	No. of unique peptides	No. of peptides	No. of PSMs	No. of AAs	MW (kDa)	calc. pI
NaScTxs	Q86SE0	Toxin Aam2 OS = *Androctonus amoreuxi* PE = 1 SV = 1 ‐ [SCX2_ANDAM]	250.68	24.42%	8	2	4	15	86	9.3	7.87
	P21150	Toxin AaHIT4 OS = *Androctonus australis* PE = 1 SV = 1 ‐ [SIX4_ANDAU]	192.23	30.77%	2	2	3	12	65	7.8	8.46
	P01482	Alpha‐toxin Amm5 OS = Androctonus mauretanicus mauretanicus PE = 1 SV = 1 ‐ [SCX5_ANDMA]	96.57	28.13%	1	1	1	2	64	7.3	7.5
	P01481	Alpha‐mammal toxin Lqq5 OS = *Leiurus quinquestriatus quinquestriatus* PE = 1 SV = 1 ‐ [SCX5_LEIQU]	77.71	25.00%	2	1	2	4	64	7.3	8.1
	P13488	Alpha‐like toxin Bom3 OS = *Buthus occitanus mardochei* PE = 1 SV = 1 ‐ [SCX3_BUTOM]	155.6	59.09%	2	2	4	18	66	6.9	6.71
	P45698	Neurotoxin BmK‐M9 OS = *Mesobuthus martensii* PE = 1 SV = 1 ‐ [SCX9_MESMA]	124.54	26.58%	11	1	3	15	79	8.8	7.88
	P68721	Beta‐insect excitatory toxin LqhIT1a OS = *Leiurus quinquestriatus hebraeus* PE = 3 SV = 1 ‐ [SIX1A_LEIQH]	55.48	10.23%	2	1	2	4	88	9.9	8.09
	P0DJH8	Alpha‐toxin Bu1 OS = *Buthacus macrocentrus* PE = 1 SV = 1 ‐ [SCX1_BUTMA]	272.84	71.64%	1	2	4	9	67	7.5	8.48
	P83644	Toxin Lqh4 OS = *Leiurus quinquestriatus hebraeus* PE = 1 SV = 1 ‐ [SCX4_LEIQH]	293.2	46.15%	7	1	3	10	65	7.2	8.1
	P01489	Alpha‐toxin Lqq4 OS = *Leiurus quinquestriatus quinquestriatus* PE = 1 SV = 1 ‐ [SCX4_LEIQU]	569.9	90.77%	8	2	6	18	65	7.2	8.1
	P01486	Alpha‐toxin Bot11 OS = *Buthus occitanus tunetanus* PE = 1 SV = 1 ‐ [SCXB_BUTOC]	76.63	35.38%	2	1	3	7	65	7.5	7.87
	P60255	Toxin Boma6a OS = *Buthus occitanus mardochei* PE = 3 SV = 1 ‐ [SCXA_BUTOM]	46.01	15.15%	2	1	1	2	66	7.5	7.09
	P17728	Alpha‐insect toxin LqhaIT OS = *Leiurus quinquestriatus hebraeus* PE = 1 SV = 2 ‐ [SCXA_LEIQH]	369.61	51.76%	4	2	5	13	85	9.6	8.12
	P04098	Neurotoxin 8 (Fragment) OS = Buthus occitanus tunetanus PE = 1 SV = 1 ‐ [SCX8_BUTOC]	536.34	77.78%	2	3	3	13	36	4.1	6.24
	P55902	Alpha‐insect toxin BotIT1 OS = *Buthus occitanus tunetanus* PE = 1 SV = 1 ‐ [SIX1_BUTOC]	296.35	61.54%	1	1	3	9	65	7.3	7.55
	P01488	Alpha‐toxin Bot1 OS = *Buthus occitanus tunetanus* PE = 1 SV = 2 ‐ [SCX1_BUTOC]	185.35	20.00%	1	1	1	3	65	7.3	6.92
	P81504	Insect toxin AaHIT5 OS = *Androctonus australis* PE = 1 SV = 1 ‐ [SIX5_ANDAU]	49.42	24.59%	1	1	1	2	61	6.9	4.83
	P01485	Alpha‐mammal toxin Bot3 (Fragment) OS = *Buthus occitanus tunetanus* PE = 1 SV = 2 ‐ [SCX3_BUTOC]	436.17	61.11%	3	2	5	61	72	8.1	7.53
	P59863	Beta‐toxin BotIT2 OS = *Buthus occitanus tunetanus* PE = 1 SV = 1 ‐ [SIX2_BUTOC]	164.41	41.67%	1	2	2	4	60	6.9	4.84
	Q17254	Alpha‐insect toxin Bot14 OS = *Buthus occitanus tunetanus* PE = 2 SV = 1 ‐ [SCXE_BUTOC]	91.78	18.82%	1	1	1	9	85	9.2	8.5
	O77091	Beta‐insect excitatory toxin BmK IT‐AP OS = *Mesobuthus martensii* GN = IT‐AP PE = 1 SV = 1 ‐ [SIXP_MESMA]	50.93	17.78%	8	1	2	5	90	10.2	5.36
	P59864	Beta‐insect depressant toxin BotIT6 OS = *Buthus occitanus tunetanus* PE = 1 SV = 1 ‐ [SIX6_BUTOC]	78.83	53.23%	1	2	3	7	62	7.3	8.1
	P0C294	Toxin Acra I‐3 OS = *Androctonus crassicauda* PE = 2 SV = 1 ‐ [TX13_ANDCR]	43.58	8.75%	1	1	1	1	80	8.8	8.25
	P59360	Neurotoxin BmK‐II OS = *Mesobuthus martensii* PE = 1 SV = 1 ‐ [SCX2_MESMA]	62.88	15.63%	3	1	1	2	64	7.2	7.09
KScTxs	P0CC12	Potassium channel toxin alpha‐KTx 8.5 OS = Odontobuthus doriae PE = 1 SV = 1 ‐ [KAX85_ODODO]	86.25	48.28%	2	1	1	2	29	3.2	5.1
	P83407	Potassium channel toxin alpha‐KTx 19.1 OS = *Mesobuthus martensii* PE = 1 SV = 1 ‐ [KA191_MESMA]	82.22	32.26%	1	1	1	5	31	3.3	8.73
	Q95NJ8	Potassium channel toxin alpha‐KTx 17.1 OS = *Mesobuthus martensii* PE = 1 SV = 1 ‐ [KA171_MESMA]	79.79	16.36%	1	1	1	5	55	6.2	8
	P80669	Potassium channel toxin alpha‐KTx 9.3 OS = *Leiurus quinquestriatus hebraeus* PE = 1 SV = 1 ‐ [KAX93_LEIQH]	211.78	92.86%	3	2	2	9	28	3	6.98
	Q9NJC6	Potassium channel toxin BmTXK‐beta OS = *Mesobuthus martensii* PE = 2 SV = 1 ‐ [KBX2_MESMA]	135.05	27.78%	2	2	2	3	90	10.4	8.82
	Q9N661	Potassium channel toxin BmTXK‐beta‐2 OS = *Mesobuthus martensii* PE = 2 SV = 1 ‐ [KBX1_MESMA]	96.9	42.86%	3	2	4	7	91	10.2	8.57
	P86399	Neurotoxin lamda‐MeuTx OS = *Mesobuthus eupeus* PE = 1 SV = 2 ‐ [TXL_MESEU]	262	25.00%	2	1	1	8	64	7.2	7.12
	P80670	Toxin GaTx2 OS = *Leiurus quinquestriatus hebraeus* PE = 1 SV = 1 ‐ [KAX83_LEIQH]	86.02	48.28%	2	1	1	2	29	3.2	5.1
CaScTxs	P83406	Neurotoxin Tx‐2 OS = Buthotus judaicus PE = 1 SV = 1 ‐ [SCBT2_BUTJU]	287.63	60.71%	1	2	2	10	28	2.9	4.89
ClScTxs	P86436	Chlorotoxin‐like peptide OS = *Androctonus australis* PE = 1 SV = 1 ‐ [CTXL_ANDAU]	993.18	67.65%	1	3	3	65	34	3.6	8.34
	P45639	Chlorotoxin OS = *Leiurus quinquestriatus quinquestriatus* PE = 1 SV = 1 ‐ [SCXL_LEIQU]	588.38	38.89%	1	2	2	38	36	4	8.13
	P01498	Neurotoxin P2 OS = Androctonus mauretanicus mauretanicus PE = 1 SV = 1 ‐ [SCXP_ANDMA]	188.48	71.43%	1	2	2	5	35	3.7	7.88
Amphipathic peptide	B8XH50	Amphipathic peptide Tx348 OS = *Buthus occitanus israelis* PE = 2 SV = 1 ‐ [NDB5R_BUTOS]	87.27	19.40%	4	1	1	1	67	7.8	9.19

Underlined peptide entries were identified by in‐gel and in‐solution digestion methods.

According to the results, 23 of the entries were detected by both digestion methods (Tables 3 and 4). Thus, 14 peptides were identified only by the in‐solution digestion method, for example, alpha‐toxin Amm5 (P01482), alpha‐mammal toxin Bot3 (P01485), potassium channel toxin alpha‐KTx 9.3 (P80669), neurotoxin Tx‐2 (P83406), neurotoxin P2 (P01498), and amphipathic peptide Tx348 (B8XH50). Otherwise, regarding the in‐gel digestion results, 13 peptides were identified only by this method of digestion, for example, potassium channel toxin alpha‐KTx 9.11 (B3EWX9); sodium channel alpha‐toxin Acra4 (M1JBC0); sodium channel alpha‐toxin Acra8 (M1JMR8), alpha‐toxin Ac3 (fragment) (D5HR52); and beta‐insect depressant toxin BotIT5 (P55904).

Since the aim of using two methods of digestions was to identify the maximum of peptide, the data generated by bottom‐up approaches using in‐gel and in‐solution digestions were then summarized in Table 5; the repeated molecules were deleted and thus allowed the detection of a total of 50 peptides, which were divided into different categories according to their molecular functions. The generated data from the bottom‐up process confirmed that the family with the most diverse members in this venom is neurotoxins, with 31 NaScTxs (62%, 4.3–10.2 kDa), 13 KScTxs (26%, 2.9–10.4 kDa), three ClScTxs (6%, 3.6–4 kDa), one CaScTx (2%, 2.9 kDa), and one toxin Acra (2%, 8.8 kDa).

In addition to these neurotoxins, we identified one amphipathic peptide (2%, 7.8 kDa) (Fig. 5B). Also, some peptides were detected as fragments (10% of total): alpha‐toxin Ac1 (D5HR50) and Ac3 (D5HR52); alpha‐mammal toxin Bot3 (P01485); and neurotoxin 8 (P04098).

**Table 5:** List of the 50 peptides detected by the bottom‐up analysis of the reduced/alkylated *B. occitanus* venom filtrate. Data sets generated from the mass spectrometer were analyzed by the proteome discover 2.2 software, against UniProtKB/Swiss‐Prot database.

Category	Accession	Description	MW (kDa)	Species	Digestion method
NaScTx	P86406	Neurotoxin MeuNaTx‐6	7.8	*Mesobuthus eupeus*	In‐gel digestion
	**P59863**	Beta‐toxin BotIT2	6.9	*Buthus occitanus tunetanus*	Both
	D5HR52	Alpha‐toxin Ac3 (Fragment)	7.8	*Androctonus crassicauda*	In‐gel digestion
	P55904	Beta‐insect depressant toxin BotIT5	6.8	*Buthus occitanus tunetanus*	In‐gel digestion
	**O77091**	Beta‐insect excitatory toxin BmK IT‐AP	10.2	*Mesobuthus martensii*	Both
	P68723	Beta‐insect excitatory toxin LqhIT1c	9.9	*Leiurus quinquestriatus hebraeus*	In‐gel digestion
	**P59360**	Neurotoxin BmK‐II	7.2	*Mesobuthus martensii*	Both
	P15224	Toxin Os1	7.6	*Orthochirus scrobiculosus*	In‐gel digestion
	D5HR50	Alpha‐toxin Ac1 (Fragment)	8.7	*Androctonus crassicauda*	In‐gel digestion
	**M1JMR8**	Sodium channel alpha‐toxin Acra8	7.5	*Androctonus crassicauda*	Both
	M1JBC0	Sodium channel alpha‐toxin Acra4	7.1	*Androctonus crassicauda*	In‐gel digestion
	Q86SE0	Toxin Aam2	9.3	*Androctonus amoreuxi*	Both
	**P21150**	Toxin AaHIT4	7.8	*Androctonus australis*	Both
	P01482	Alpha‐toxin Amm5	7.3	*Androctonus mauretanicus mauretanicus*	In‐solution digestion
	P01481	Alpha‐mammal toxin Lqq5	7.3	*Leiurus quinquestriatus quinquestriatus*	In‐solution digestion
	**P13488**	Alpha‐like toxin Bom3	6.9	*Buthus occitanus mardochei*	Both
	**P45698**	Neurotoxin BmK‐M9	8.8	*Mesobuthus martensii*	In‐solution digestion
	P68721	Beta‐insect excitatory toxin LqhIT1a	9.9	*Leiurus quinquestriatus hebraeus*	Both
	P0DJH8	Alpha‐toxin Bu1	7.5	*Buthacus macrocentrus*	Both
	**P83644**	Toxin Lqh4	7.2	*Leiurus quinquestriatus hebraeus*	Both
	P01489	Alpha‐toxin Lqq4	7.2	*Leiurus quinquestriatus quinquestriatus*	Both
	P01486	Alpha‐toxin Bot11	7.5	*Buthus occitanus tunetanus*	In‐solution digestion
	**P60255**	Toxin Boma6a	7.5	*Buthus occitanus mardochei*	Both
	**P17728**	Alpha‐insect toxin LqhaIT	9.6	*Leiurus quinquestriatus hebraeus*	Both
	P04098	Neurotoxin 8 (Fragment)	4.1	*Buthus occitanus tunetanus*	Both
	**P55902**	Alpha‐insect toxin BotIT1	7.3	*Buthus occitanus tunetanus*	Both
	**P01488**	Alpha‐toxin Bot1	7.3	*Buthus occitanus tunetanus*	Both
	**P81504**	Insect toxin AaHIT5	6.9	*Androctonus australis*	Both
	**P01485**	Alpha‐mammal toxin Bot3 (Fragment)	8.1	*Buthus occitanus tunetanus*	In‐solution digestion
	P83406	Neurotoxin Tx‐2	2.9	*Buthotus judaicus*	In‐solution digestion
	**Q17254**	Alpha‐insect toxin Bot14	9.2	*Buthus occitanus tunetanus*	Both
	P59864	Beta‐insect depressant toxin BotIT6	7.3	*Buthus occitanus tunetanus*	In‐solution digestion
	P0C294	Toxin Acra I‐3	8.8	*Androctonus crassicauda*	In‐solution digestion
KScTx	B3EWX9	Potassium channel toxin alpha‐KTx 9.11	2.9	*Mesobuthus gibbosus*	In‐gel digestion
	P0C161	Potassium channel toxin alpha‐KTx 2.8	4.3	*Centruroides elegans*	In‐gel digestion
	B8XH42	Potassium channel toxin alpha‐KTx 16.6	6.5	*Buthus occitanus israelis*	Both
	P0CC12	Potassium channel toxin alpha‐KTx 8.5	3.2	*Odontobuthus doriae*	In‐solution digestion
	P59869	Potassium channel toxin alpha‐KTx 5.4	3.5	*Mesobuthus tamulus*	In‐gel digestion
	B8XH40	Potassium channel toxin BuTXK‐beta	10.2	*Buthus occitanus israelis*	In‐gel digestion
	Q95NJ8	Potassium channel toxin alpha‐KTx 17.1	6.2	*Odontobuthus doriae*	In‐solution digestion
	P83407	Potassium channel toxin alpha‐KTx 19.1	3.3	*Mesobuthus martensii*	in‐solution digestion
	P80669	Potassium channel toxin alpha‐KTx 9.3	3	*Leiurus quinquestriatus hebraeus*	In‐solution digestion
	P86399	Neurotoxin lamda‐MeuTx	7.2	*Mesobuthus eupeus*	In‐solution digestion
	Q9NJC6	Potassium channel toxin BmTXK‐beta	10.4	*Mesobuthus martensii*	Both
	**Q9N661**	Potassium channel toxin BmTXK‐beta‐2	10.2	*Mesobuthus martensii*	Both
ClScTx	P01498	Neurotoxin P2	3.7	*Androctonus mauretanicus mauretanicus*	in‐solution digestion
	P86436	Chlorotoxin‐like peptide	3.6	*Androctonus australis*	Both
	P45639	Chlorotoxin	4	*Leiurus quinquestriatus quinquestriatus*	Both
	P80670	Toxin GaTx2	3.2	*Leiurus quinquestriatus hebraeus*	In‐solution digestion
Amphipathic peptide	B8XH50	Amphipathic peptide Tx348	7.8	*Buthus occitanus israelis*	In‐solution digestion

Peptide entries in bold were identified by both top‐down and bottom‐up approaches.

As we mentioned above, we aimed to gain a deeper understanding of the *B. occitanus* peptidome (under 30 kDa), so the molecular diversity of its toxins. In this context, we combined data from the top‐down and bottom‐up analyses and then analyzed the generated data to infer a global and comprehensive characterization of this venom.

According to this study, a total of 118 peptides were identified from *B. occitanus* venom; among them, 16 were identified by both approaches, for example, potassium channel toxin BmTXK‐beta‐2 (Q9N661); toxin AaHIT4 (P21150); and alpha‐mammal toxin Bot3 (Fragment) (P01485).

**Fig. 6:**
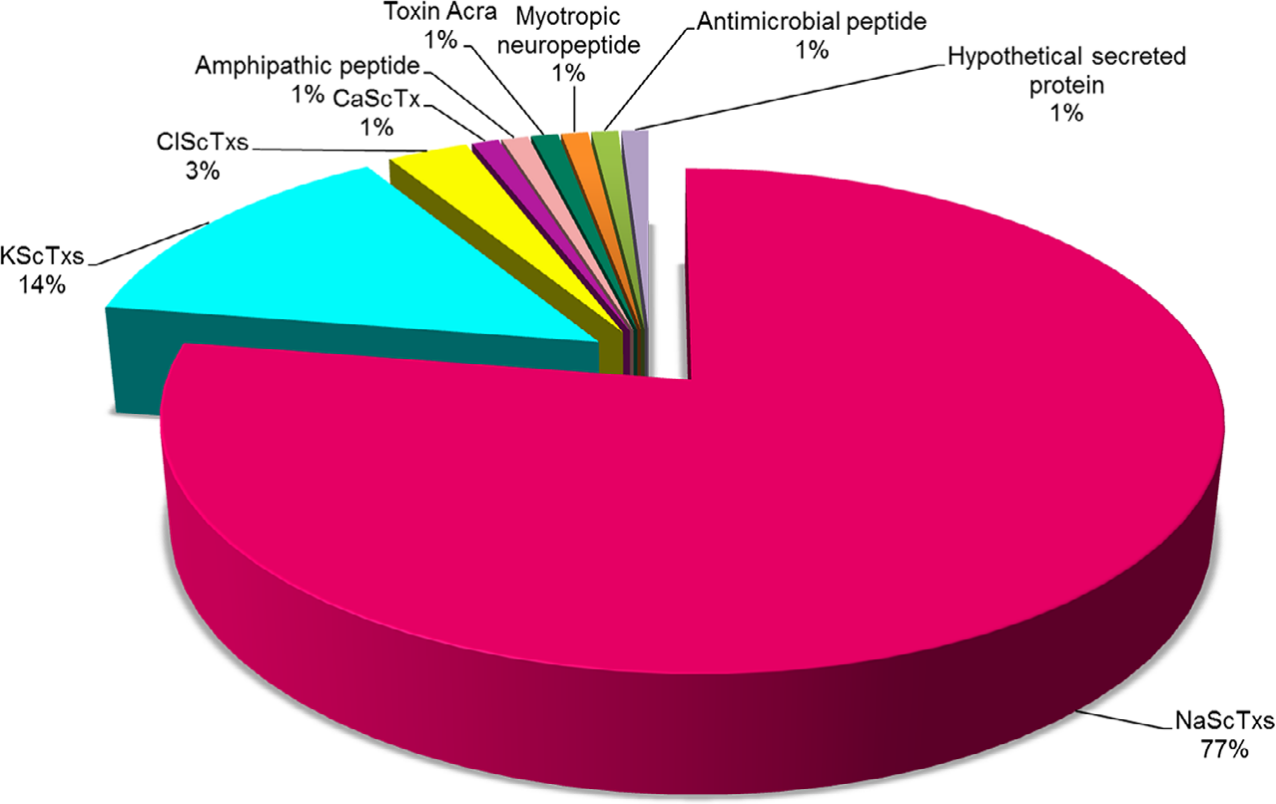
Summary of the total peptides identified by top‐down and bottom‐up approaches. The 102 peptides were divided into neurotoxins, including NaScTxs, KScTxs, ClScTxs, CaScTx and toxin Acra, amphipathic peptide, myotropic neuropeptide, AMPs, and hypothetical secreted protein.

Among the 102 identified peptides, the most representative category is neurotoxins, mainly NaScTxs (77%), followed by KScTxs (14%), ClScTxs (3%), CaScTx (1%), and toxin Acra (1%). We also characterized other peptides with low percentage such as AMPs (1%), amphipathic peptides (1%), hypothetical secreted proteins (1%), and myotropic neuropeptides (1%) (Fig. 6).

**Fig. 7:**
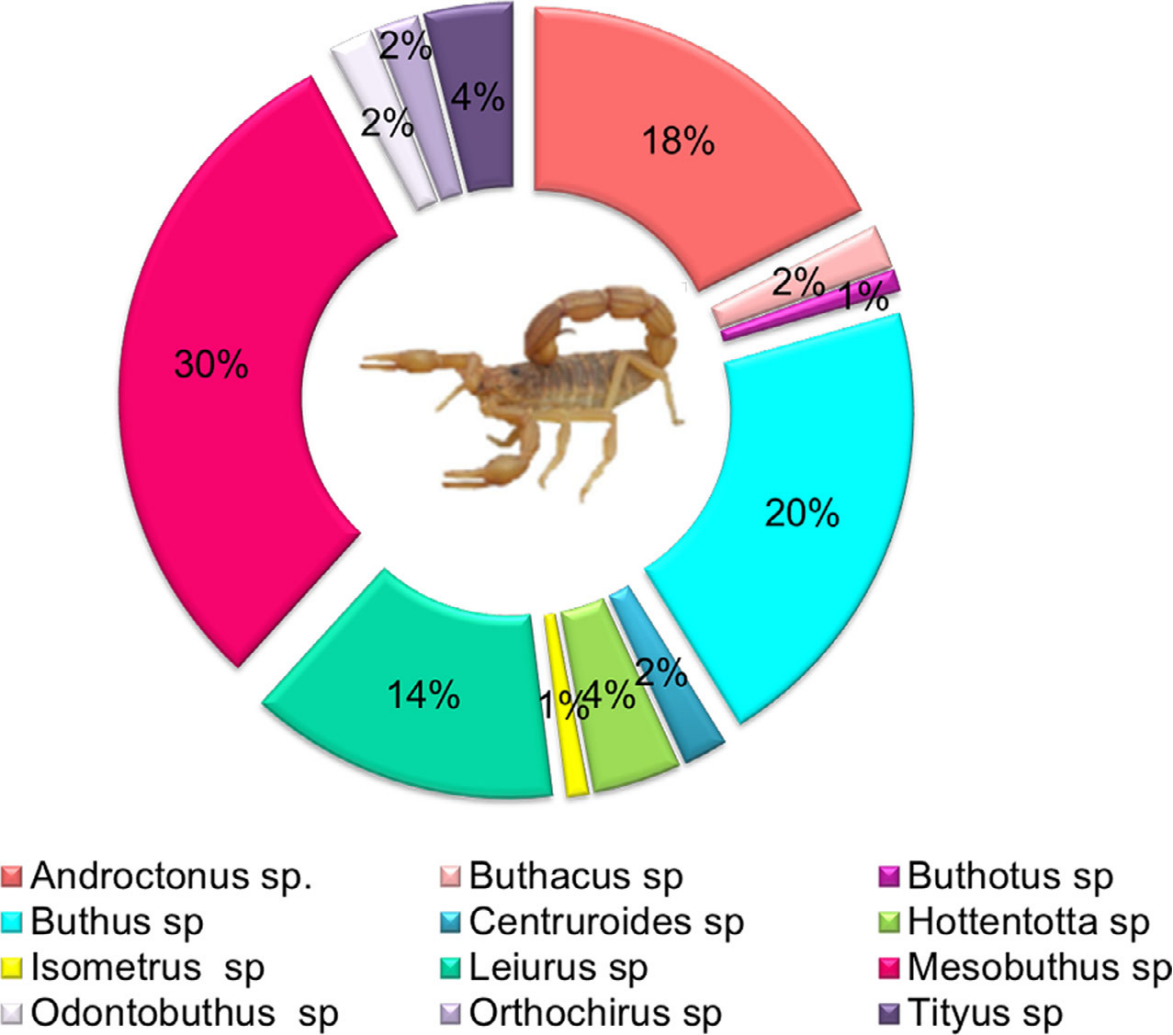
Percentage of *B. occitanus* peptides, which showed similarity of sequences with others from several scorpion genera.

The majority of described peptides were identified for the first time in this Moroccan *B. occitanus* scorpion venom. The identified peptides showed sequence similarities with toxins previously detected from several genera of scorpions (Fig. 7), principally Mesobuthus sp (30%), Buthus Sp (20%), and Androctonus sp (18%).

## Discussion

Envenomation following scorpion stings constitutes one of the most encountered emergencies in large parts of the world, especially in North Africa, where the data show the highest incidence and lethality [1]. Morocco is a country known for a high risk of envenomation owing to its huge and diversified scorpion fauna. Among the different scorpion species living in this country, the yellow scorpion *B. occitanus* is one of the most dangerous species with venom responsible for severe cases of envenomation.

Due to the limited knowledge about the composition and toxin arsenal of *B. occitanus* venom, we aimed in this study to elaborate the first exhaustive view of this scorpion venom peptidome and its molecular diversity, using mass spectrometry‐based top‐down and bottom‐up approaches.

Top‐down data sets showed that the venom of *B. occitanus* is very complex, counting around 200 MWs ranging from 1868 to 16 720 Da. A similar number of components have been revealed by previous studies [32, 33, 34], others showed fewer components, as well as *Leiurus abdullahbayrami* (45 masses) and *Opisthacanthus elatus* (106 masses) [35, 36], whereas some other scorpion venoms were more complex, such as the *Pandinus cavimanus* (390 masses) and *Centruroides limpidus* (395 masses) [37, 38]. Additionally, the repartition of MWs showed that < 1% were components with molecular masses < 2000 Da, 14% were those from 2000 to 5000 Da, 74% were those between 5000 and 8000 Da, and 10% were those over than 8000 Da, while the repartition of MW from the French *B. occitanus* scorpion venom showed an abundance of molecules ranging from 2000 to 3000 Da and those less than 2000 Da [39]. Most importantly, the whole sequences of five toxins were identified with 100% sequence coverage using the top‐down approach. These neurotoxins were detected for the first time in this venom; they all belong to the NaScTxs category and shared high similarities of sequence with toxins identified from other scorpion species: neurotoxin BmK‐II (P59360), beta‐insect depressant toxin BotIT4 (P55903), beta‐insect depressant toxin BaIT2 (P80962), insect toxin LqhIT5 (P81240), and insect toxin BsIT4 (P82814). It is important to stress that the observed sequence of the P59360 entry with a MW of 7431.33 Da showed 100% similarity with the sequence of neurotoxin BmK‐II isolated from the Chinese scorpion *Mesobuthus martensii*, this neurotoxin is active in mammal and insect Nav channel [40]. In contrast, the detected sequence of the P81240 entry (6611.8 Da) showed the presence of methionine in the N‐terminal compared with the database sequence of the Insect toxin LqhIT5, an excitatory insect beta‐toxin from the *Leiurus hebraeus* scorpion [41]. Similar to the P82814 entry (6954.15 Da), in which the observed sequence corresponds 100% to the insect toxin BsIT4, a depressant insect beta‐toxins was isolated from *Hottentotta tamulus sindicus* [42]. Also, the observed sequence of the peptide corresponding to the depressant toxin BotIT4 (6837. 96 Da) presents methionine in N‐terminal compared with the database sequence. This toxin, identified for the first time from the Tunisian *Buthus tunetanus* [43], showed also 100% sequence identity with the P80962 entry (6845.9 Da), referred to the beta‐insect depressant toxin BaIT2 isolated from the *Buthacus arenicola* scorpion [44]. The high similarity of the amino acid sequence, in both detected depressant toxins and in the other peptides is commonly observed in scorpion toxins.

Interestingly, the combined top‐down and bottom‐up data sets of *B. occitanus* venom provide the identification of 102 different peptides, whereas 147 proteins were characterized from the yellow Brazilian scorpion *Tityus serrulatus*, 60 of which were detected by the top‐down approach [45]. The major representative category of components identified in our venom was neurotoxins, mainly NaScTxs (77%), these neurotoxins are abundant in species from the Buthidae family [38, 46, 47] and less representative in scorpions from the non‐Buthidae family [33, 48, 49]. Those toxins are the ones responsible for envenomation symptoms [39]; their high content in the *B. occitanus* venom could explain the involvement of this scorpion in lethal cases of envenoming in the country.

Between the entries corresponding to NaScTxs, there are alpha‐like toxins, this type of toxins had been already identified in several Buthus sp; yet, the alpha‐toxin Bot1 (P01488) has never been found in other Moroccan Buthus subspecies except from *Buthus mardochei* [39, 50, 51, 52, 53], but identified herein with a high sequence coverage (98.48% on top‐down data set). We should mention also that we identified for the first time, in this scorpion venom, peptides corresponding to atypical NaScTxs, as well as makatoxin‐1, fragment from makatoxin‐2, toxin Cg2, chain [20‐87] in venom toxin meuNa32, and AaHIT4 toxin (which could bind on receptor site 3 or 4 of sodium channel) [33].

Besides NaScTxs and KScTxs (14%), ClScTxs (3%) were identified, these categories of peptides showed activities against autoimmune disease and cancers, respectively [54, 55, 56, 57, 58]; also, we identified one entry that shared 60% of similarity with neurotoxin Tx‐2 (P83406), a calcium channel activator identified for the first time from the *Buthotus judaicus*, this category of toxins was identified in few scorpion species, for example, *Parabuthus transvaalicus* (Kurtoxin) and *Parabuthus granulatus* (Kurtoxin‐like I) but never been detected in a Moroccan scorpion venom [59, 60]. And last but not least, peptides referring to toxin Acra category have also been screened in *B. occitanus* venom, these toxins probably acting on ion channels.

Some peptides with antibacterial activities were also found, for example, amphipathic peptide (B8XH50) and AMP AcrAP1 (A0A059UI30); this category was commonly present in scorpion venom due to its role in the protection of venom glands and its involvement in the neurotoxic effects [61, 62, 63, 64, 65]. Additionally, other components were identified with a low percentage, such as orcokinin, a myotropic neuropeptide identified from crustaceans, insects, and arachnids [17, 66], and hypothetical secreted proteins, which are proteins with unknown activities. Finally, we notice that some of the detected toxins were identified as fragments and chains, which may be due to the proteolysis of toxins. This process seems to be a usual PTM in scorpion and snake venoms, whereas its biological pertinence remains obscure [17, 45].

This study decrypted the peptidome arsenal of the Moroccan *B. occitanus* scorpion venom through proteomic view without the *de novo* sequence annotation. These findings constitute a step forward to a ‘deeper’ understanding of this scorpion venom; nevertheless, complete identification of this complex matrix is still a challenging task, especially with the lack of a specific database and/or a complete sequenced genome of this venom.

## Conclusion

Herein; we reported the first proteomic study of the Moroccan *B. occitanus* scorpion peptidome, using mass spectrometry‐based top‐down and bottom‐up venomic approaches. The combination of these approaches allowed the identification of 102 components classified, with approximation, on different categories, mainly neurotoxins (96%), including NaScTxs (77%), KScTxs (14%), ClScTxs (3%), CaScTx (1%), and toxin Acra (1%). We also identified AMPs (1%), amphipathic peptides (1%), hypothetical secreted proteins (1%), and myotropic neuropeptides (1%). This study constitutes for sure a step forward to a deeper understanding of the *B. occitanus* venom; nevertheless, complete identification of this complex matrix is still a challenging task, especially with the lack of a specific database and a complete sequenced genome.

## Conflict of interest

The authors declare no conflict of interest.

## Author contributions

NO and JCR conceived the research. KD and CM performed experiments. KD and CM analyzed the data. KD interpreted data and wrote the manuscript. AL, BD, and SC participated in writing. JMS and RC reviewed the manuscript. NO designed the project, supervised the study, and reviewed the manuscript. All authors read and approved the final version for publication.

## Supporting information


**Fig. S1.** SDS/PAGE profile of the < 30 kDa filtrate of *Buthus occitanus* venom. Molecular weight markers (MM) are indicated in kDa. Proteins/Peptides were stained with Coomassie Brilliant Blue R (InstantBlue, Expedeon, CA, USA). Stained bands corresponding to proteins/peptides with massed < 30 kDa were manually excised into equal small cubes of 1 mm^3^ and subjected to a nanoLC‐MS/MS analysis.

**Fig. S2.** Detected amino acid sequences of the 68 peptides identified by Top‐down approach.

## Data Availability

All generated data during this study are included in this article.
