# Supplementary material for: Mass spectrometry‐based top‐down and bottom‐up approaches for proteomic analysis of the Moroccan Buthus occitanus scorpion venom
Source: FEBS Open Bio. 2021 May 28;11(7):1867–92. doi: 10.1002/2211-5463.13143 (PMC8255848; doi:10.1002/2211-5463.13143)
Supplement: Supplementary file 1 — Fig. S1. SDS/PAGE profile of the < 30 kDa filtrate of Buthus occitanus venom. Molecular weight markers (MM) are indicated in kDa. Proteins/Peptides were stained with Coomassie Brilliant Blue R (InstantBlue, Expedeon, CA, USA). Stained bands corresponding to proteins/peptides with massed < 30 kDa were manually excised into equal small cubes of 1 mm3 and subjected to a nanoLC‐MS/MS analysis. [file FEB4-11-1867-s002.pptx]

## Slide 1
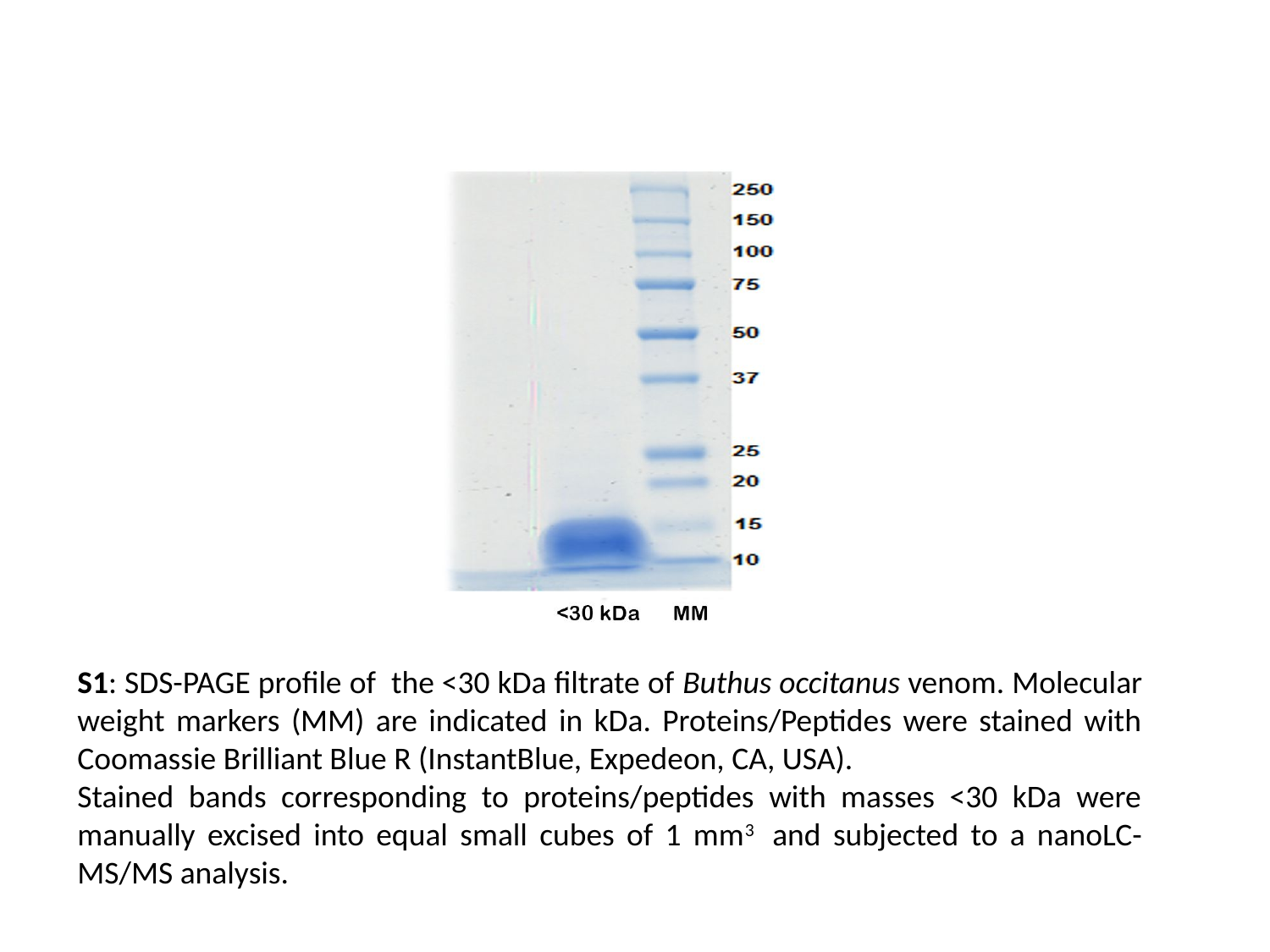

S1: SDS-PAGE profile of the <30 kDa filtrate of Buthus occitanus venom. Molecular weight markers (MM) are indicated in kDa. Proteins/Peptides were stained with Coomassie Brilliant Blue R (InstantBlue, Expedeon, CA, USA).
Stained bands corresponding to proteins/peptides with masses <30 kDa were manually excised into equal small cubes of 1 mm3 and subjected to a nanoLC-MS/MS analysis.
