# Supplementary material for: Mass spectrometry‐based top‐down and bottom‐up approaches for proteomic analysis of the Moroccan Buthus occitanus scorpion venom
Source: FEBS Open Bio. 2021 May 28;11(7):1867–92. doi: 10.1002/2211-5463.13143 (PMC8255848; doi:10.1002/2211-5463.13143)
Supplement: Supplementary file 2 — Fig. S2. Detected amino acid sequences of the 68 peptides identified by Top‐down approach. [file FEB4-11-1867-s001.pptx]

## Slide 1
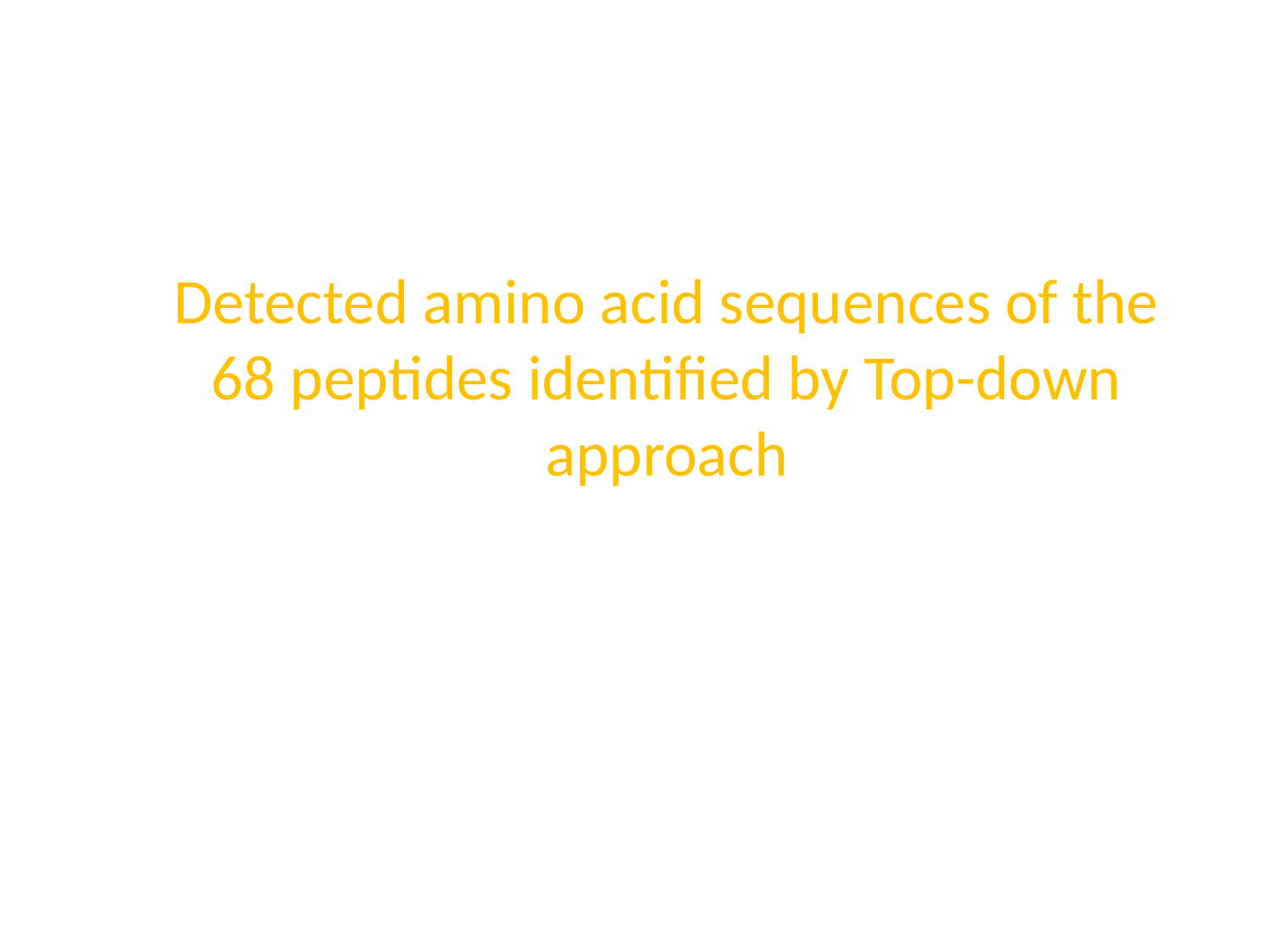

Detected amino acid sequences of the 68 peptides identified by Top-down approach

## Slide 2
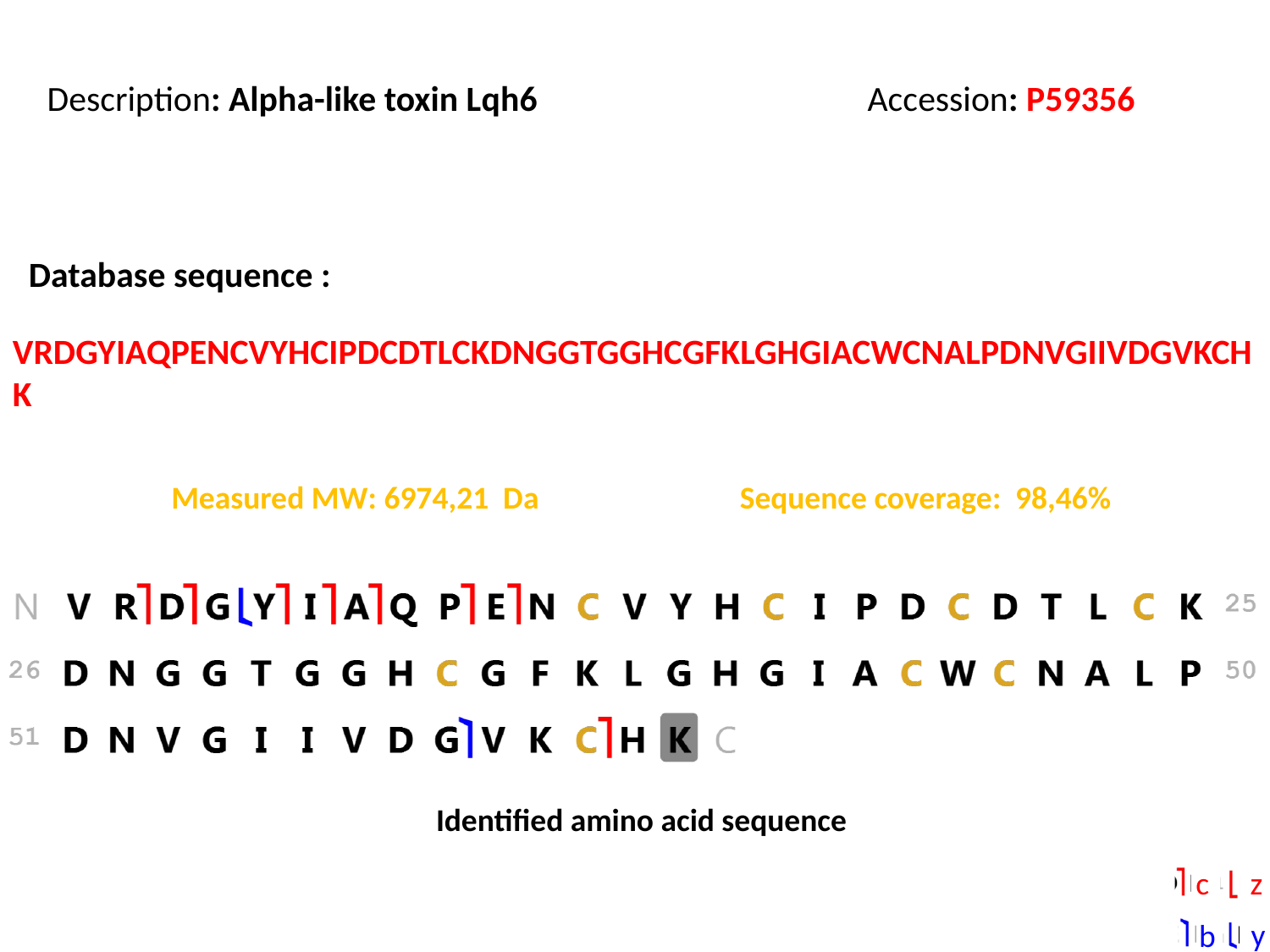

Description: Alpha-like toxin Lqh6 Accession: P59356
Database sequence :
VRDGYIAQPENCVYHCIPDCDTLCKDNGGTGGHCGFKLGHGIACWCNALPDNVGIIVDGVKCHK
Measured MW: 6974,21 Da Sequence coverage: 98,46%
Identified amino acid sequence
c
z
y
b

## Slide 3
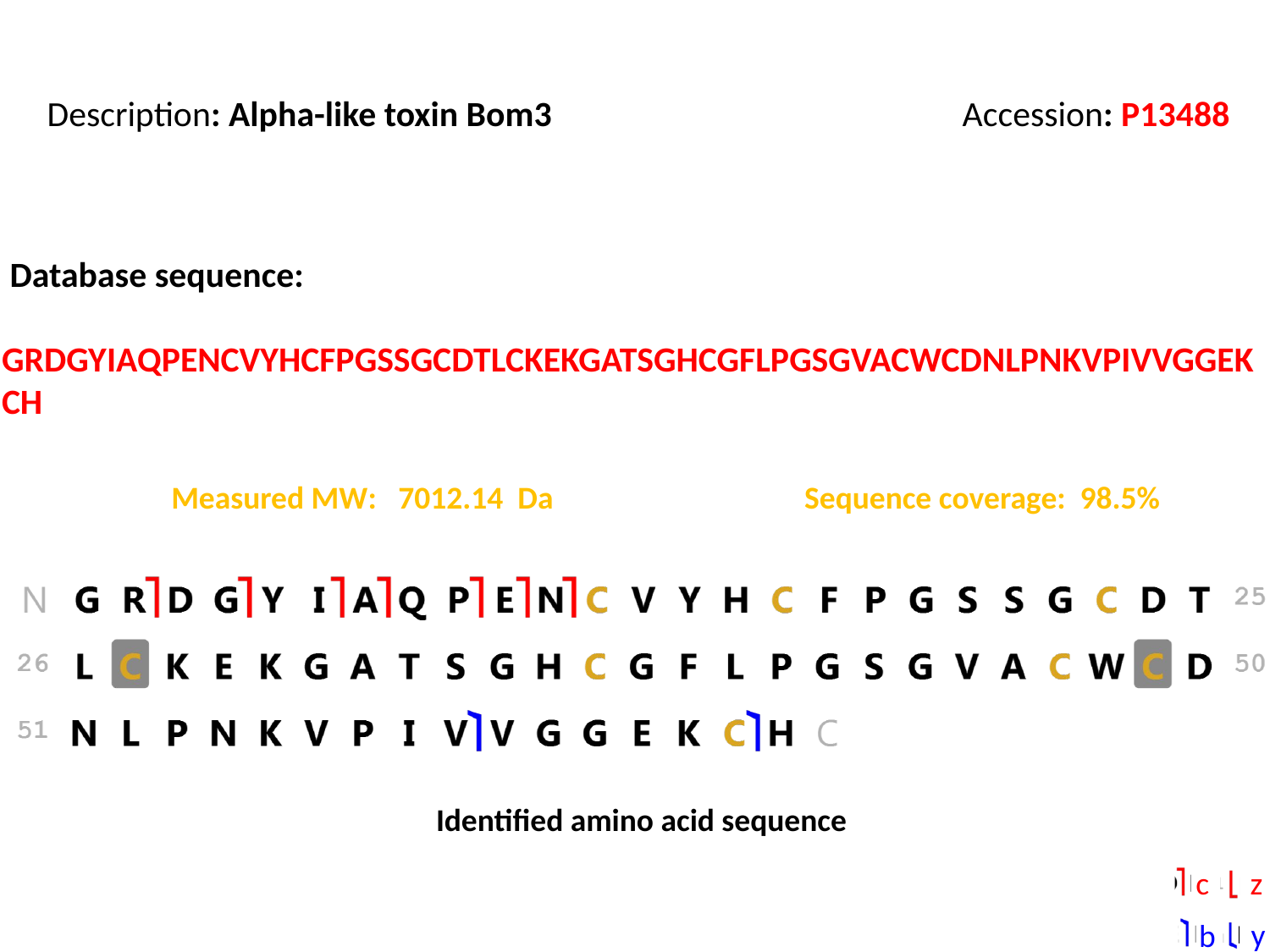

Description: Alpha-like toxin Bom3 Accession: P13488
 Database sequence:
GRDGYIAQPENCVYHCFPGSSGCDTLCKEKGATSGHCGFLPGSGVACWCDNLPNKVPIVVGGEKCH
Measured MW: 7012.14 Da Sequence coverage: 98.5%
Identified amino acid sequence
c
z
y
b

## Slide 4
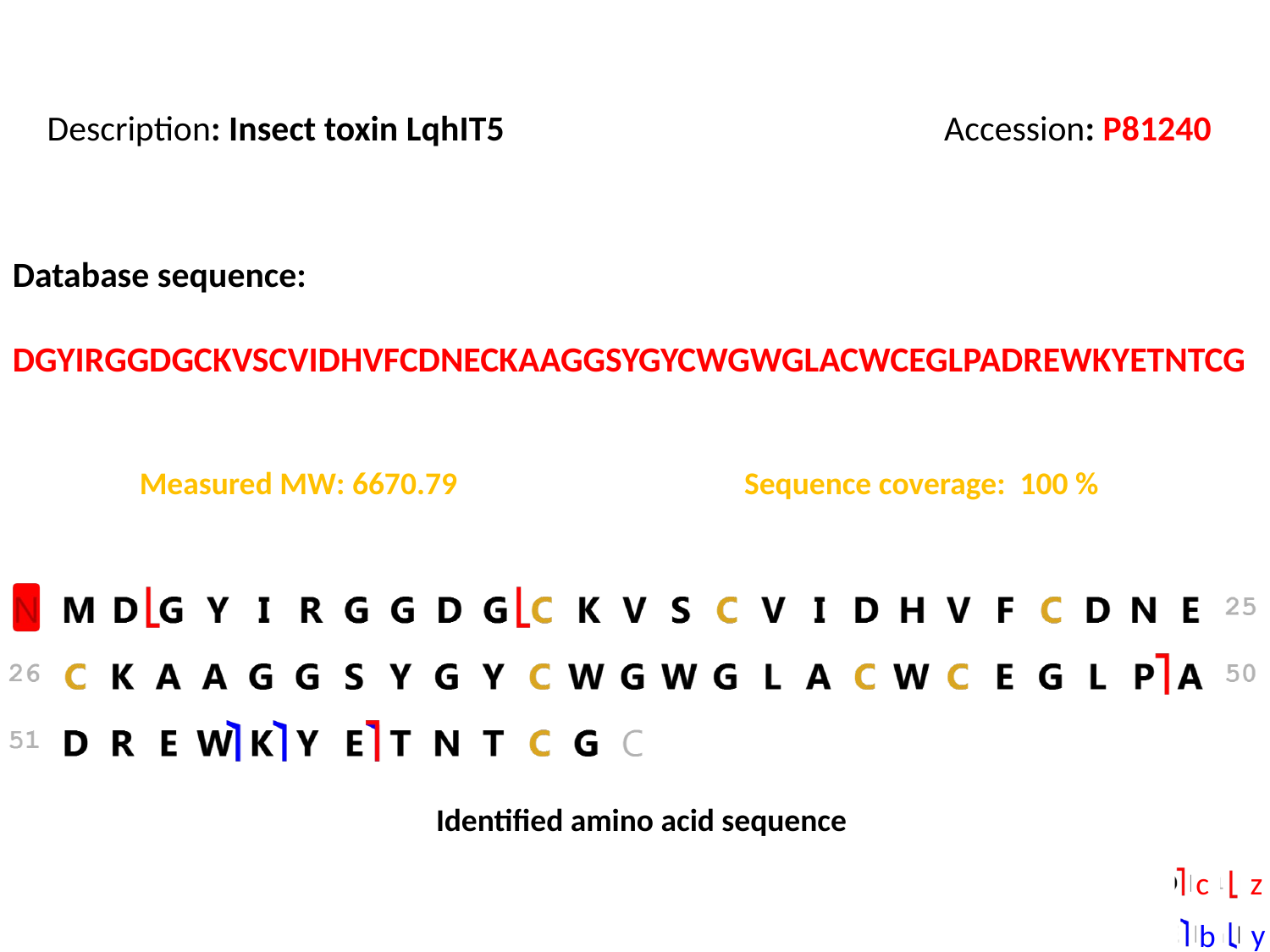

Description: Insect toxin LqhIT5 				 Accession: P81240
Database sequence:
DGYIRGGDGCKVSCVIDHVFCDNECKAAGGSYGYCWGWGLACWCEGLPADREWKYETNTCG
Measured MW: 6670.79 Sequence coverage: 100 %
Identified amino acid sequence
c
z
y
b

## Slide 5
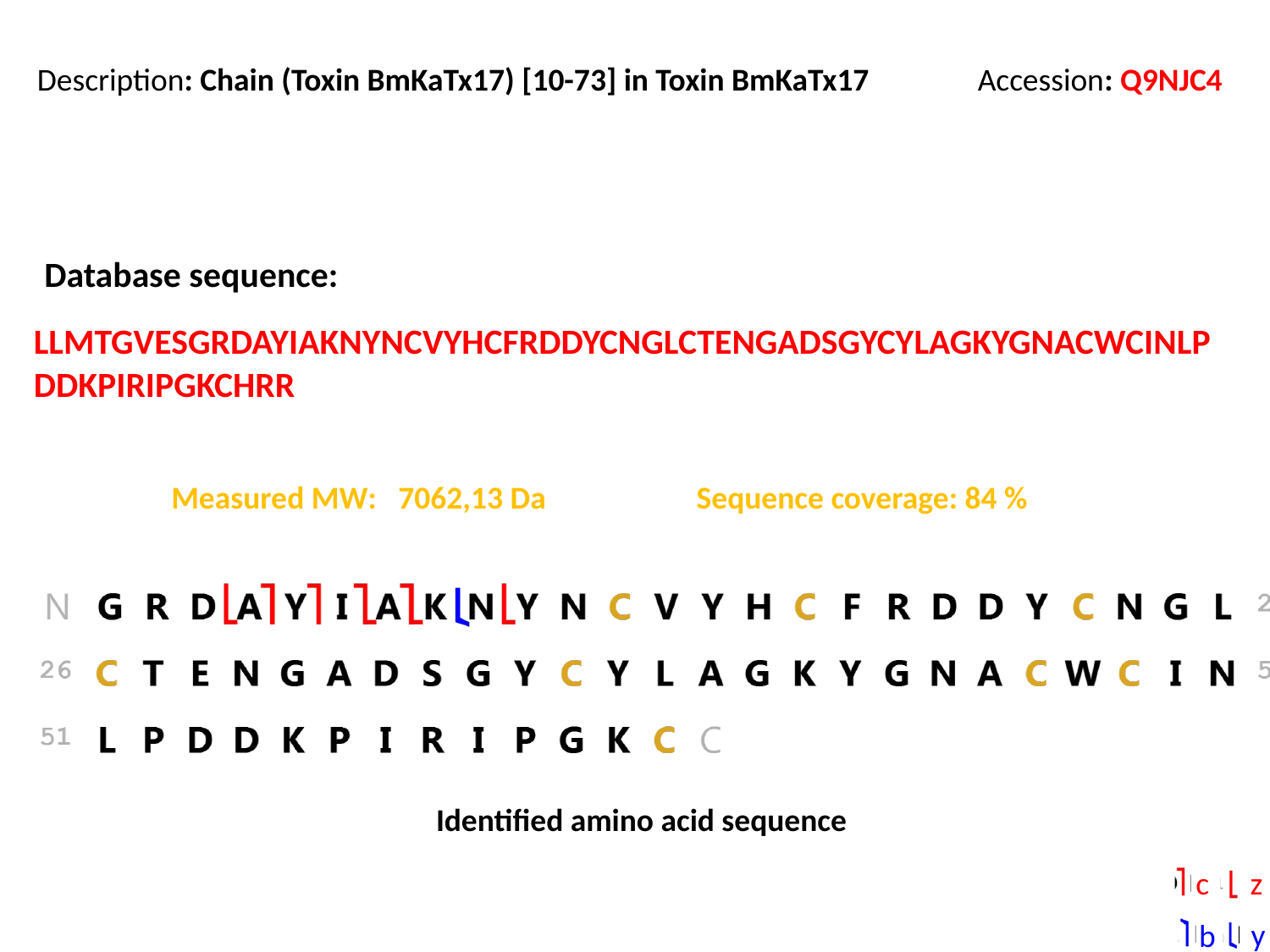

Description: Chain (Toxin BmKaTx17) [10-73] in Toxin BmKaTx17 Accession: Q9NJC4
Database sequence:
LLMTGVESGRDAYIAKNYNCVYHCFRDDYCNGLCTENGADSGYCYLAGKYGNACWCINLPDDKPIRIPGKCHRR
Measured MW: 7062,13 Da Sequence coverage: 84 %
Identified amino acid sequence
c
z
y
b

## Slide 6
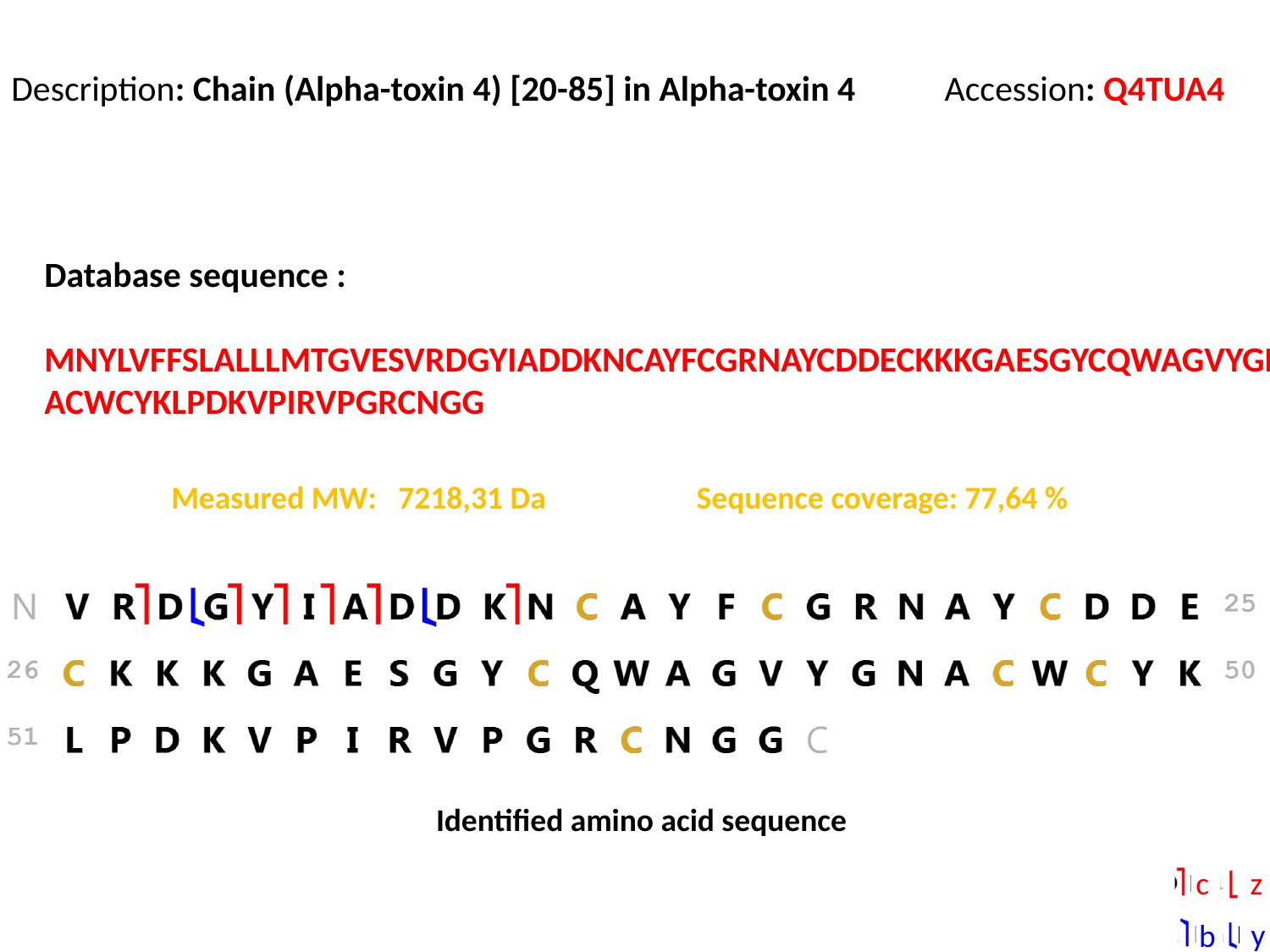

Description: Chain (Alpha-toxin 4) [20-85] in Alpha-toxin 4 Accession: Q4TUA4
Database sequence :
MNYLVFFSLALLLMTGVESVRDGYIADDKNCAYFCGRNAYCDDECKKKGAESGYCQWAGVYGNACWCYKLPDKVPIRVPGRCNGG
Measured MW: 7218,31 Da Sequence coverage: 77,64 %
Identified amino acid sequence
c
z
y
b

## Slide 7
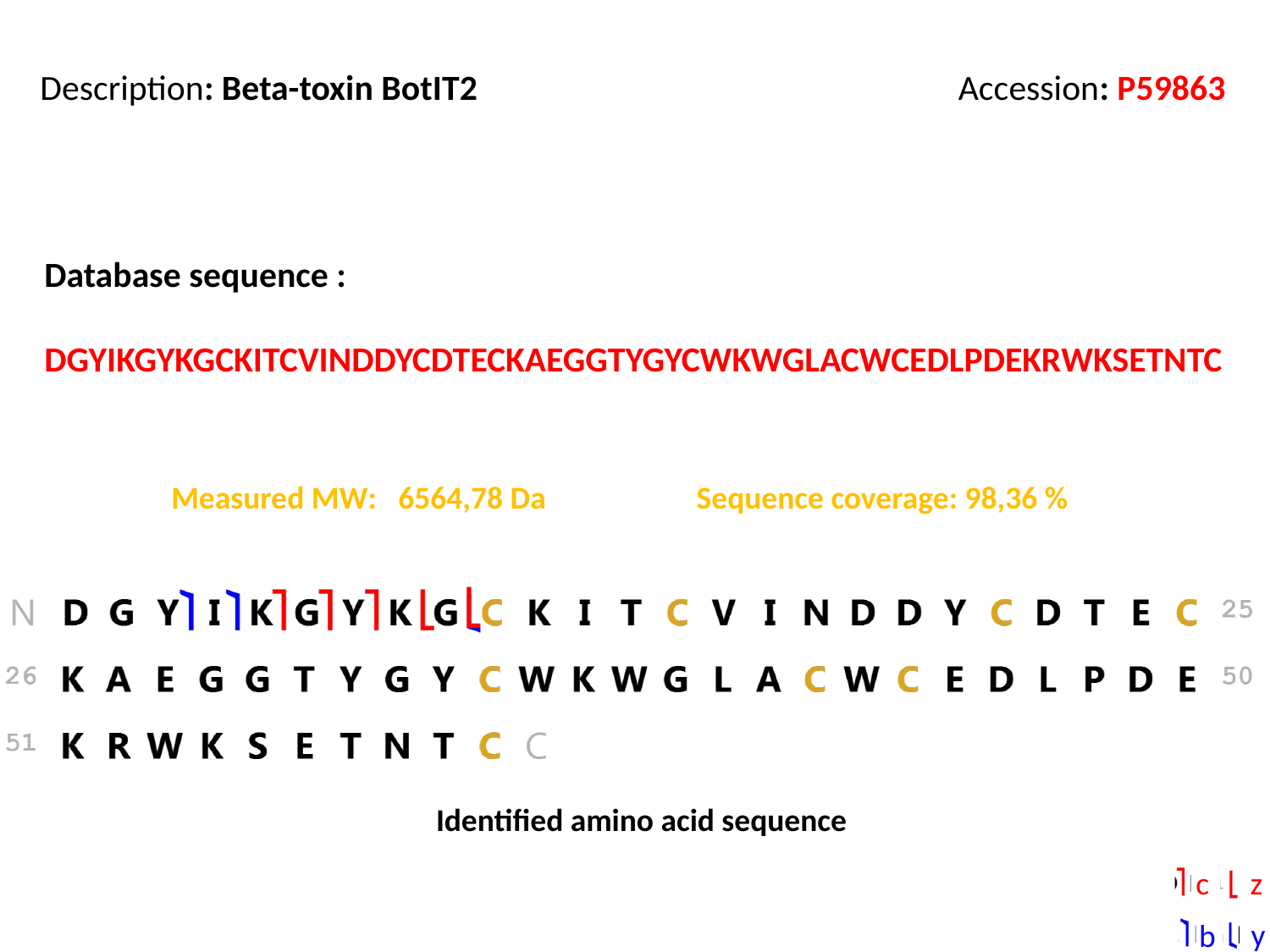

Description: Beta-toxin BotIT2	 Accession: P59863
Database sequence :
DGYIKGYKGCKITCVINDDYCDTECKAEGGTYGYCWKWGLACWCEDLPDEKRWKSETNTC
Measured MW: 6564,78 Da Sequence coverage: 98,36 %
Identified amino acid sequence
c
z
y
b

## Slide 8
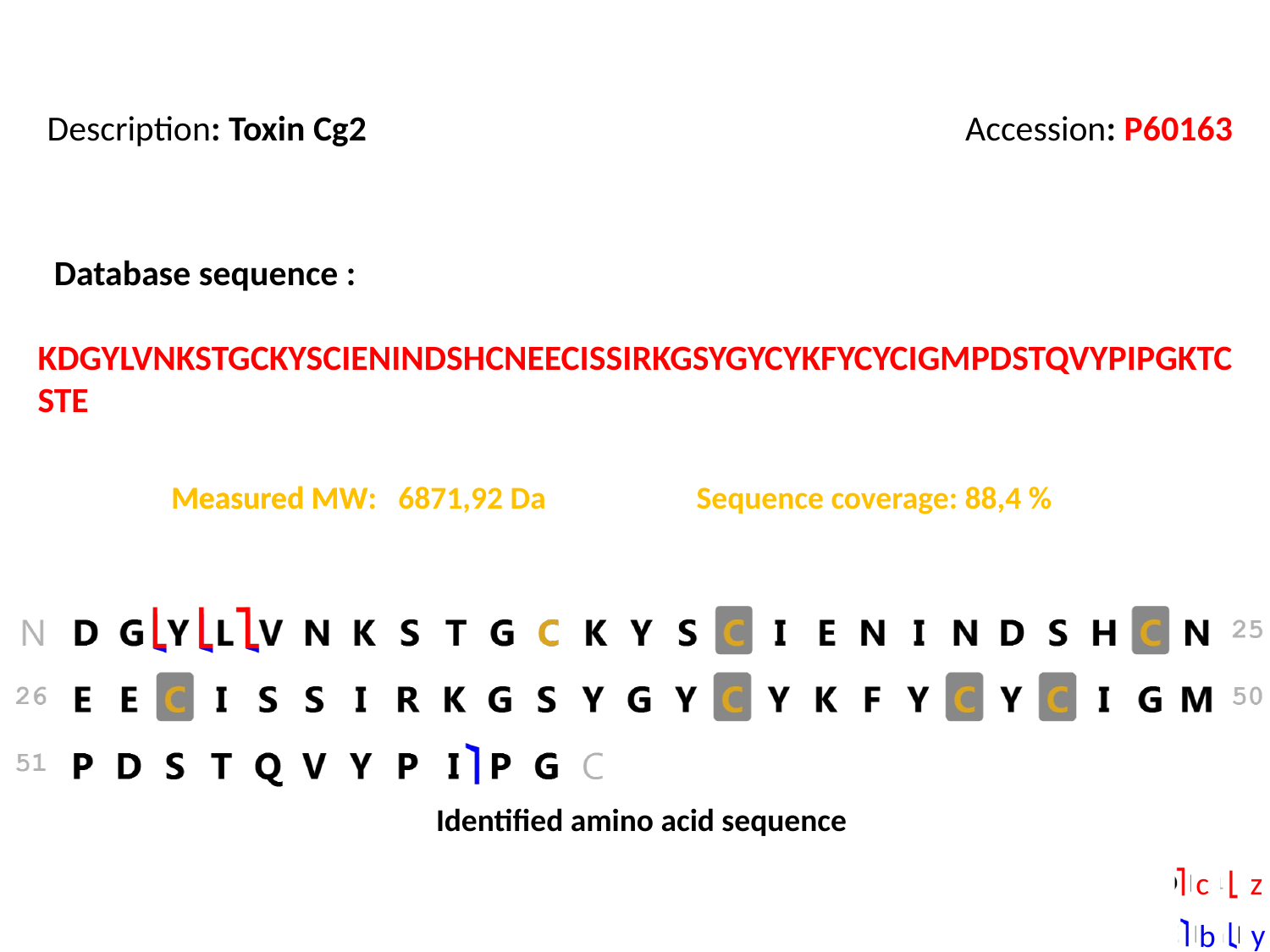

Description: Toxin Cg2		 Accession: P60163
 Database sequence :
KDGYLVNKSTGCKYSCIENINDSHCNEECISSIRKGSYGYCYKFYCYCIGMPDSTQVYPIPGKTCSTE
Measured MW:
Measured MW: 6871,92 Da Sequence coverage: 88,4 %
Identified amino acid sequence
c
z
y
b

## Slide 9
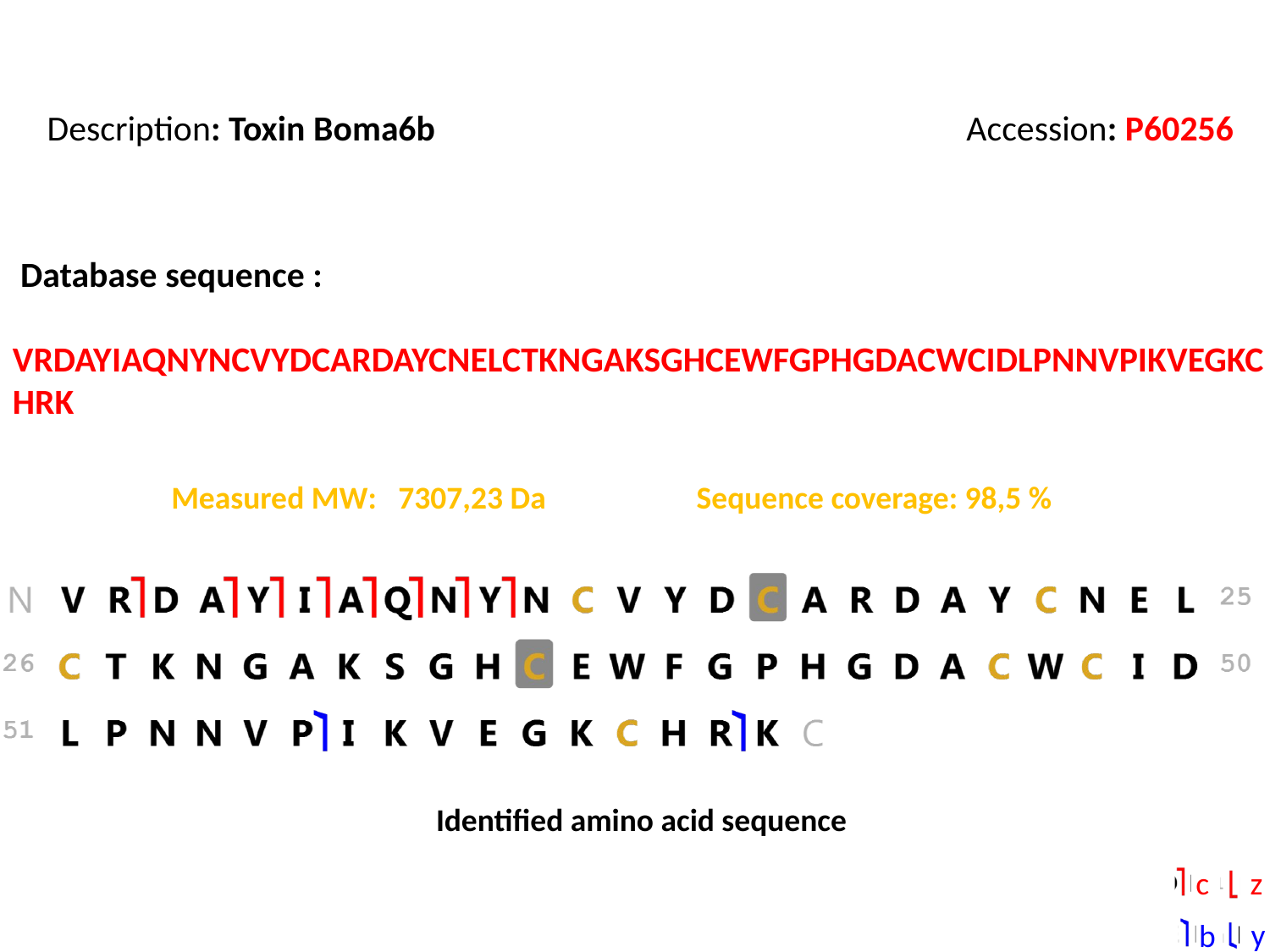

Description: Toxin Boma6b Accession: P60256
 Database sequence :
VRDAYIAQNYNCVYDCARDAYCNELCTKNGAKSGHCEWFGPHGDACWCIDLPNNVPIKVEGKCHRK
Measured MW: 7307,23 Da Sequence coverage: 98,5 %
Identified amino acid sequence
c
z
y
b

## Slide 10
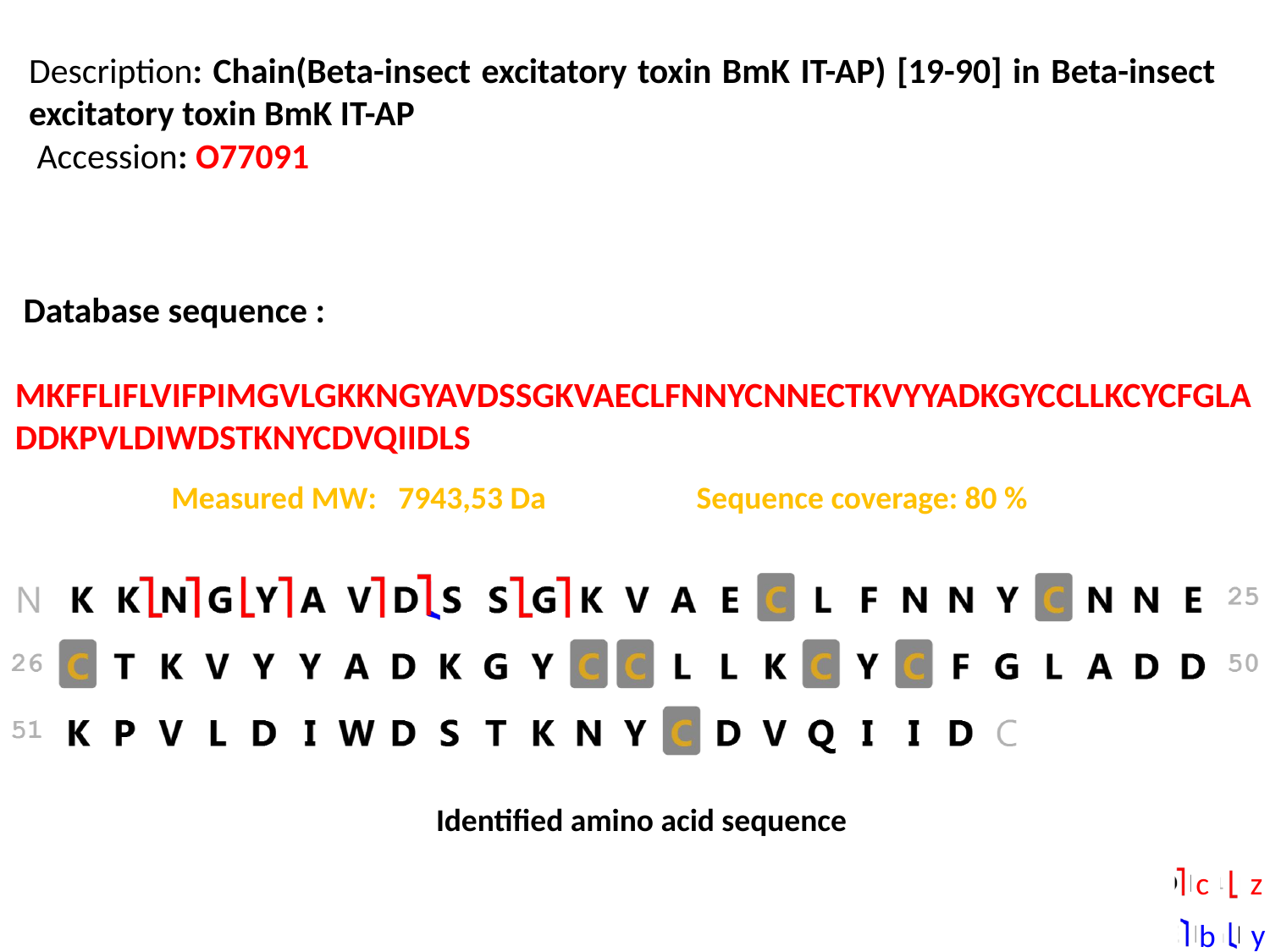

Description: Chain(Beta-insect excitatory toxin BmK IT-AP) [19-90] in Beta-insect excitatory toxin BmK IT-AP
 Accession: O77091
 Database sequence :
MKFFLIFLVIFPIMGVLGKKNGYAVDSSGKVAECLFNNYCNNECTKVYYADKGYCCLLKCYCFGLADDKPVLDIWDSTKNYCDVQIIDLS
Measured MW: 7943,53 Da Sequence coverage: 80 %
Identified amino acid sequence
c
z
y
b

## Slide 11
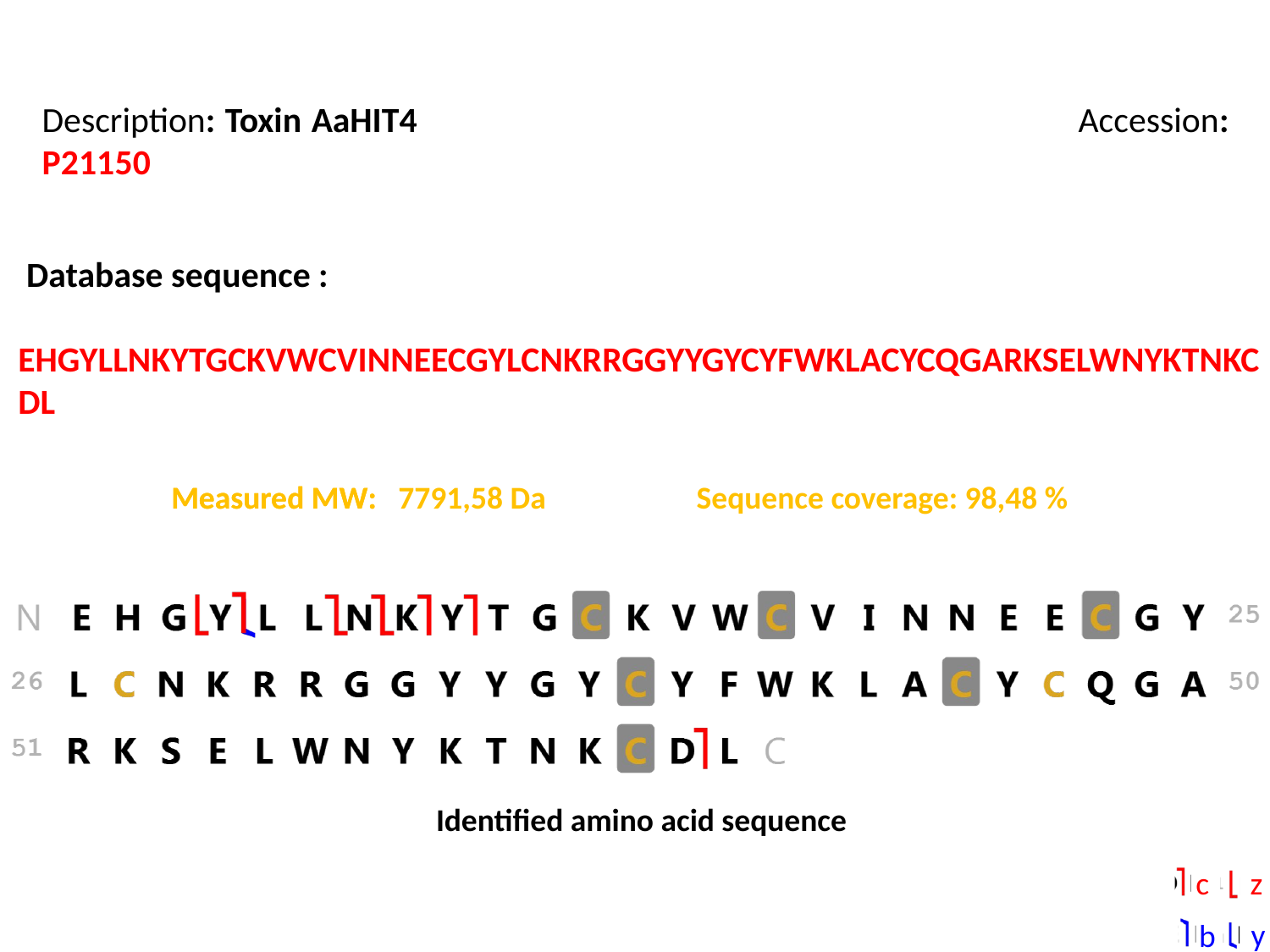

Description: Toxin AaHIT4	 Accession: P21150
 Database sequence :
EHGYLLNKYTGCKVWCVINNEECGYLCNKRRGGYYGYCYFWKLACYCQGARKSELWNYKTNKCDL
Measured MW:
Measured MW: 7791,58 Da Sequence coverage: 98,48 %
Identified amino acid sequence
c
z
y
b

## Slide 12
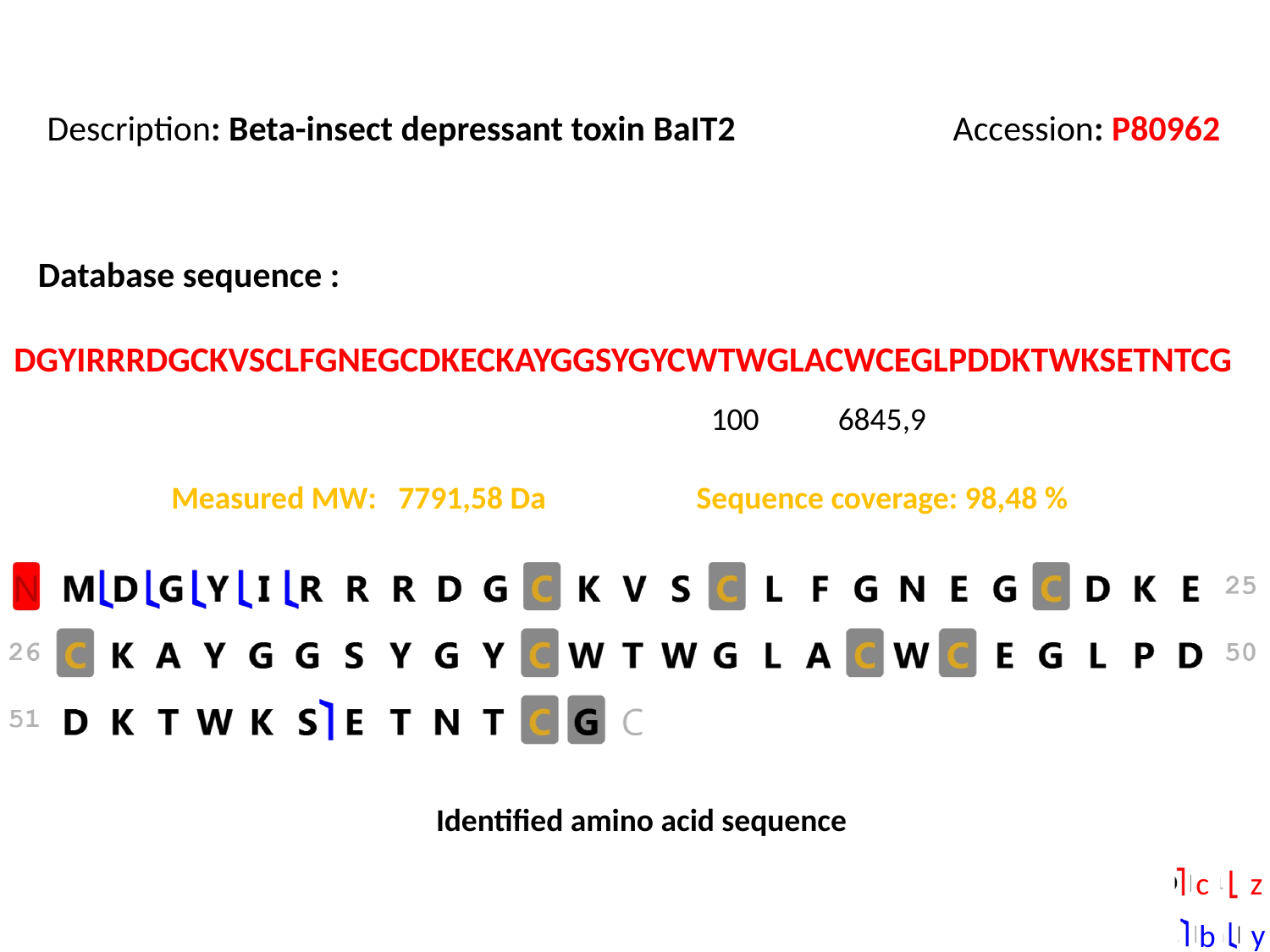

Description: Beta-insect depressant toxin BaIT2 Accession: P80962
 Database sequence :
DGYIRRRDGCKVSCLFGNEGCDKECKAYGGSYGYCWTWGLACWCEGLPDDKTWKSETNTCG
			100	6845,9
Measured MW: 7791,58 Da Sequence coverage: 98,48 %
Identified amino acid sequence
c
z
y
b

## Slide 13
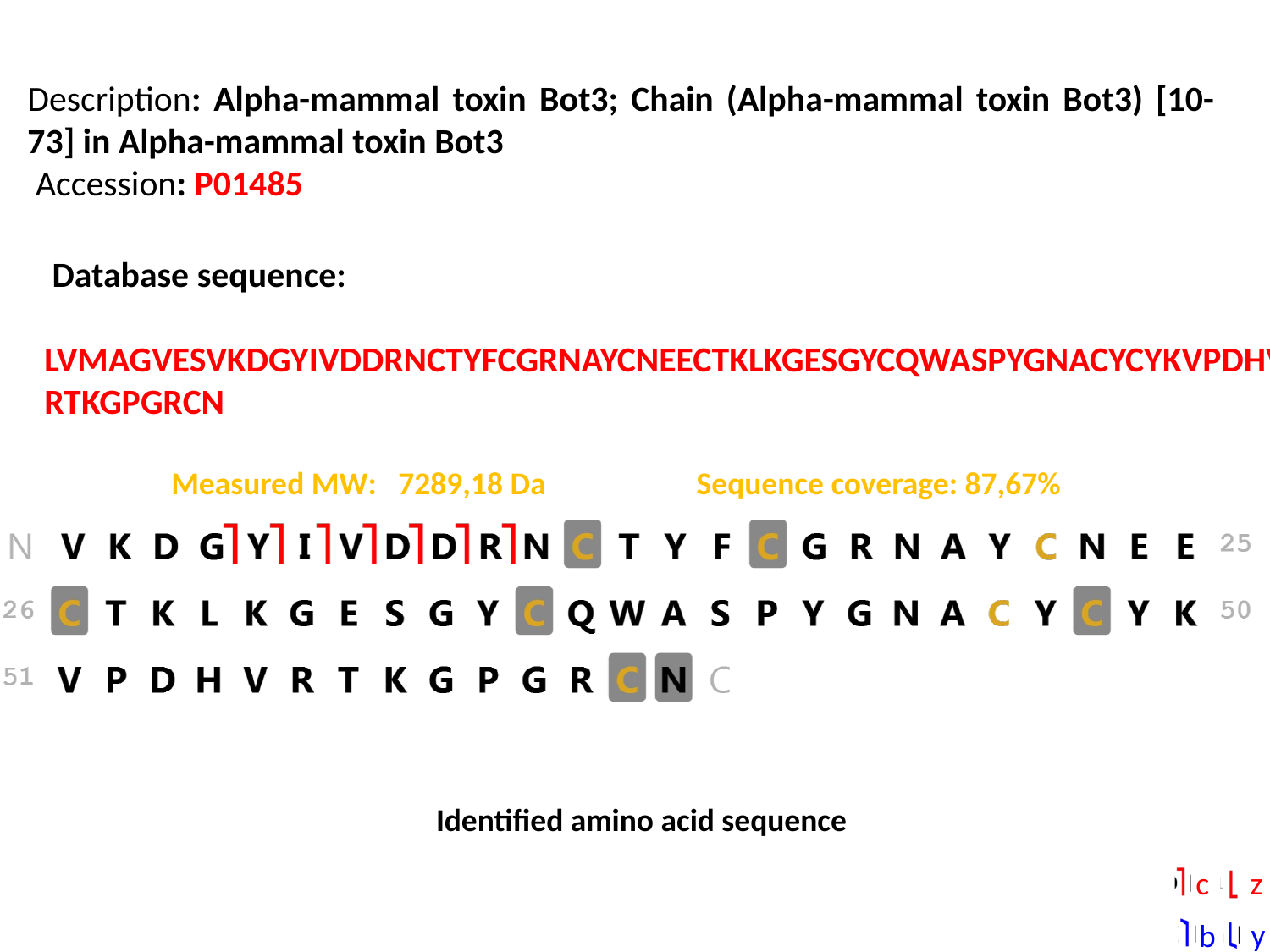

Description: Alpha-mammal toxin Bot3; Chain (Alpha-mammal toxin Bot3) [10-73] in Alpha-mammal toxin Bot3
 Accession: P01485
 Database sequence:
LVMAGVESVKDGYIVDDRNCTYFCGRNAYCNEECTKLKGESGYCQWASPYGNACYCYKVPDHVRTKGPGRCN
Measured MW: 7289,18 Da Sequence coverage: 87,67%
Identified amino acid sequence
c
z
y
b

## Slide 14
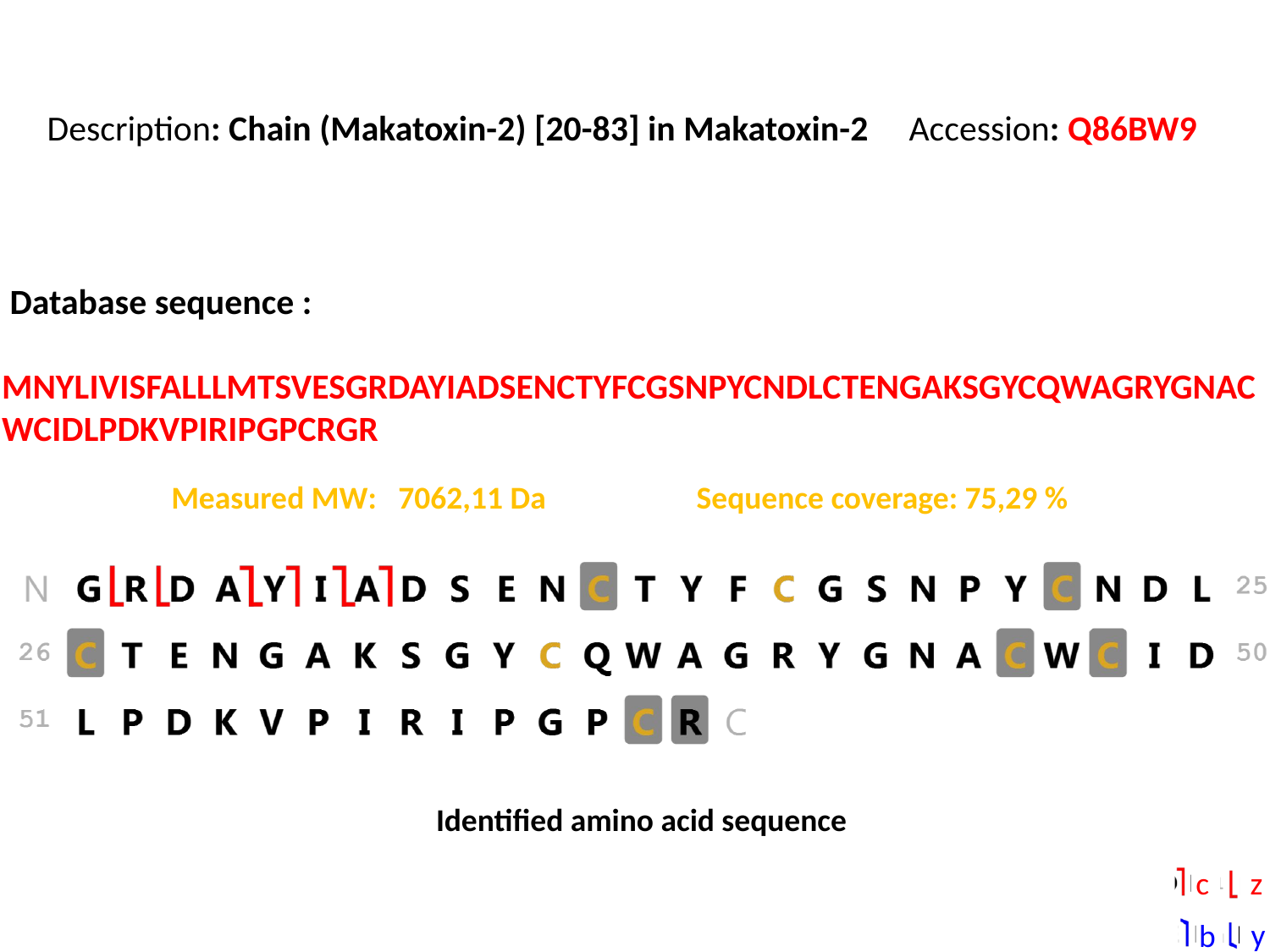

Description: Chain (Makatoxin-2) [20-83] in Makatoxin-2 Accession: Q86BW9
 Database sequence :
MNYLIVISFALLLMTSVESGRDAYIADSENCTYFCGSNPYCNDLCTENGAKSGYCQWAGRYGNACWCIDLPDKVPIRIPGPCRGR
Measured MW: 7062,11 Da Sequence coverage: 75,29 %
Identified amino acid sequence
c
z
y
b

## Slide 15
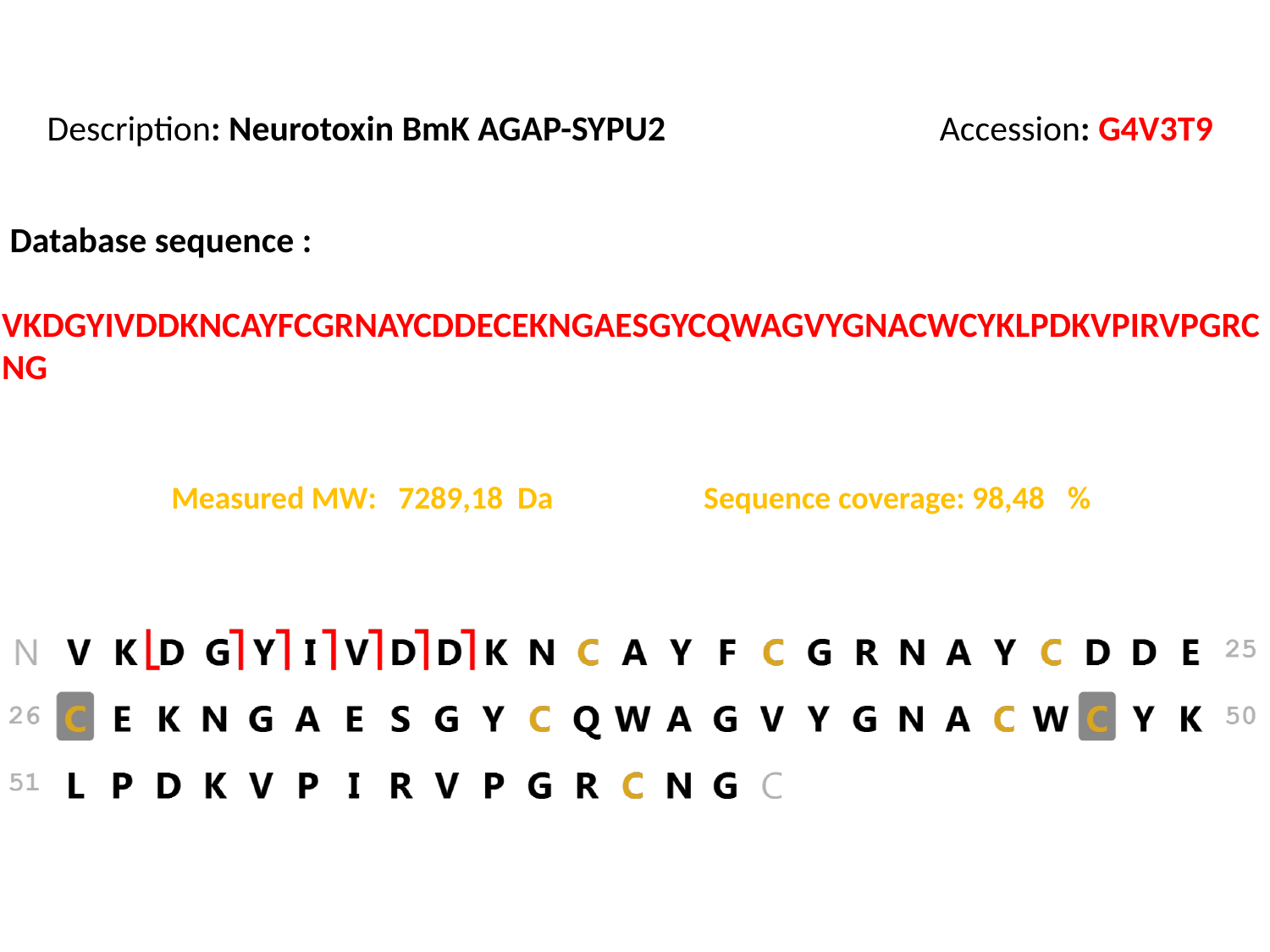

Description: Neurotoxin BmK AGAP-SYPU2 Accession: G4V3T9
 Database sequence :
VKDGYIVDDKNCAYFCGRNAYCDDECEKNGAESGYCQWAGVYGNACWCYKLPDKVPIRVPGRCNG
Measured MW: 7289,18 Da Sequence coverage: 98,48	 %

## Slide 16
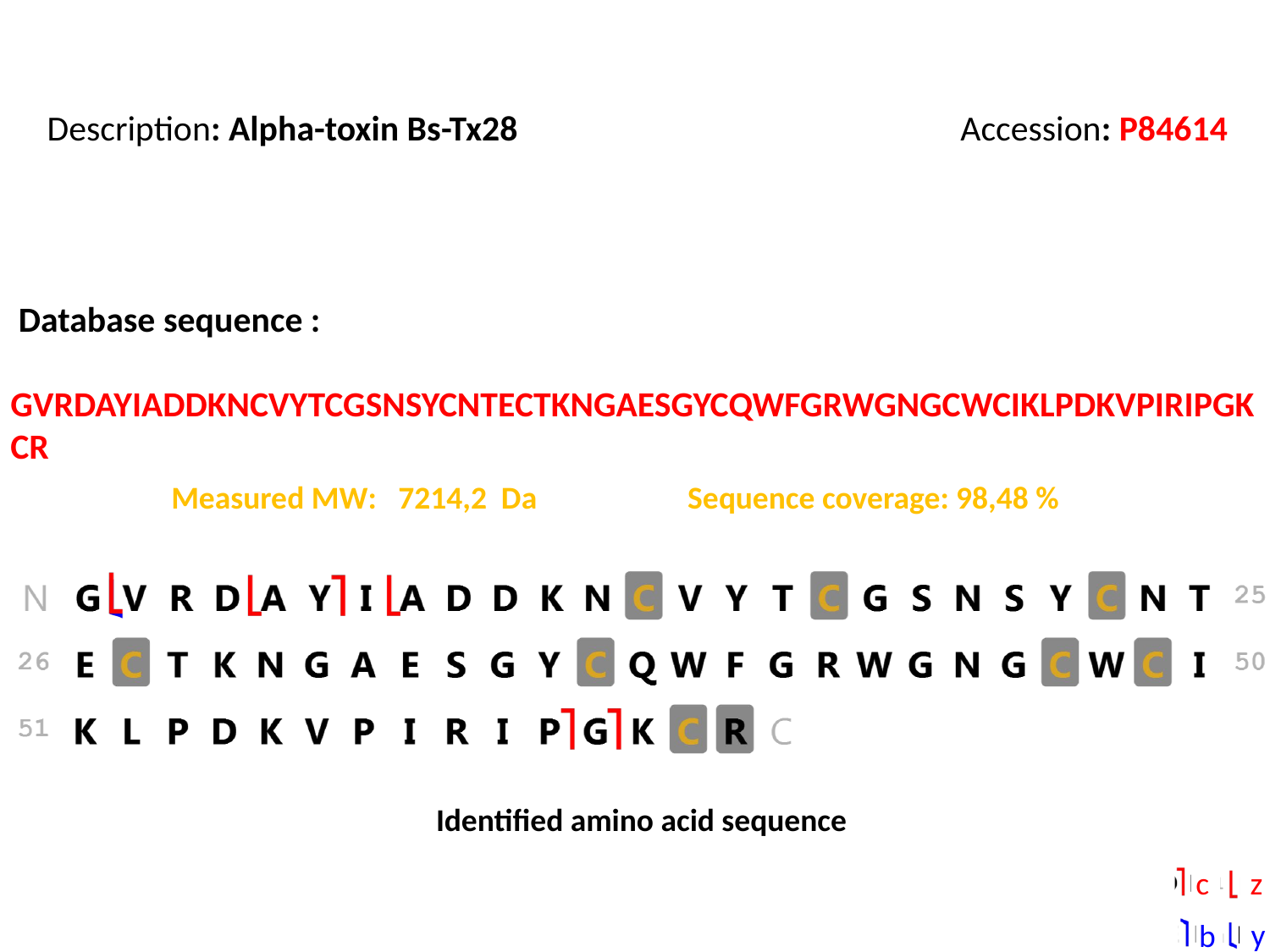

Description: Alpha-toxin Bs-Tx28 Accession: P84614
 Database sequence :
GVRDAYIADDKNCVYTCGSNSYCNTECTKNGAESGYCQWFGRWGNGCWCIKLPDKVPIRIPGKCR
Measured MW: 7214,2 Da Sequence coverage: 98,48 %
Identified amino acid sequence
c
z
y
b

## Slide 17
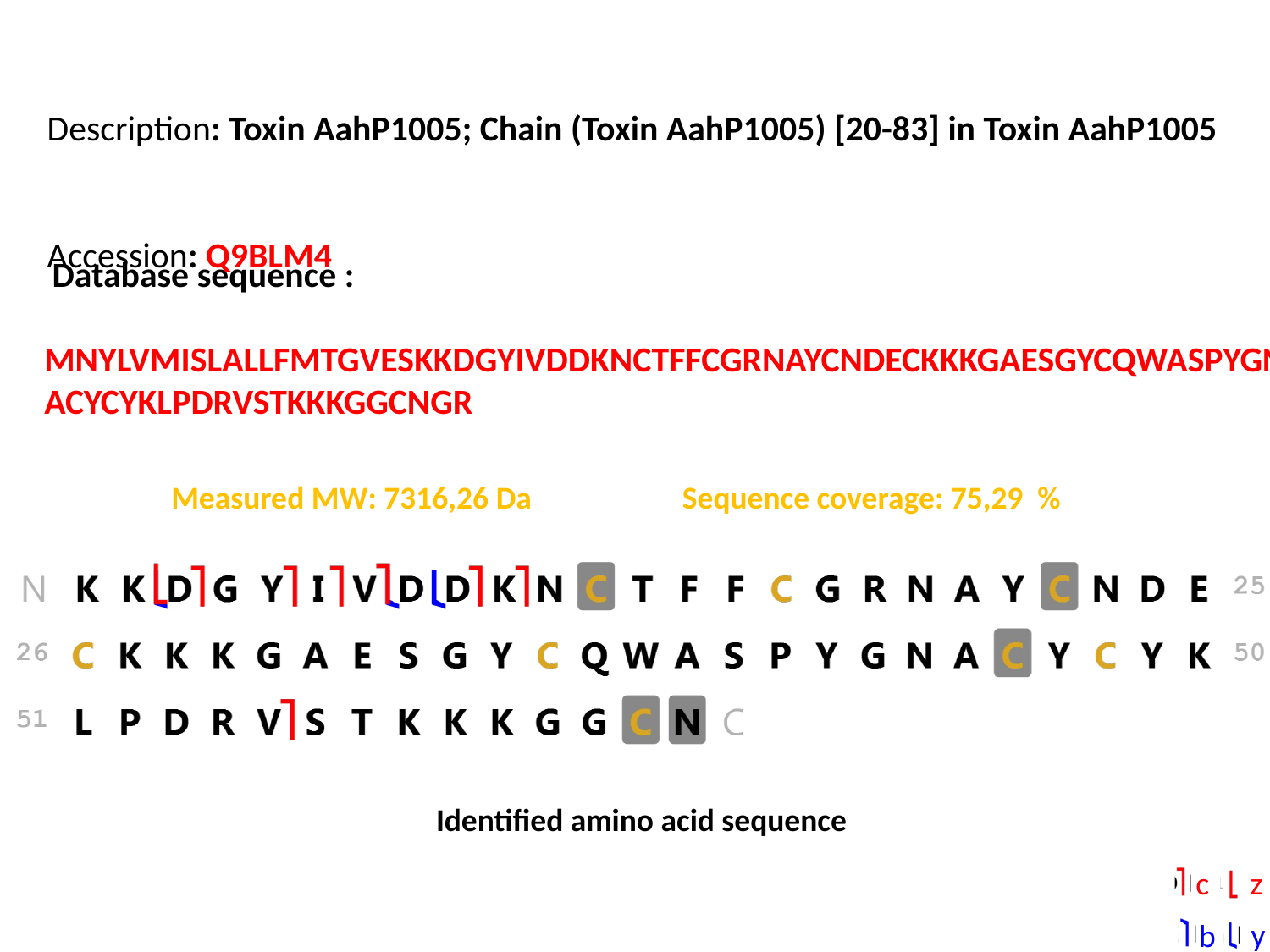

Description: Toxin AahP1005; Chain (Toxin AahP1005) [20-83] in Toxin AahP1005
Accession: Q9BLM4
 Database sequence :
MNYLVMISLALLFMTGVESKKDGYIVDDKNCTFFCGRNAYCNDECKKKGAESGYCQWASPYGNACYCYKLPDRVSTKKKGGCNGR
Measured MW: 7316,26 Da Sequence coverage: 75,29 %
Identified amino acid sequence
c
z
y
b

## Slide 18
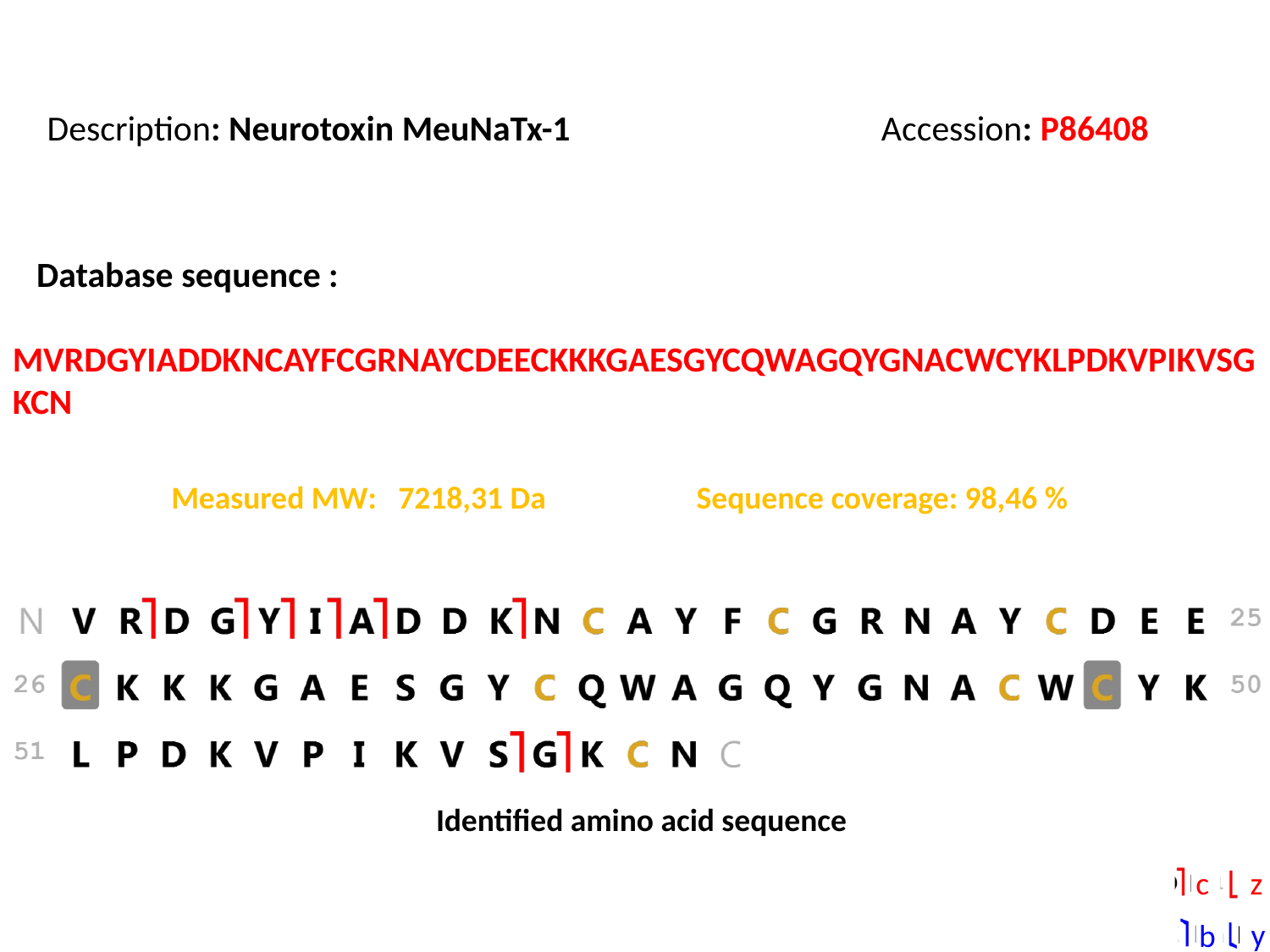

Description: Neurotoxin MeuNaTx-1		 Accession: P86408
 Database sequence :
MVRDGYIADDKNCAYFCGRNAYCDEECKKKGAESGYCQWAGQYGNACWCYKLPDKVPIKVSGKCN
Measured MW: 7218,31 Da Sequence coverage: 98,46 %
Identified amino acid sequence
c
z
y
b

## Slide 19
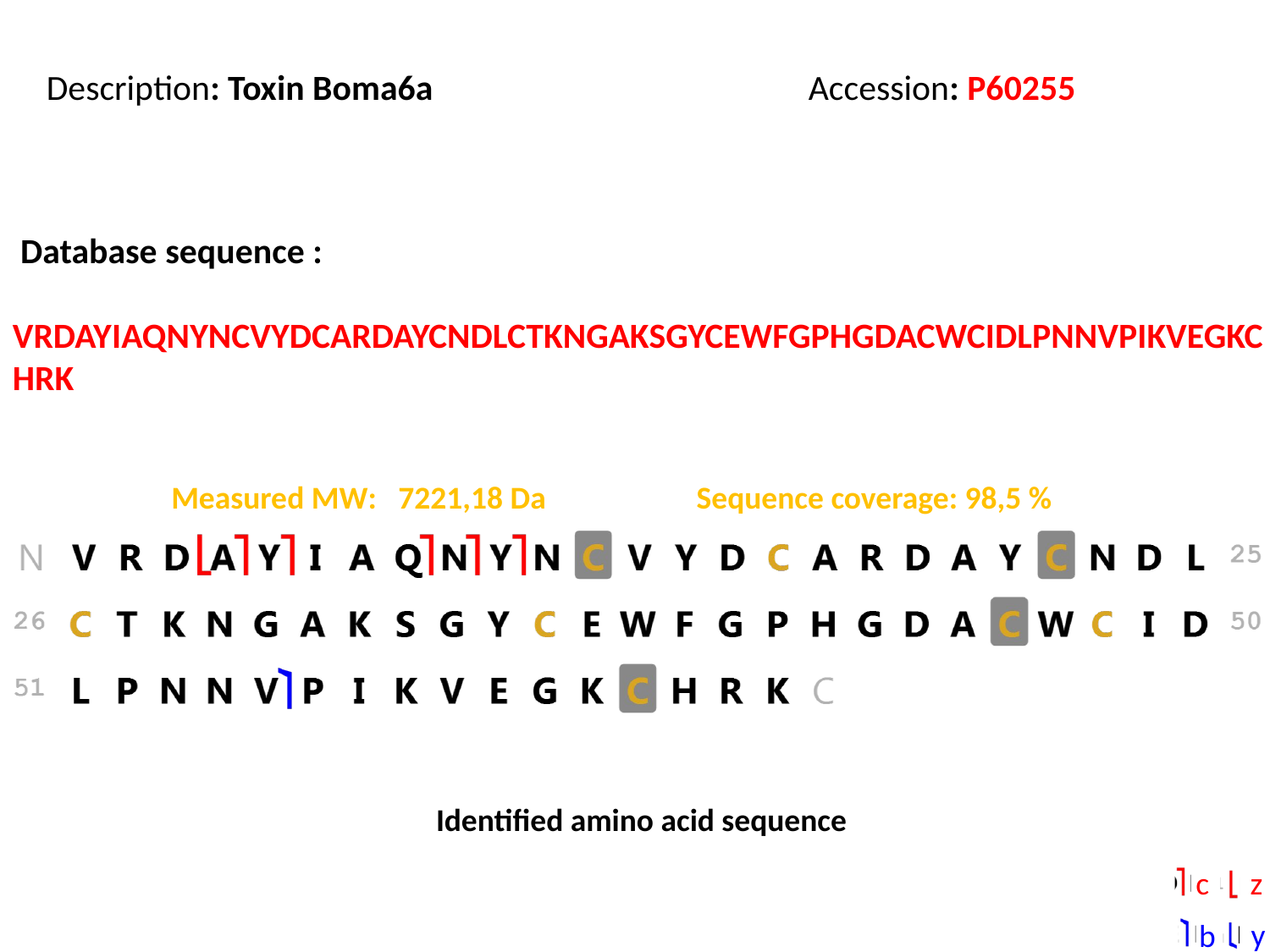

Description: Toxin Boma6a			Accession: P60255
 Database sequence :
VRDAYIAQNYNCVYDCARDAYCNDLCTKNGAKSGYCEWFGPHGDACWCIDLPNNVPIKVEGKCHRK
Measured MW: 7221,18 Da Sequence coverage: 98,5 %
Identified amino acid sequence
c
z
y
b

## Slide 20
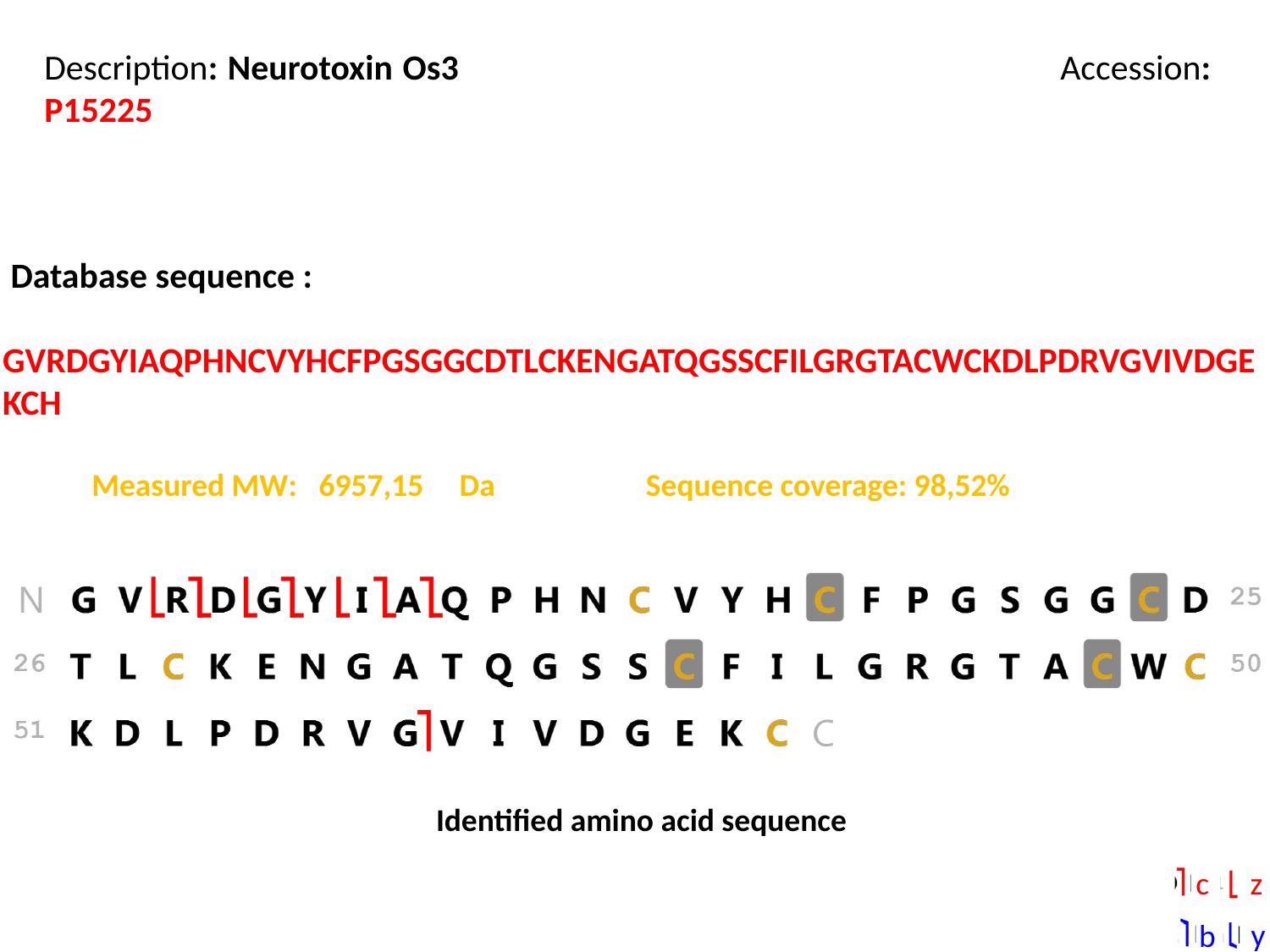

Description: Neurotoxin Os3 Accession: P15225
 Database sequence :
GVRDGYIAQPHNCVYHCFPGSGGCDTLCKENGATQGSSCFILGRGTACWCKDLPDRVGVIVDGEKCH
Measured MW: 6957,15 Da Sequence coverage: 98,52%
Identified amino acid sequence
c
z
y
b

## Slide 21
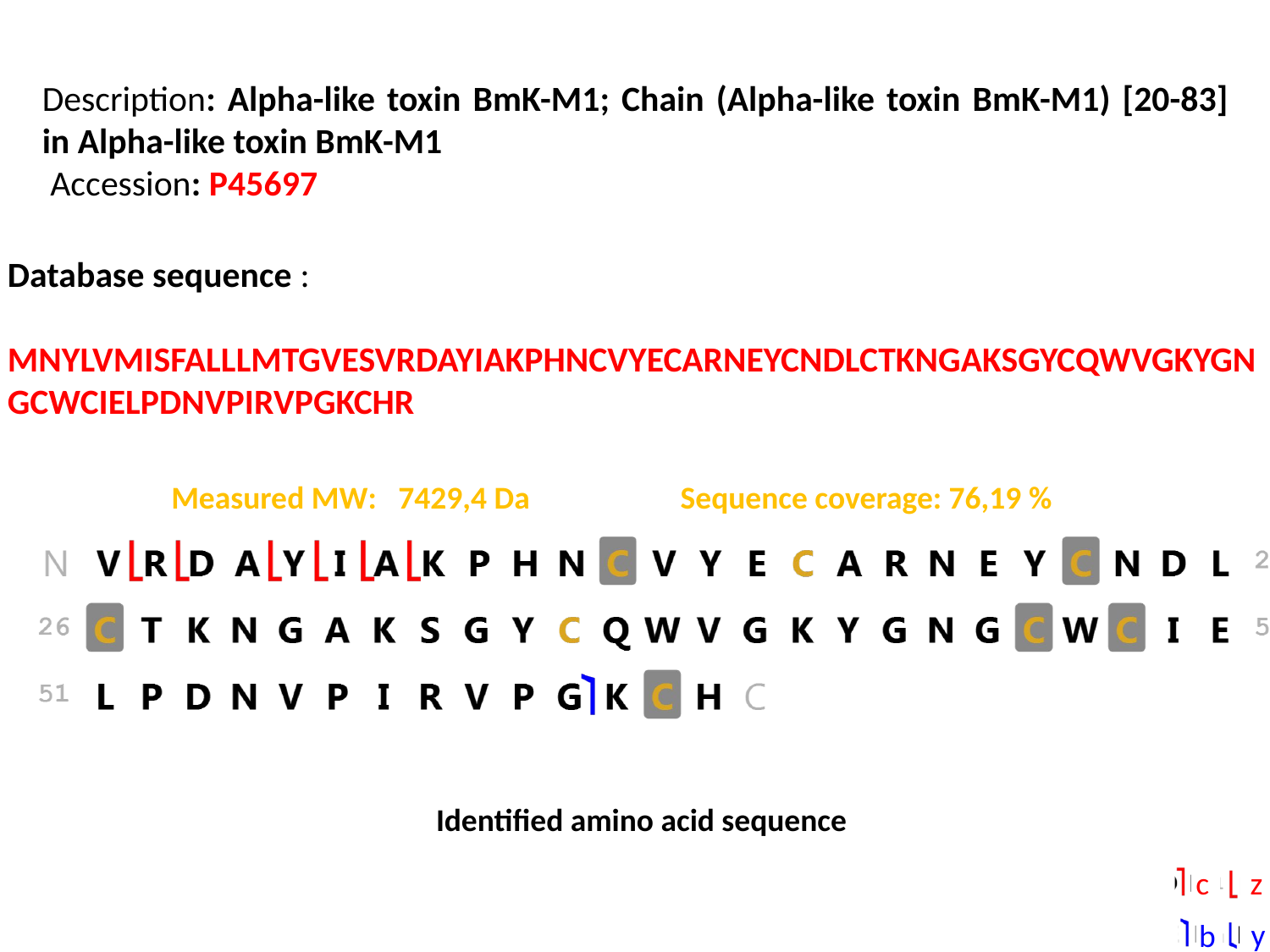

Description: Alpha-like toxin BmK-M1; Chain (Alpha-like toxin BmK-M1) [20-83] in Alpha-like toxin BmK-M1
 Accession: P45697
Database sequence :
MNYLVMISFALLLMTGVESVRDAYIAKPHNCVYECARNEYCNDLCTKNGAKSGYCQWVGKYGNGCWCIELPDNVPIRVPGKCHR
Measured MW: 7429,4 Da Sequence coverage: 76,19 %
Identified amino acid sequence
c
z
y
b

## Slide 22
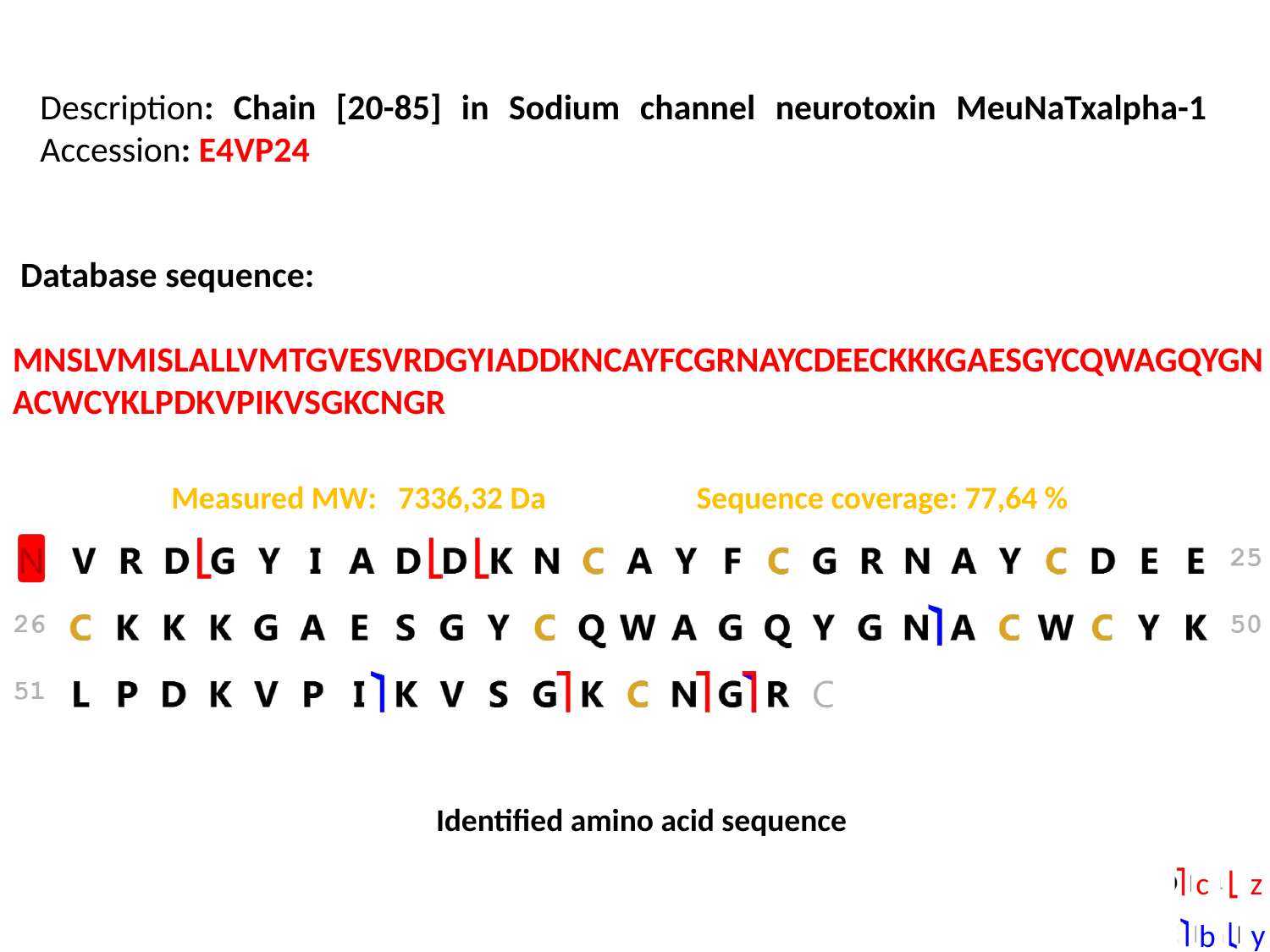

Description: Chain [20-85] in Sodium channel neurotoxin MeuNaTxalpha-1 Accession: E4VP24
 Database sequence:
MNSLVMISLALLVMTGVESVRDGYIADDKNCAYFCGRNAYCDEECKKKGAESGYCQWAGQYGNACWCYKLPDKVPIKVSGKCNGR
Measured MW: 7336,32 Da Sequence coverage: 77,64 %
Identified amino acid sequence
c
z
y
b

## Slide 23
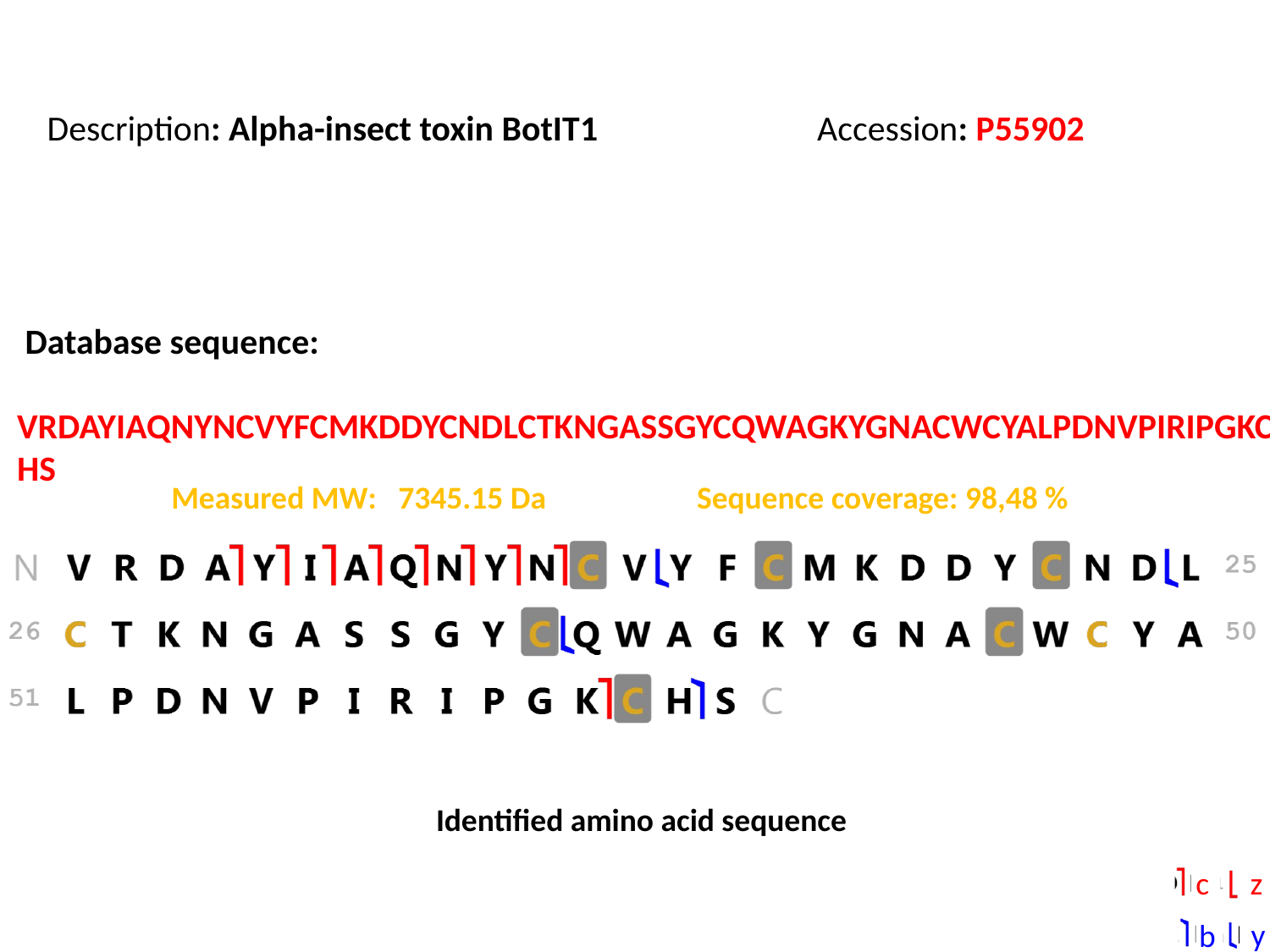

Description: Alpha-insect toxin BotIT1		 Accession: P55902
 Database sequence:
VRDAYIAQNYNCVYFCMKDDYCNDLCTKNGASSGYCQWAGKYGNACWCYALPDNVPIRIPGKCHS
Measured MW: 7345.15 Da Sequence coverage: 98,48 %
Identified amino acid sequence
c
z
y
b

## Slide 24
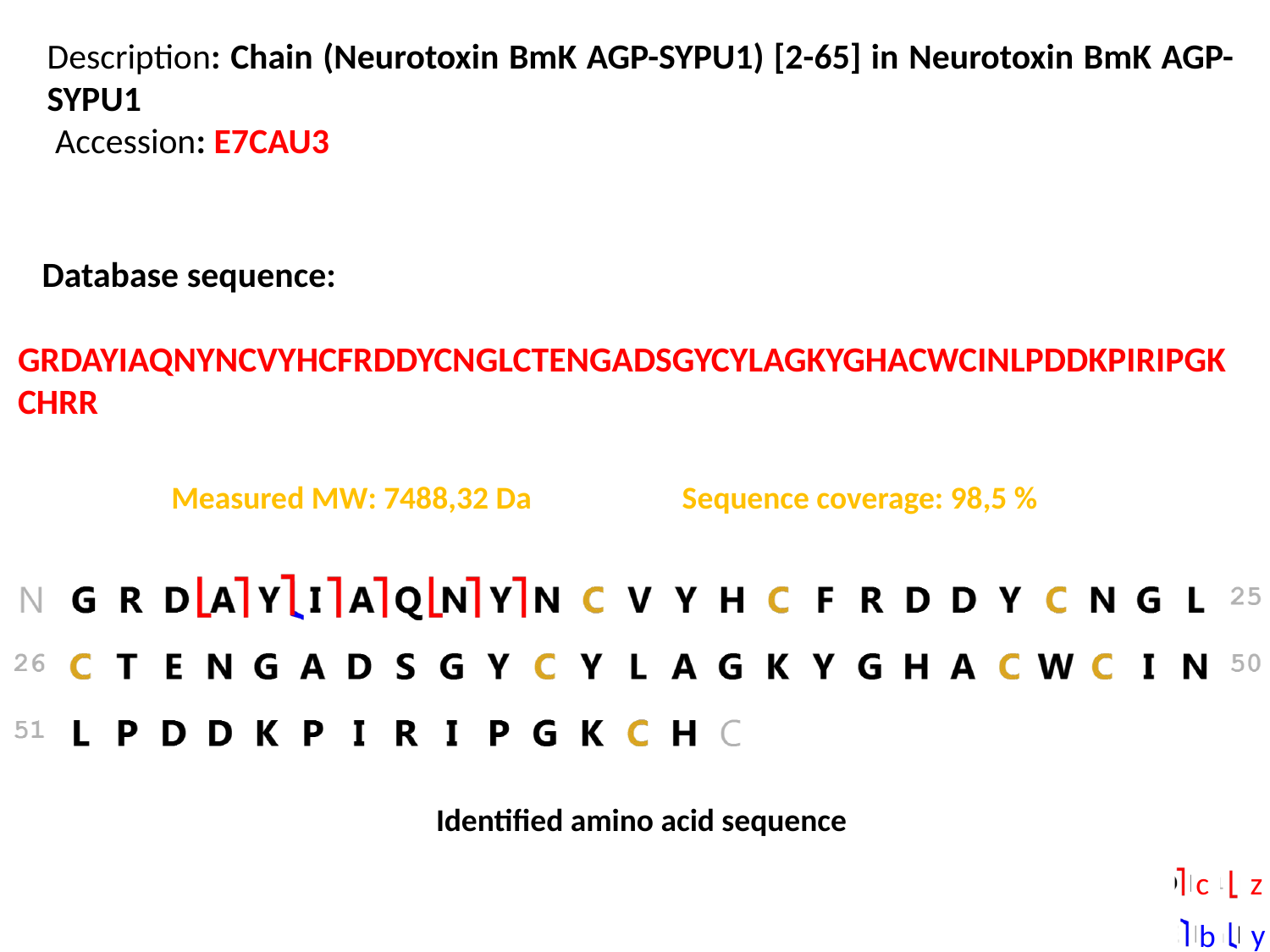

Description: Chain (Neurotoxin BmK AGP-SYPU1) [2-65] in Neurotoxin BmK AGP-SYPU1
 Accession: E7CAU3
 Database sequence:
GRDAYIAQNYNCVYHCFRDDYCNGLCTENGADSGYCYLAGKYGHACWCINLPDDKPIRIPGK
CHRR
Measured MW: 7488,32 Da Sequence coverage: 98,5 %
Identified amino acid sequence
c
z
y
b

## Slide 25
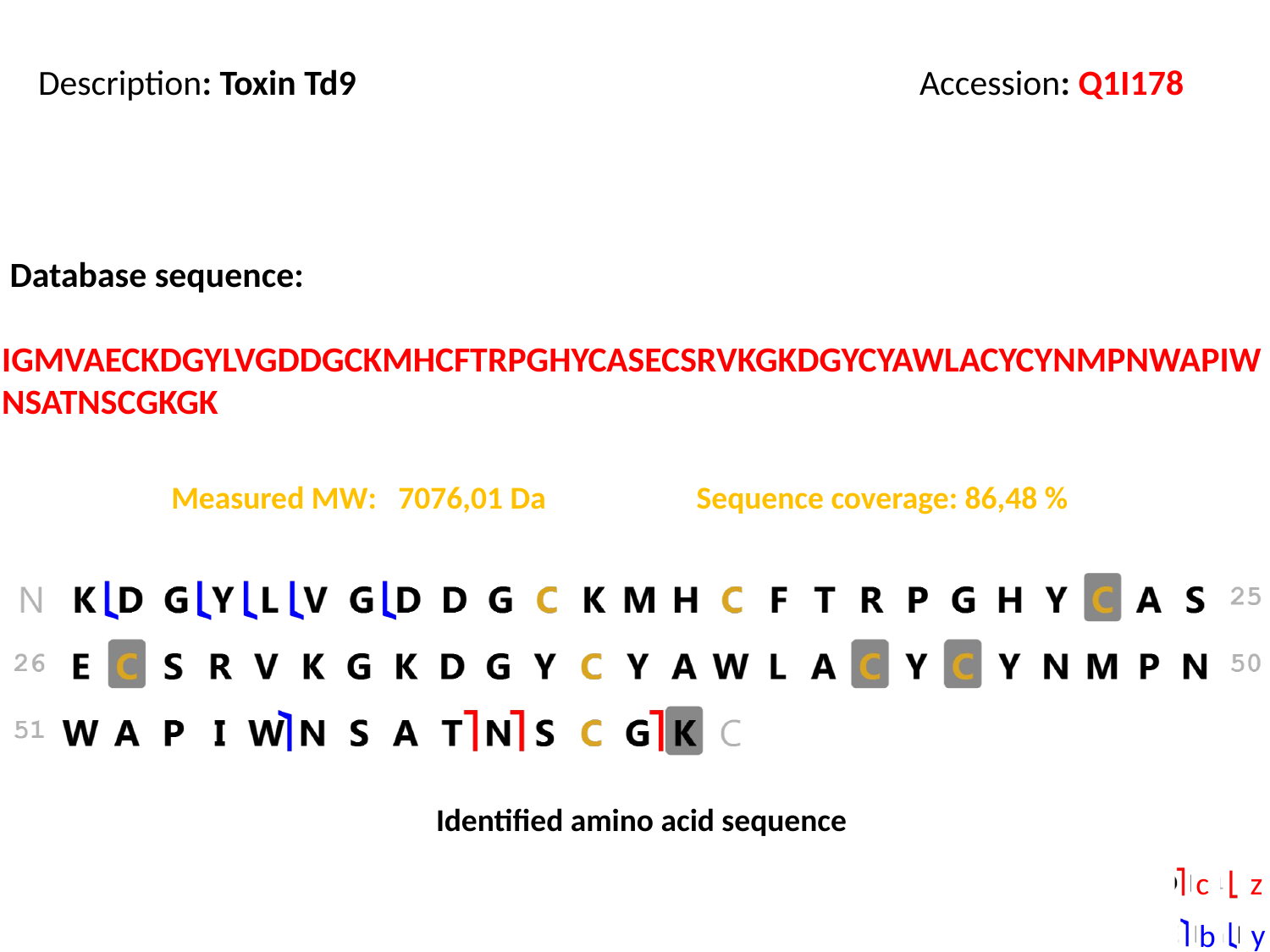

Description: Toxin Td9 Accession: Q1I178
 Database sequence:
IGMVAECKDGYLVGDDGCKMHCFTRPGHYCASECSRVKGKDGYCYAWLACYCYNMPNWAPIWNSATNSCGKGK
Measured MW: 7076,01 Da Sequence coverage: 86,48 %
Identified amino acid sequence
c
z
y
b

## Slide 26
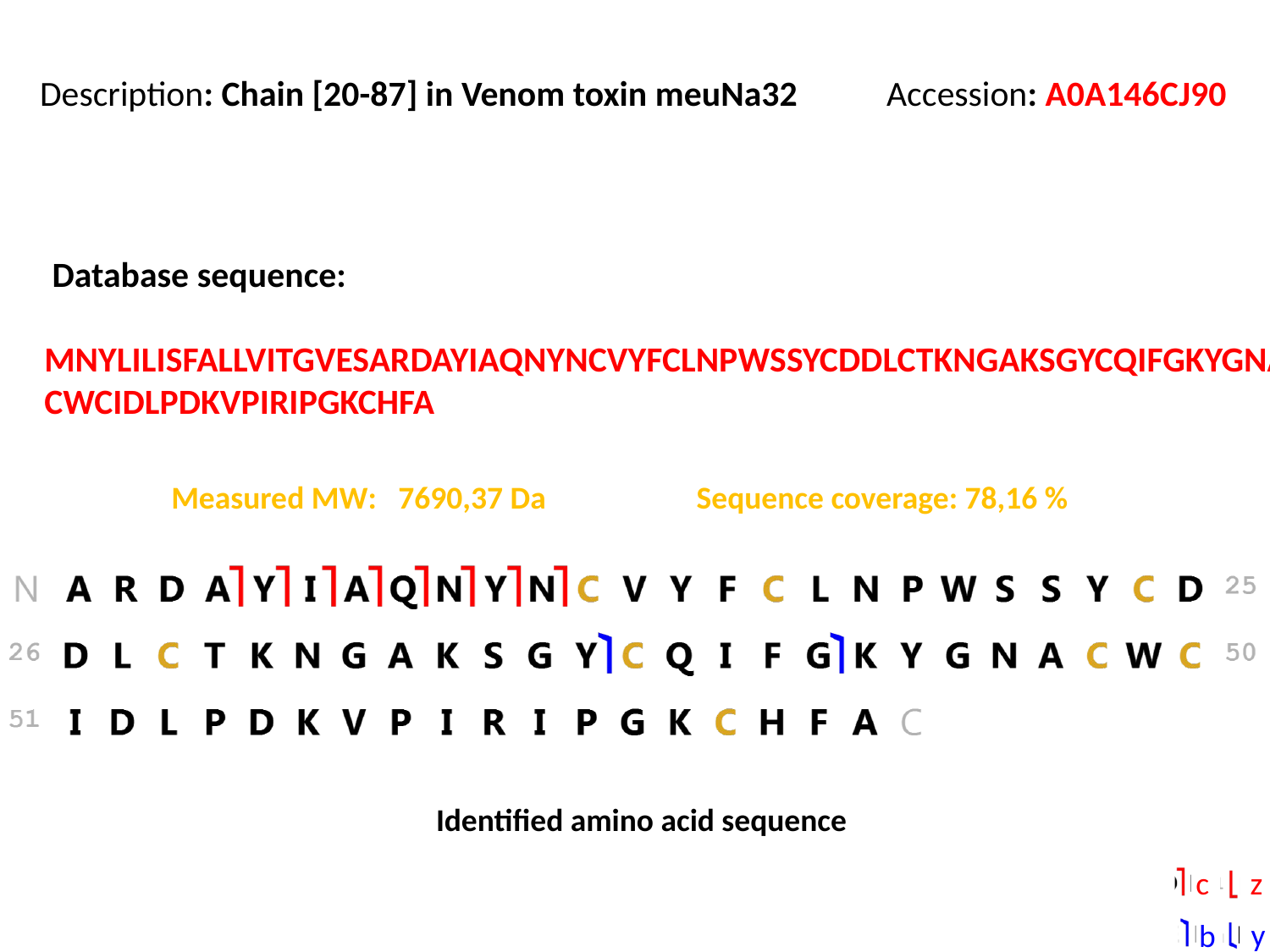

Description: Chain [20-87] in Venom toxin meuNa32 Accession: A0A146CJ90
 Database sequence:
MNYLILISFALLVITGVESARDAYIAQNYNCVYFCLNPWSSYCDDLCTKNGAKSGYCQIFGKYGNACWCIDLPDKVPIRIPGKCHFA
Measured MW: 7690,37 Da Sequence coverage: 78,16 %
Identified amino acid sequence
c
z
y
b

## Slide 27
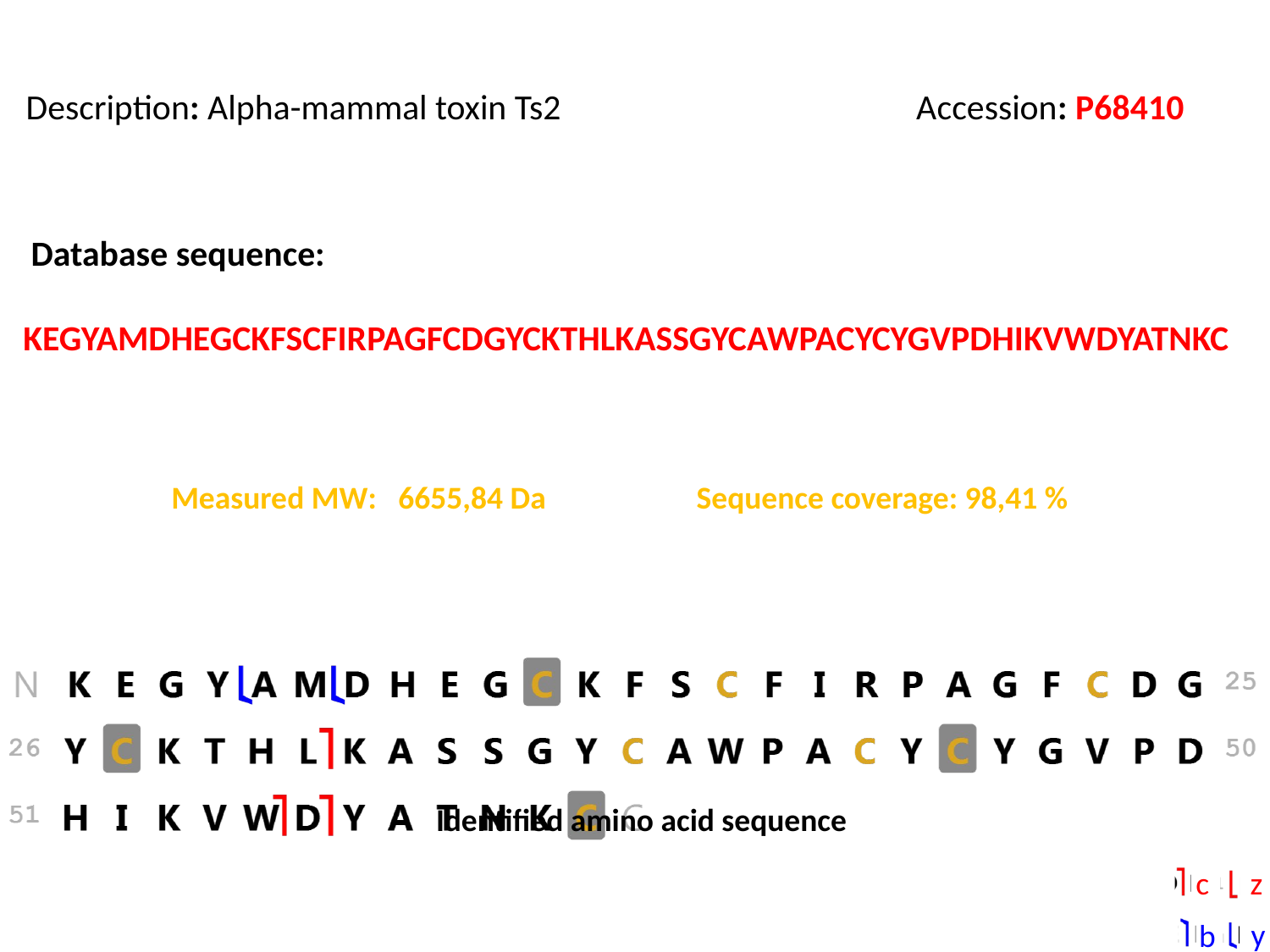

Description: Alpha-mammal toxin Ts2		 Accession: P68410
 Database sequence:
KEGYAMDHEGCKFSCFIRPAGFCDGYCKTHLKASSGYCAWPACYCYGVPDHIKVWDYATNKC
Measured MW: 6655,84 Da Sequence coverage: 98,41 %
Identified amino acid sequence
c
z
y
b

## Slide 28
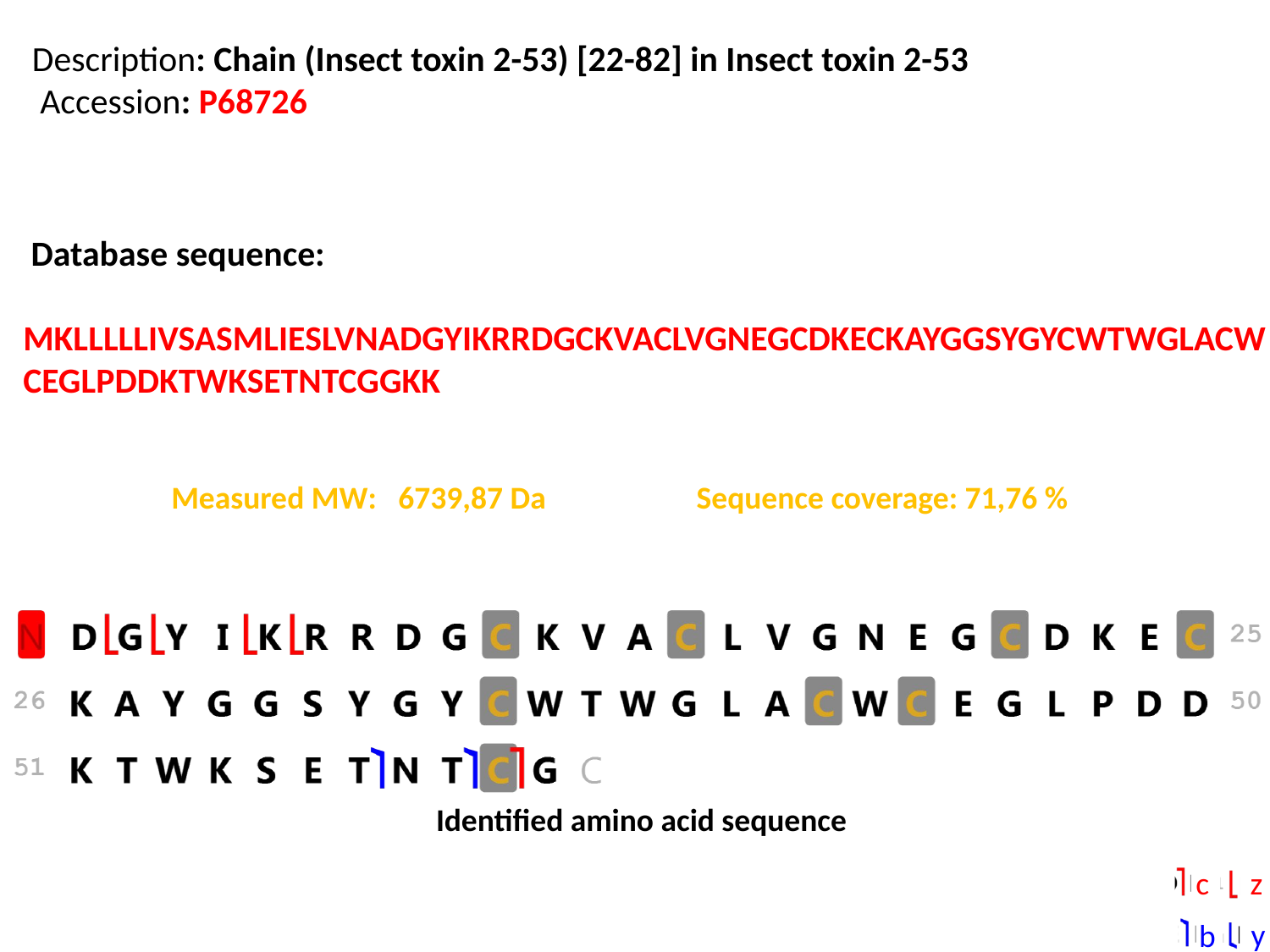

Description: Chain (Insect toxin 2-53) [22-82] in Insect toxin 2-53
 Accession: P68726
 Database sequence:
MKLLLLLIVSASMLIESLVNADGYIKRRDGCKVACLVGNEGCDKECKAYGGSYGYCWTWGLACWCEGLPDDKTWKSETNTCGGKK
Measured MW: 6739,87 Da Sequence coverage: 71,76 %
Identified amino acid sequence
c
z
y
b

## Slide 29
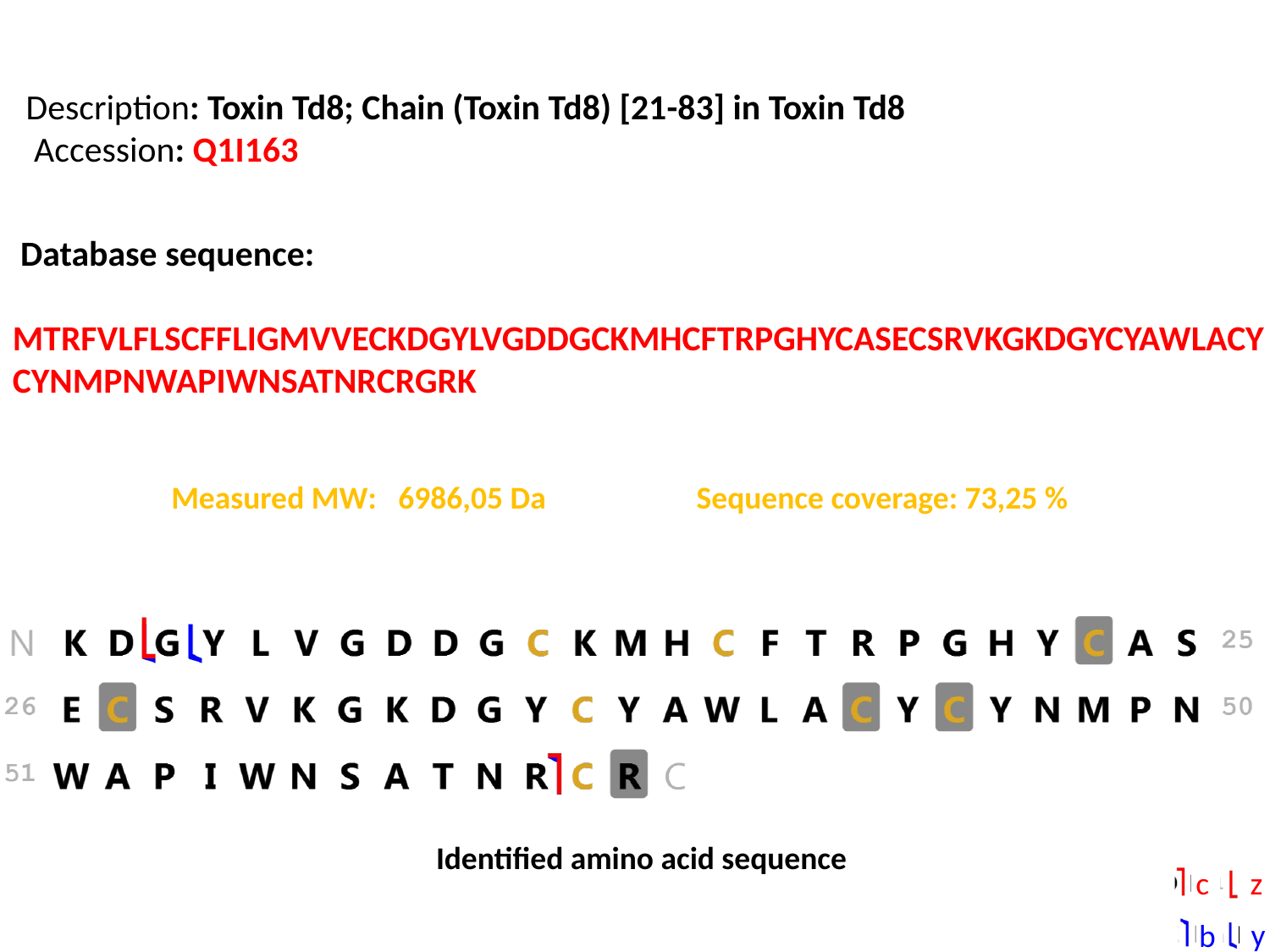

Description: Toxin Td8; Chain (Toxin Td8) [21-83] in Toxin Td8
 Accession: Q1I163
 Database sequence:
MTRFVLFLSCFFLIGMVVECKDGYLVGDDGCKMHCFTRPGHYCASECSRVKGKDGYCYAWLACYCYNMPNWAPIWNSATNRCRGRK
Measured MW: 6986,05 Da Sequence coverage: 73,25 %
Identified amino acid sequence
c
z
y
b

## Slide 30
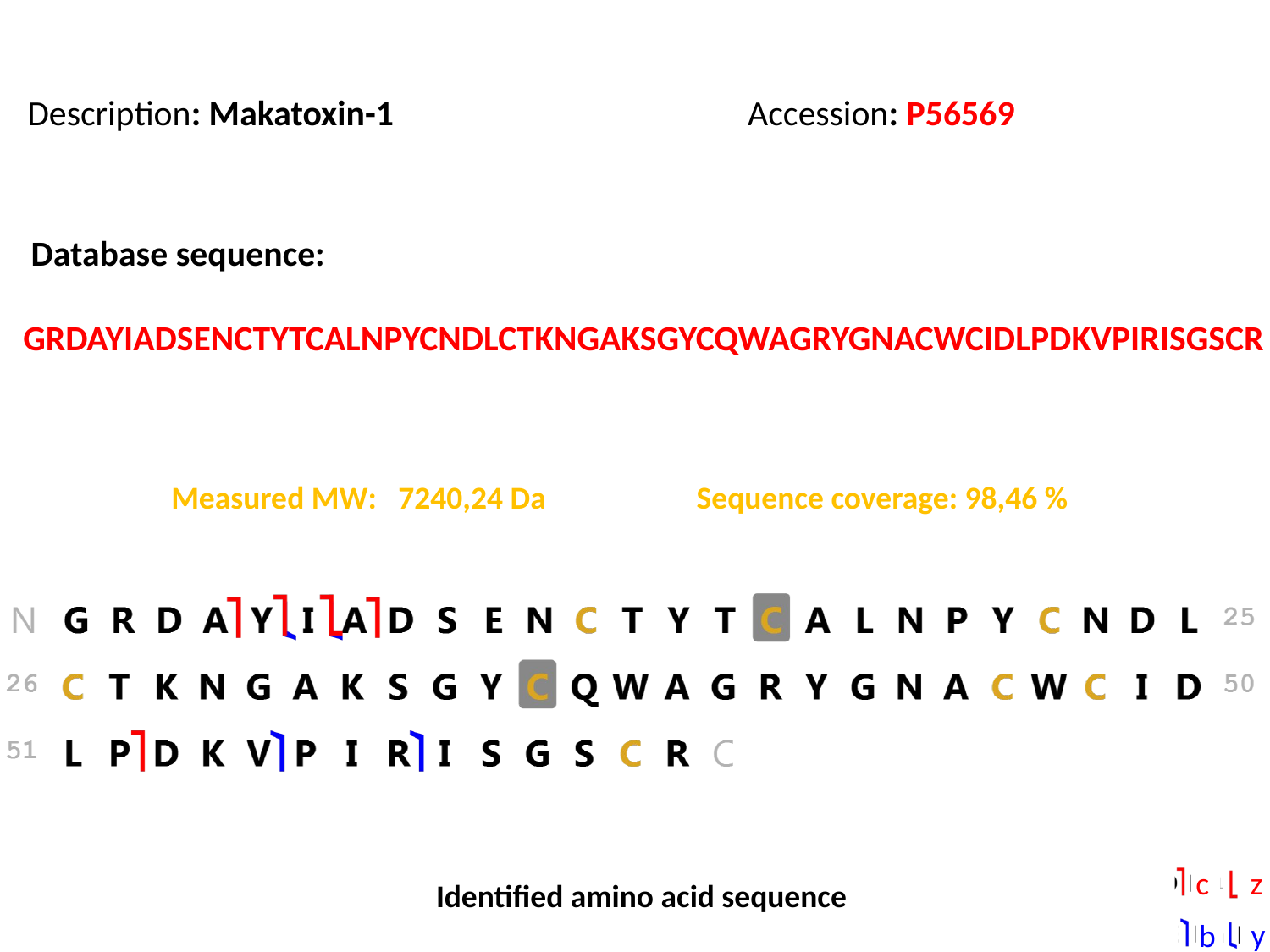

Description: Makatoxin-1 Accession: P56569
 Database sequence:
GRDAYIADSENCTYTCALNPYCNDLCTKNGAKSGYCQWAGRYGNACWCIDLPDKVPIRISGSCR
Measured MW: 7240,24 Da Sequence coverage: 98,46 %
Identified amino acid sequence
c
z
y
b

## Slide 31
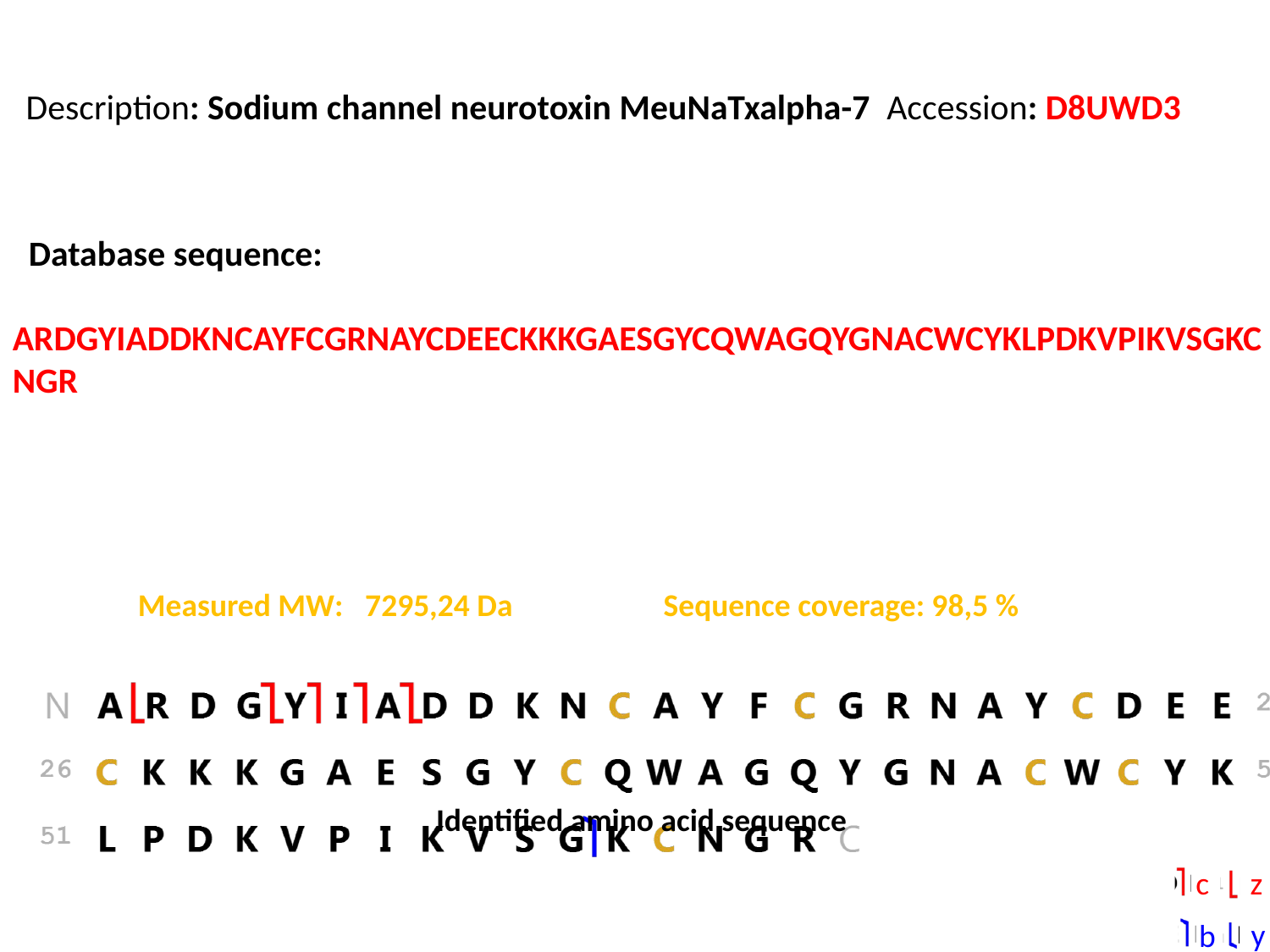

Description: Sodium channel neurotoxin MeuNaTxalpha-7 Accession: D8UWD3
 Database sequence:
ARDGYIADDKNCAYFCGRNAYCDEECKKKGAESGYCQWAGQYGNACWCYKLPDKVPIKVSGKCNGR
Measured MW: 7295,24 Da Sequence coverage: 98,5 %
Identified amino acid sequence
c
z
y
b

## Slide 32
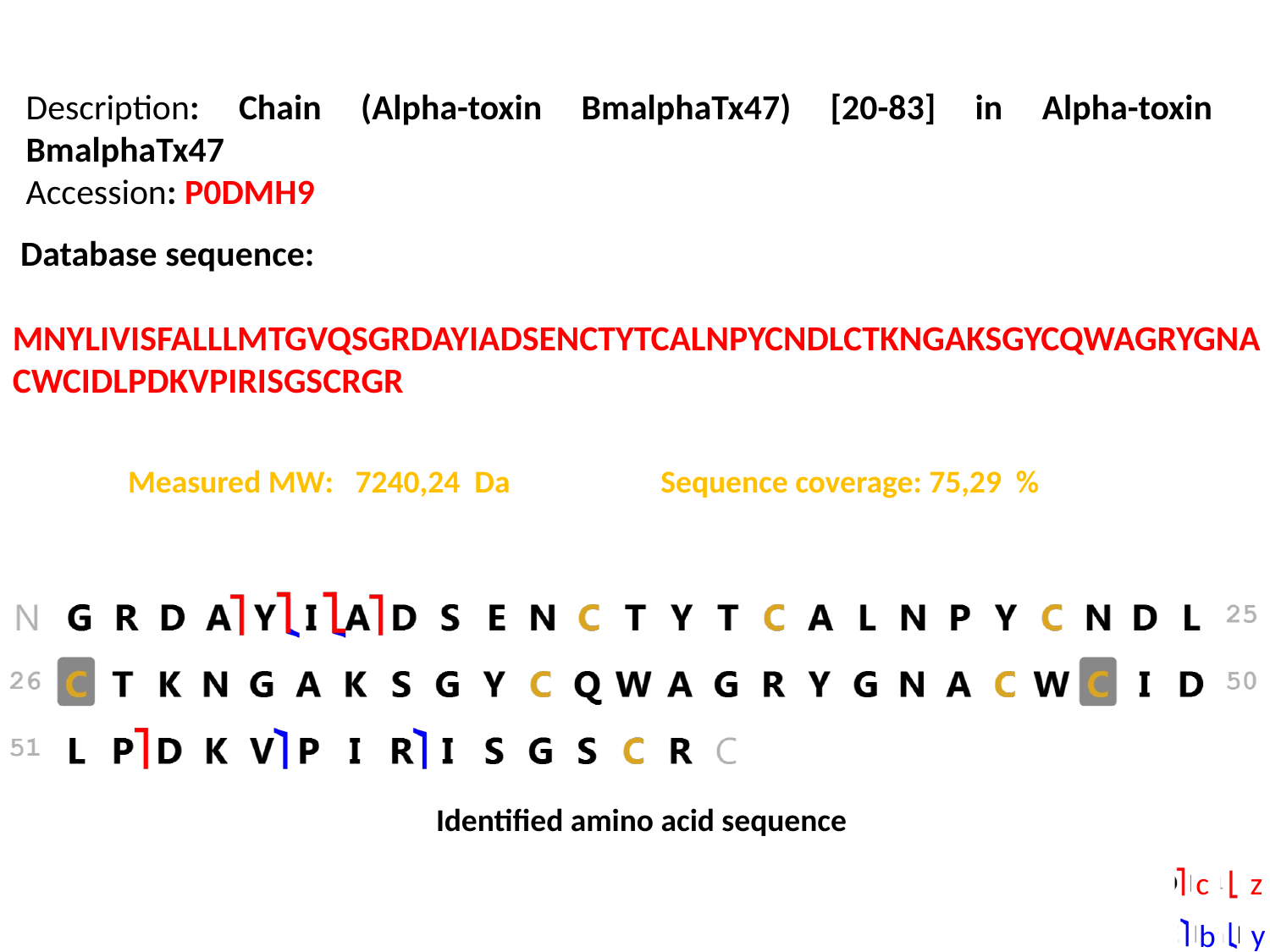

Description: Chain (Alpha-toxin BmalphaTx47) [20-83] in Alpha-toxin BmalphaTx47
Accession: P0DMH9
 Database sequence:
MNYLIVISFALLLMTGVQSGRDAYIADSENCTYTCALNPYCNDLCTKNGAKSGYCQWAGRYGNACWCIDLPDKVPIRISGSCRGR
Measured MW: 7240,24 Da Sequence coverage: 75,29 %
Identified amino acid sequence
c
z
y
b

## Slide 33
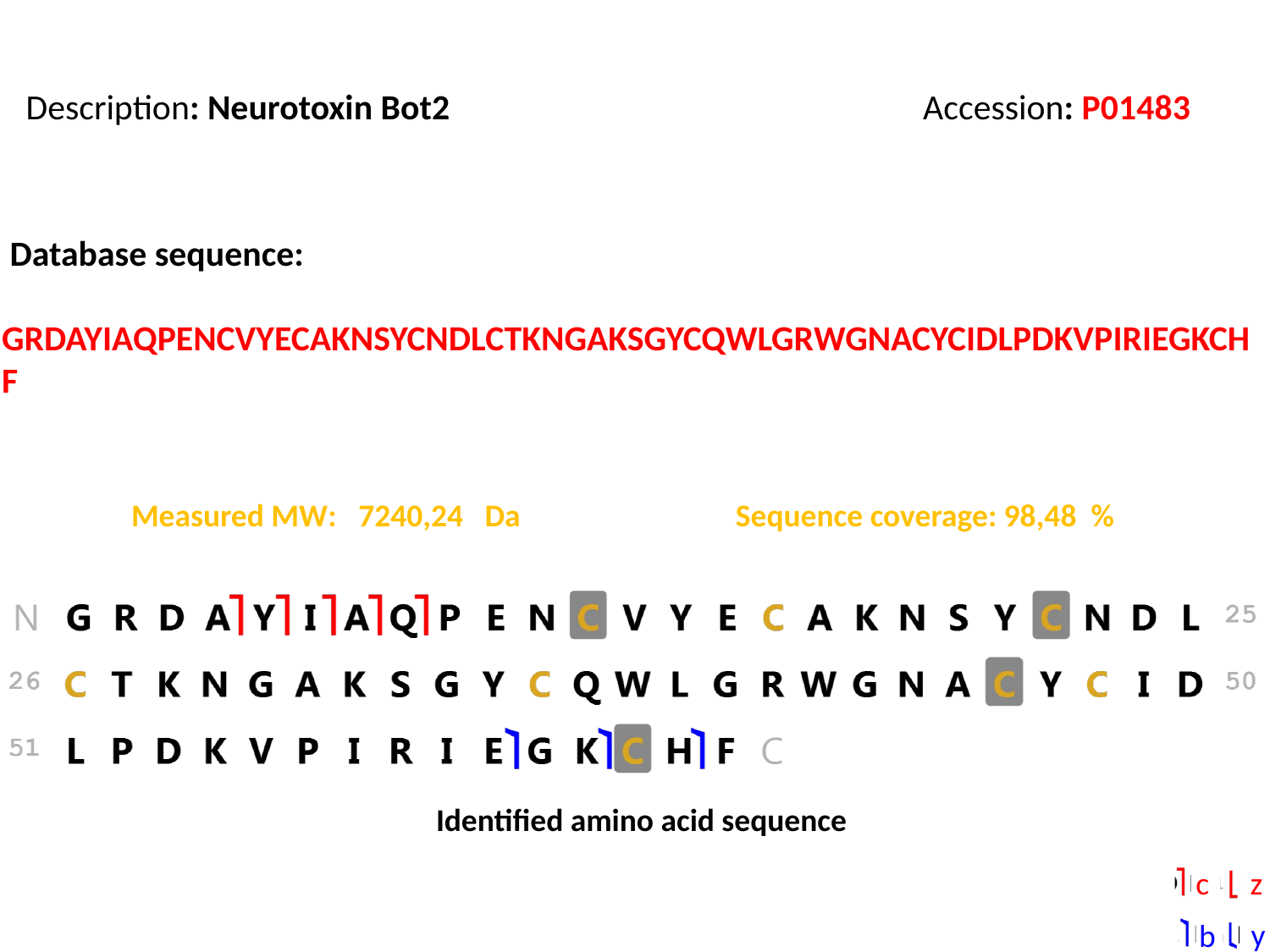

Description: Neurotoxin Bot2 				 Accession: P01483
 Database sequence:
GRDAYIAQPENCVYECAKNSYCNDLCTKNGAKSGYCQWLGRWGNACYCIDLPDKVPIRIEGKCHF
Measured MW: 7240,24 Da Sequence coverage: 98,48 %
Identified amino acid sequence
c
z
y
b

## Slide 34
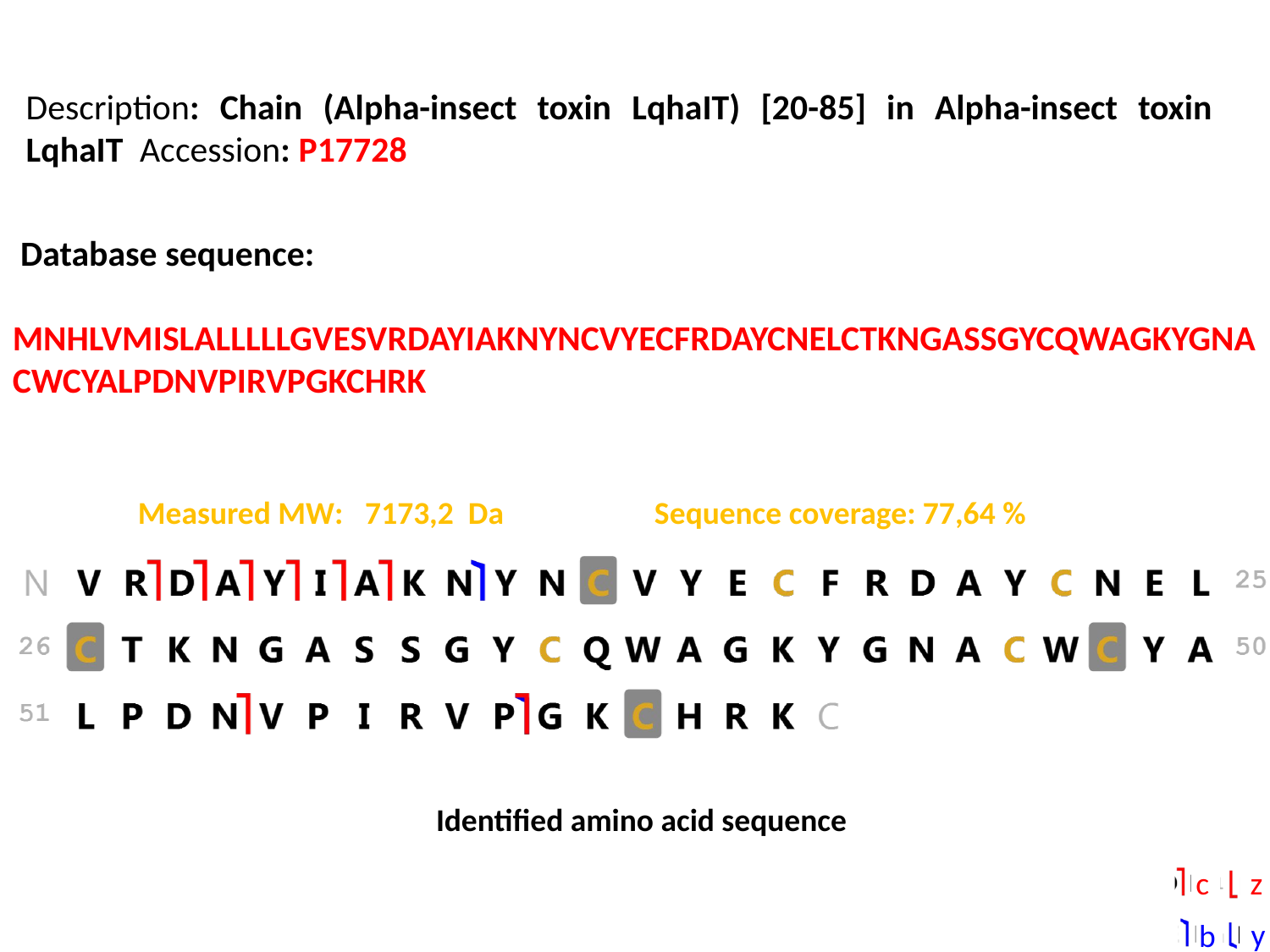

Description: Chain (Alpha-insect toxin LqhaIT) [20-85] in Alpha-insect toxin LqhaIT Accession: P17728
 Database sequence:
MNHLVMISLALLLLLGVESVRDAYIAKNYNCVYECFRDAYCNELCTKNGASSGYCQWAGKYGNACWCYALPDNVPIRVPGKCHRK
Measured MW: 7173,2 Da Sequence coverage: 77,64 %
Identified amino acid sequence
c
z
y
b

## Slide 35
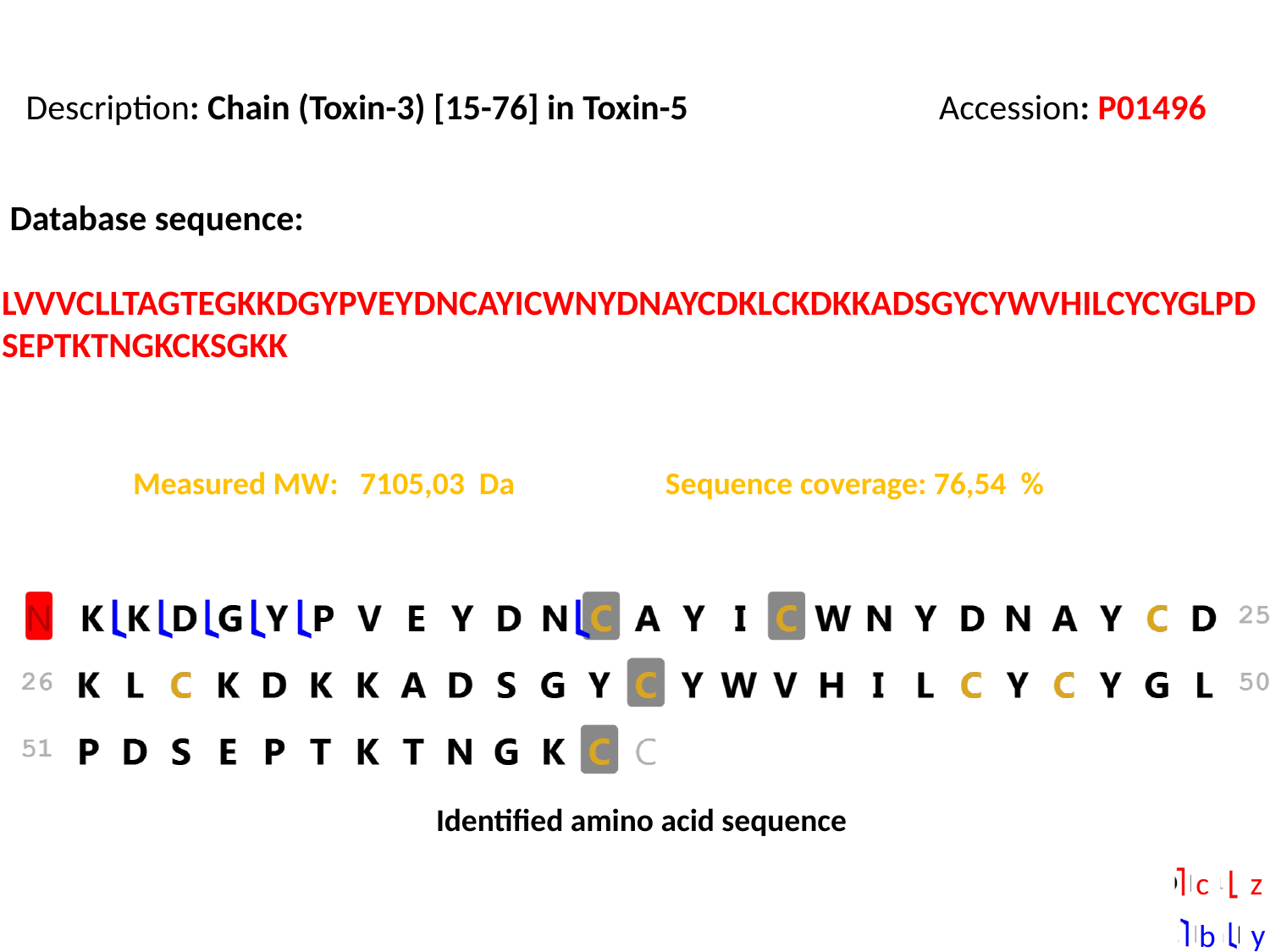

Description: Chain (Toxin-3) [15-76] in Toxin-5		 Accession: P01496
 Database sequence:
LVVVCLLTAGTEGKKDGYPVEYDNCAYICWNYDNAYCDKLCKDKKADSGYCYWVHILCYCYGLPDSEPTKTNGKCKSGKK
Measured MW: 7105,03 Da Sequence coverage: 76,54 %
Identified amino acid sequence
c
z
y
b

## Slide 36
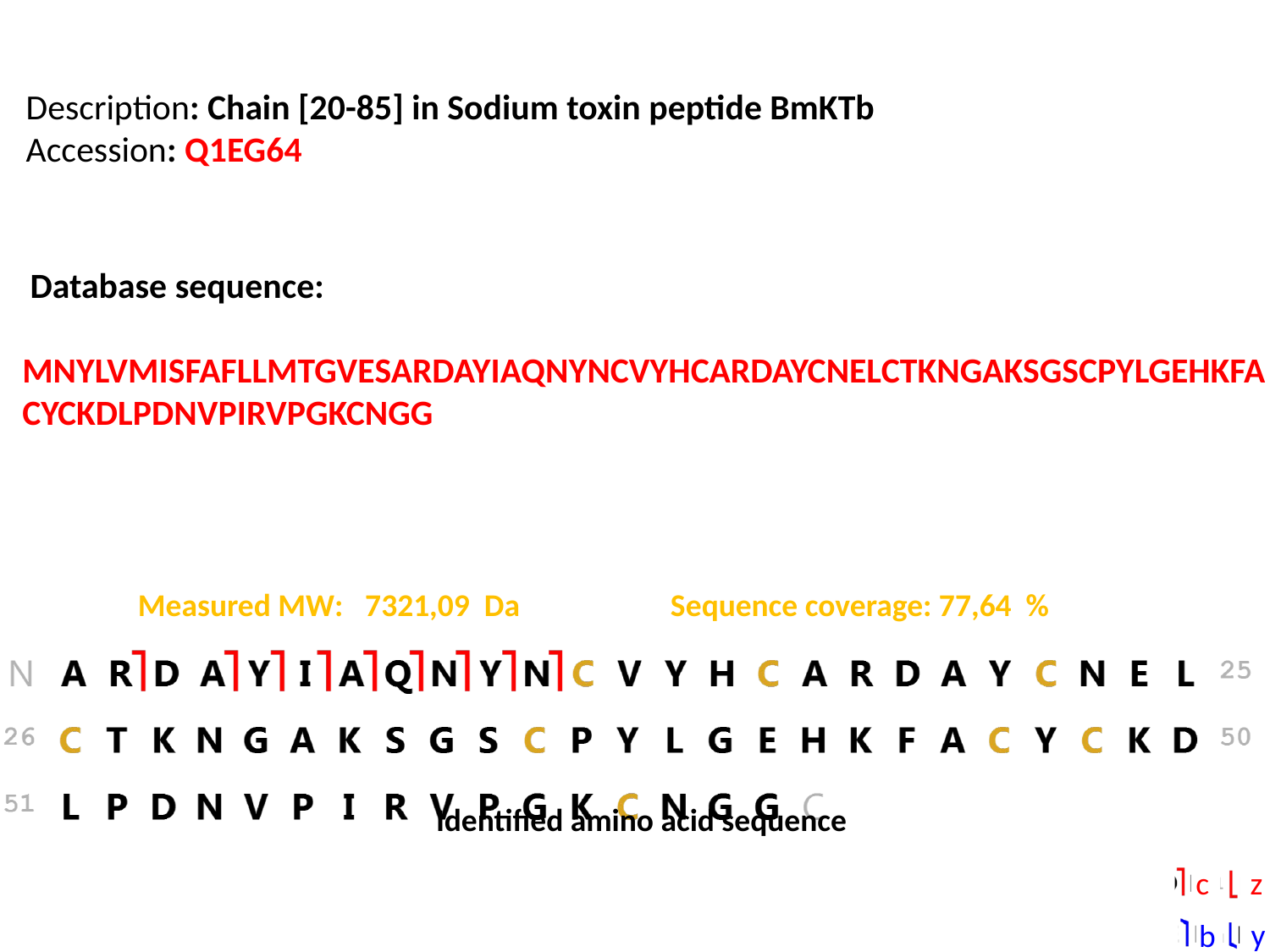

Description: Chain [20-85] in Sodium toxin peptide BmKTb
Accession: Q1EG64
 Database sequence:
MNYLVMISFAFLLMTGVESARDAYIAQNYNCVYHCARDAYCNELCTKNGAKSGSCPYLGEHKFACYCKDLPDNVPIRVPGKCNGG
	'
Measured MW: 7321,09 Da Sequence coverage: 77,64 %
Identified amino acid sequence
c
z
y
b

## Slide 37
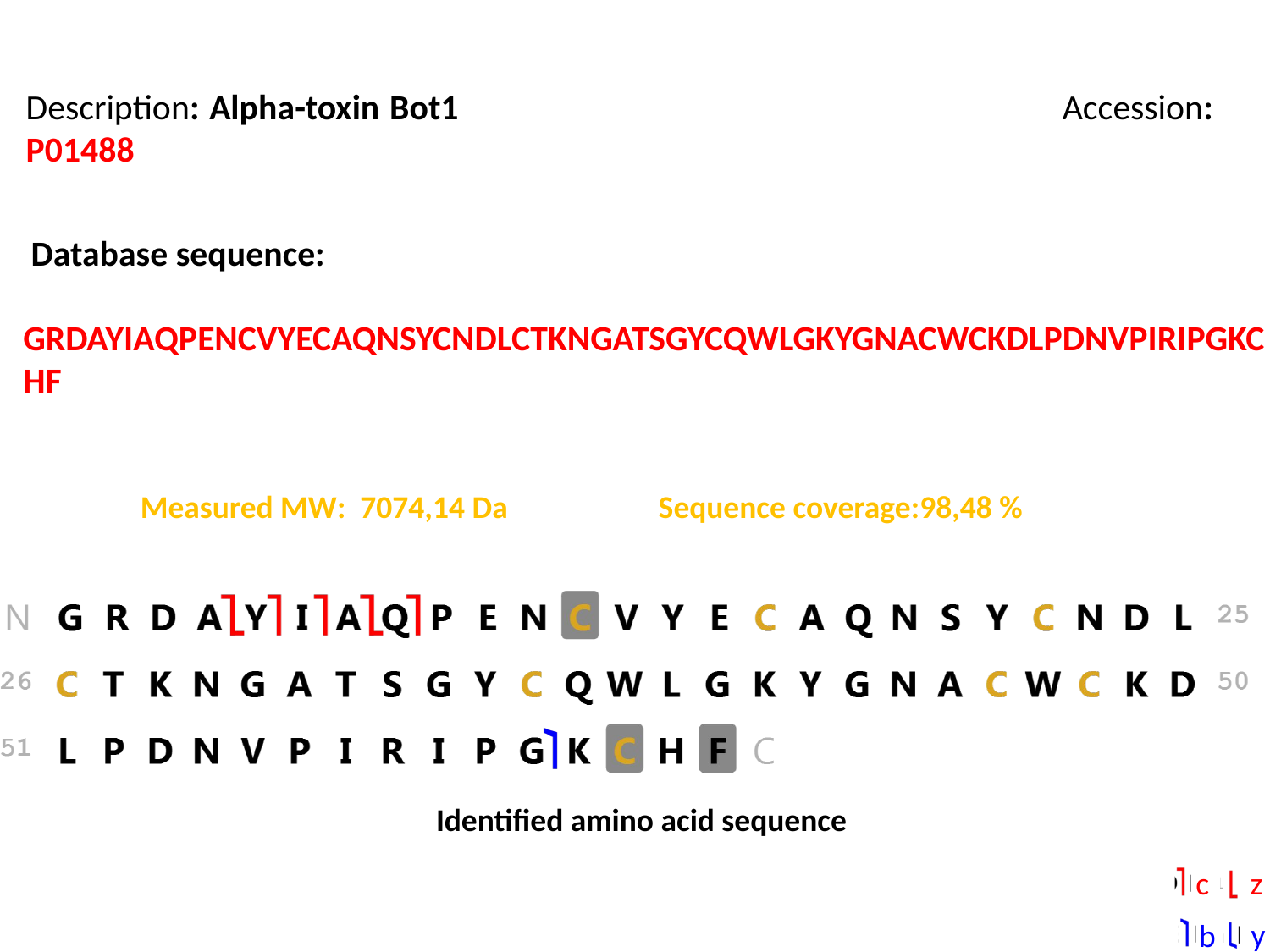

Description: Alpha-toxin Bot1 Accession: P01488
 Database sequence:
GRDAYIAQPENCVYECAQNSYCNDLCTKNGATSGYCQWLGKYGNACWCKDLPDNVPIRIPGKCHF
Measured MW: 7074,14 Da Sequence coverage:98,48 %
Identified amino acid sequence
c
z
y
b

## Slide 38
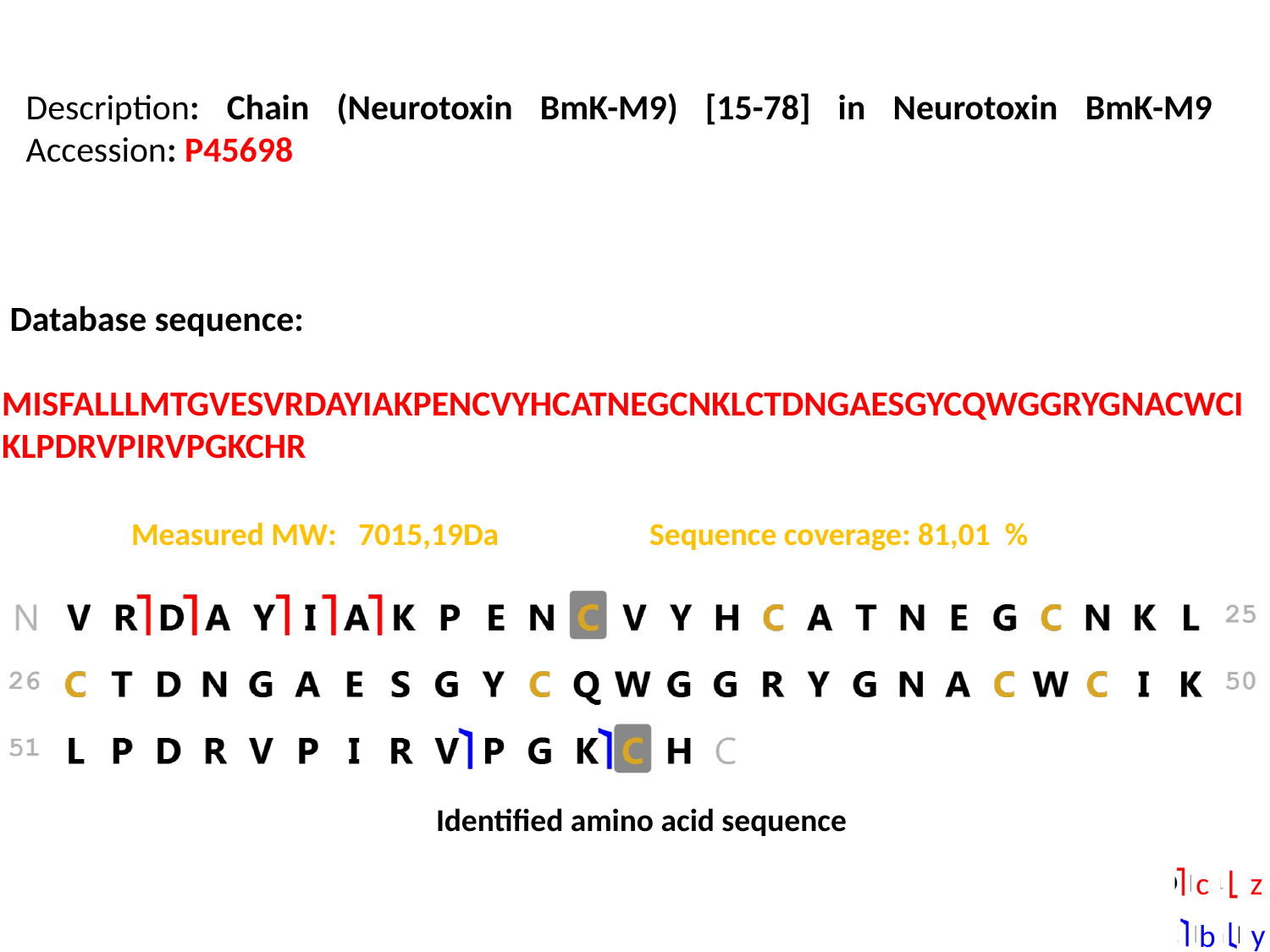

Description: Chain (Neurotoxin BmK-M9) [15-78] in Neurotoxin BmK-M9 Accession: P45698
 Database sequence:
MISFALLLMTGVESVRDAYIAKPENCVYHCATNEGCNKLCTDNGAESGYCQWGGRYGNACWCIKLPDRVPIRVPGKCHR
Measured MW: 7015,19Da Sequence coverage: 81,01 %
Identified amino acid sequence
c
z
y
b

## Slide 39
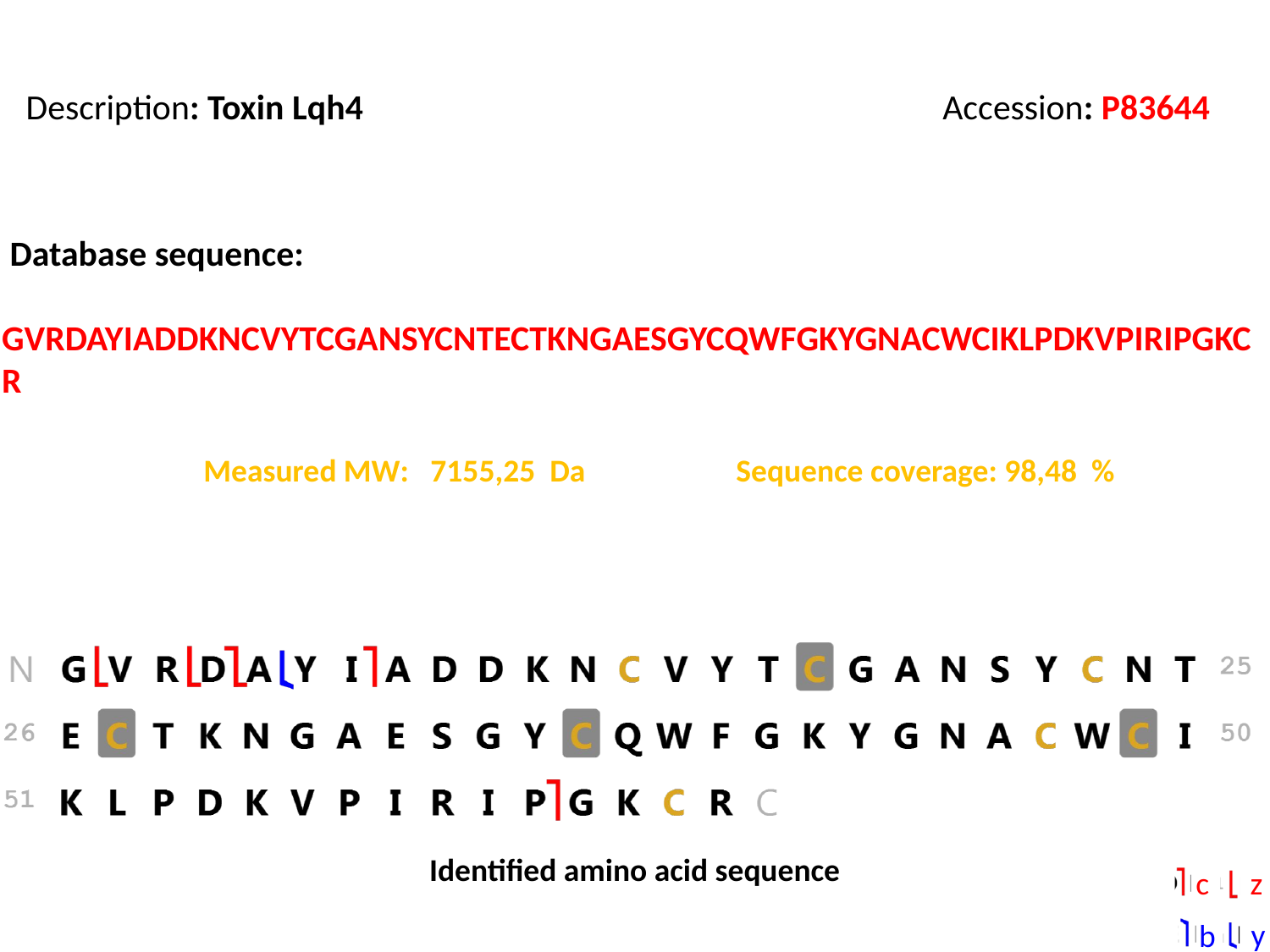

Description: Toxin Lqh4 Accession: P83644
 Database sequence:
GVRDAYIADDKNCVYTCGANSYCNTECTKNGAESGYCQWFGKYGNACWCIKLPDKVPIRIPGKCR
Measured MW: 7155,25 Da Sequence coverage: 98,48 %
Identified amino acid sequence
c
z
y
b

## Slide 40
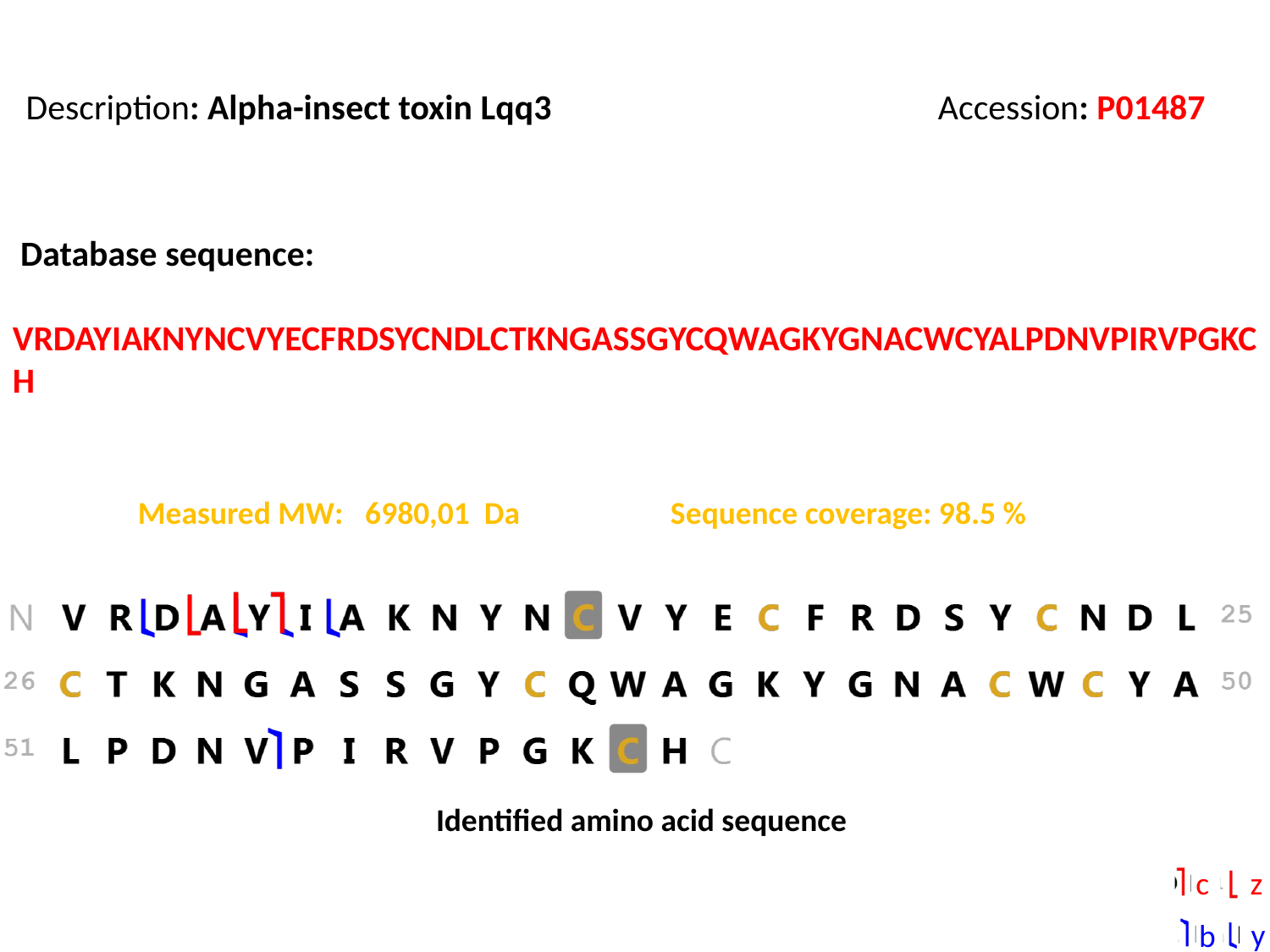

Description: Alpha-insect toxin Lqq3 Accession: P01487
 Database sequence:
VRDAYIAKNYNCVYECFRDSYCNDLCTKNGASSGYCQWAGKYGNACWCYALPDNVPIRVPGKCH
Measured MW: 6980,01 Da Sequence coverage: 98.5 %
Identified amino acid sequence
c
z
y
b

## Slide 41
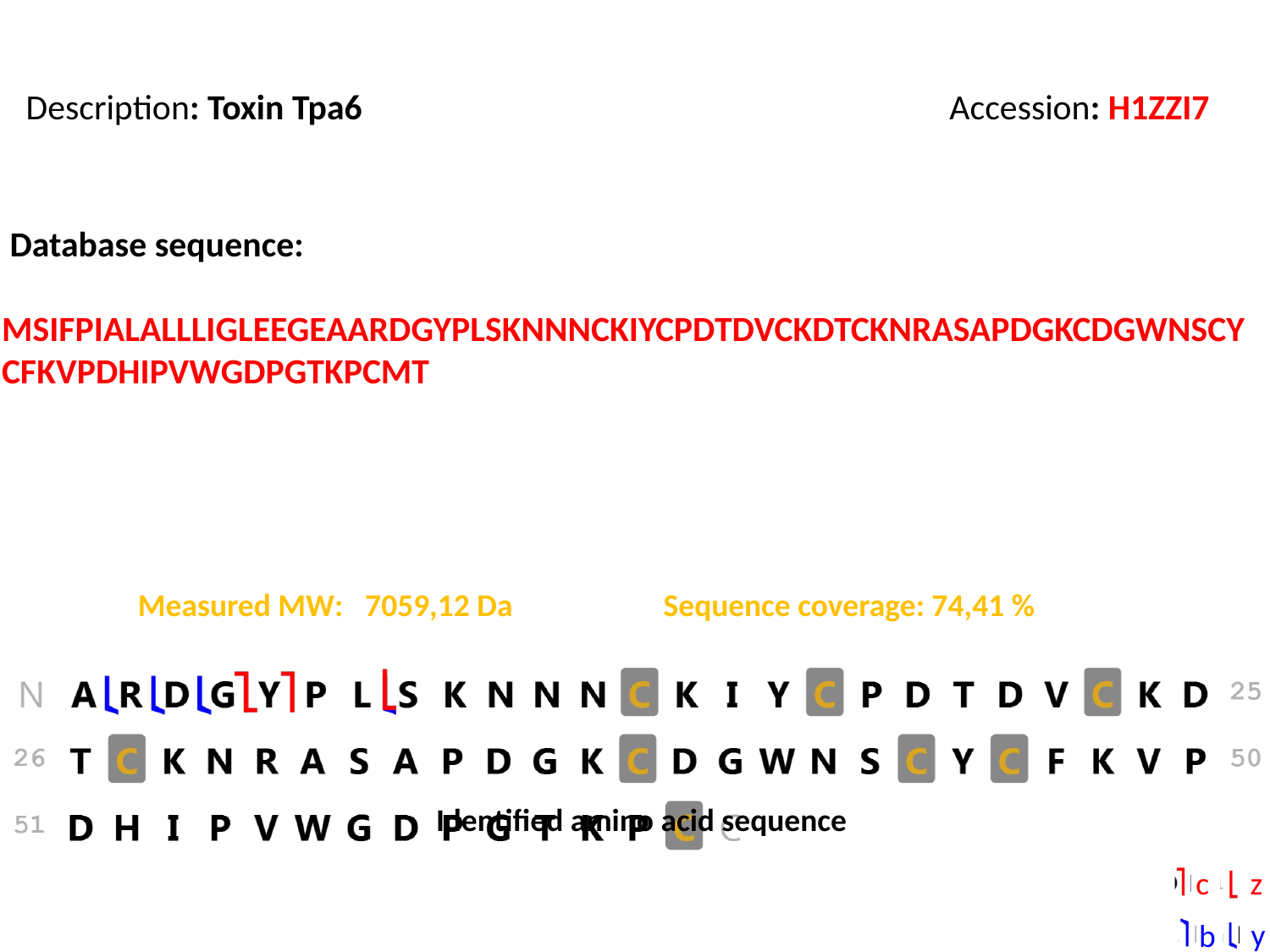

Description: Toxin Tpa6 Accession: H1ZZI7
 Database sequence:
MSIFPIALALLLIGLEEGEAARDGYPLSKNNNCKIYCPDTDVCKDTCKNRASAPDGKCDGWNSCYCFKVPDHIPVWGDPGTKPCMT
Measured MW: 7059,12 Da Sequence coverage: 74,41 %
Identified amino acid sequence
c
z
y
b

## Slide 42
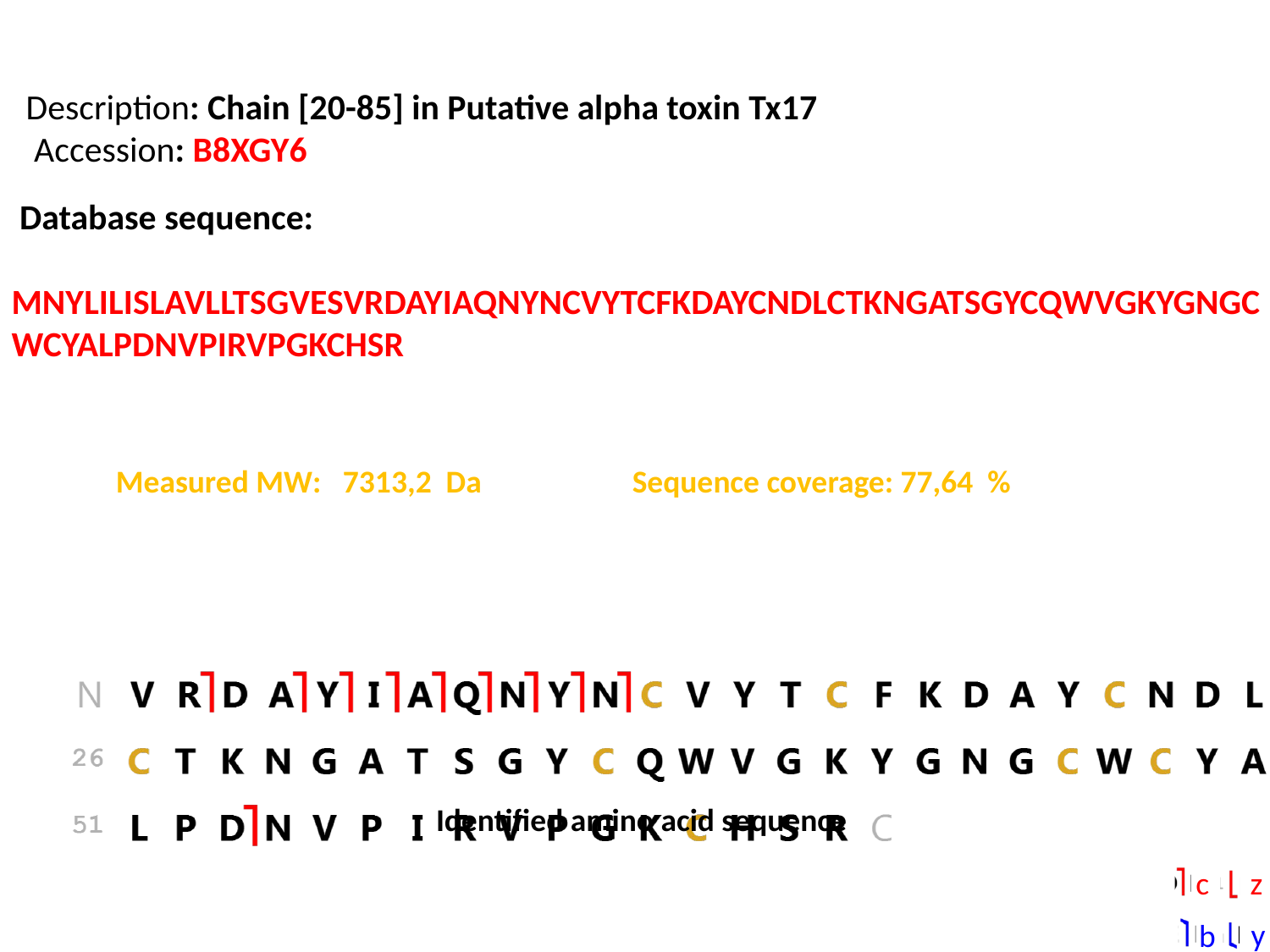

Description: Chain [20-85] in Putative alpha toxin Tx17
 Accession: B8XGY6
 Database sequence:
MNYLILISLAVLLTSGVESVRDAYIAQNYNCVYTCFKDAYCNDLCTKNGATSGYCQWVGKYGNGCWCYALPDNVPIRVPGKCHSR
Measured MW: 7313,2 Da Sequence coverage: 77,64 %
Identified amino acid sequence
c
z
y
b

## Slide 43
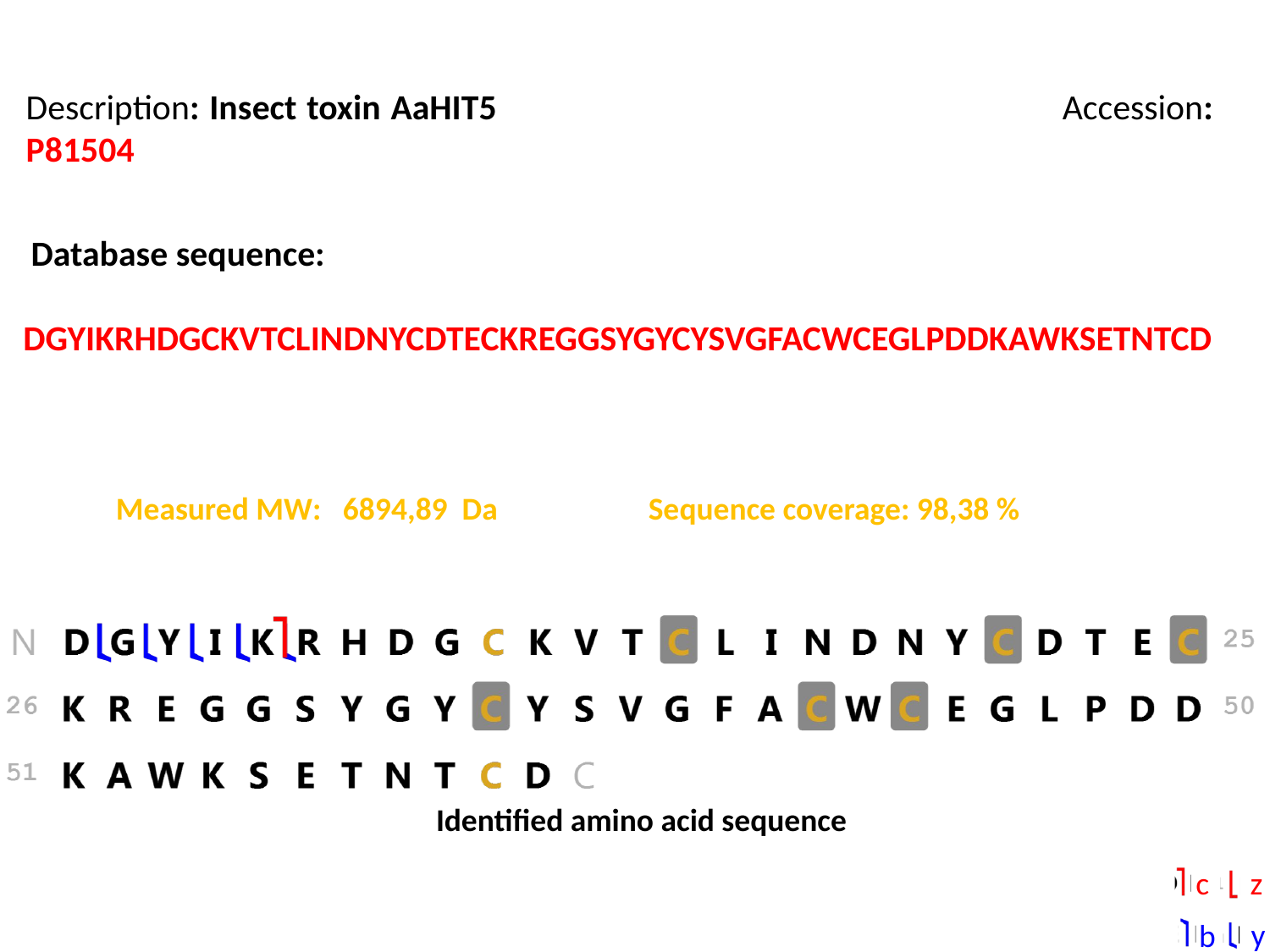

Description: Insect toxin AaHIT5 Accession: P81504
 Database sequence:
DGYIKRHDGCKVTCLINDNYCDTECKREGGSYGYCYSVGFACWCEGLPDDKAWKSETNTCD
Measured MW: 6894,89 Da Sequence coverage: 98,38 %
Identified amino acid sequence
c
z
y
b

## Slide 44
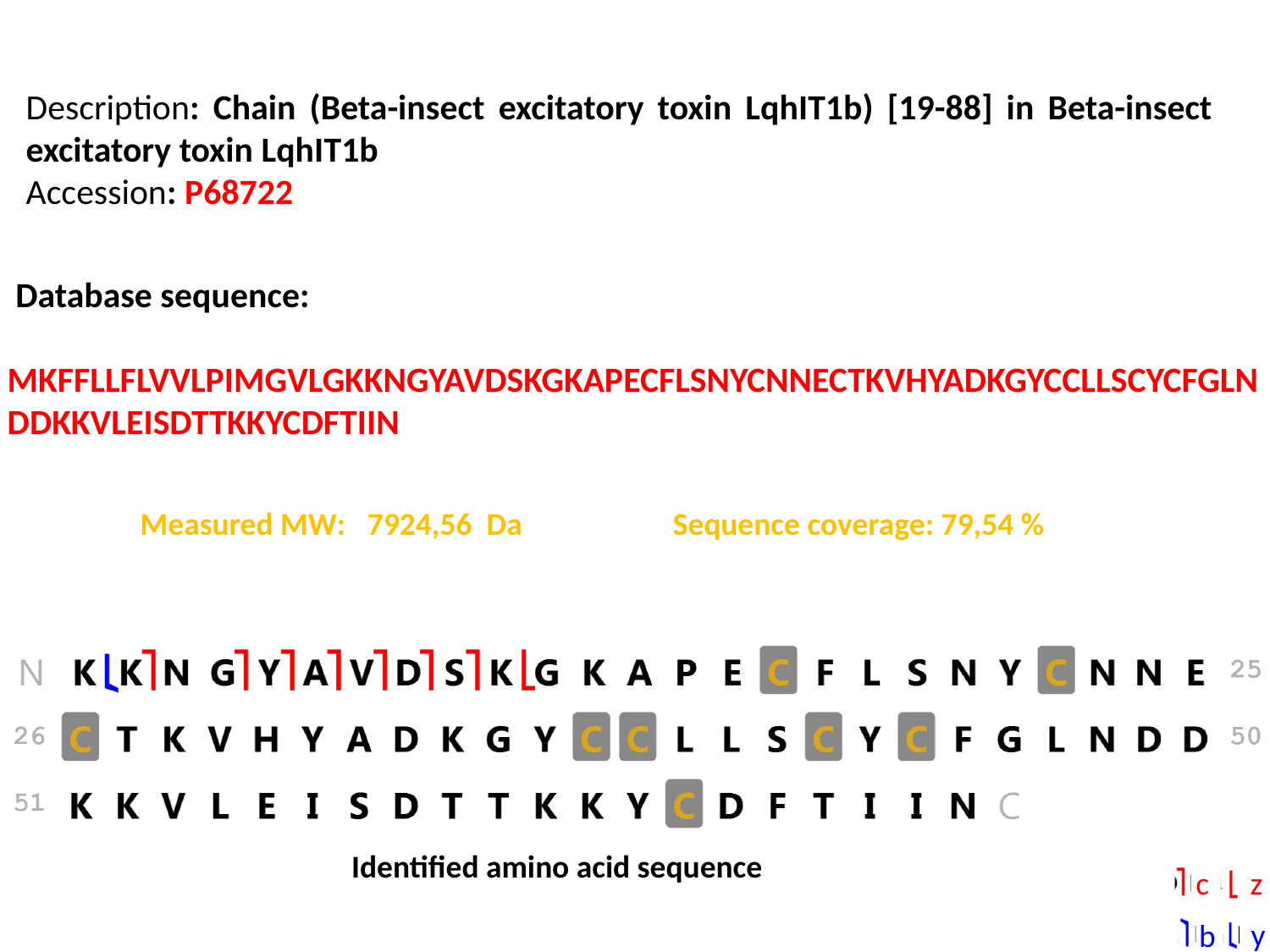

Description: Chain (Beta-insect excitatory toxin LqhIT1b) [19-88] in Beta-insect excitatory toxin LqhIT1b
Accession: P68722
 Database sequence:
MKFFLLFLVVLPIMGVLGKKNGYAVDSKGKAPECFLSNYCNNECTKVHYADKGYCCLLSCYCFGLNDDKKVLEISDTTKKYCDFTIIN
Measured MW: 7924,56 Da Sequence coverage: 79,54 %
Identified amino acid sequence
c
z
y
b

## Slide 45
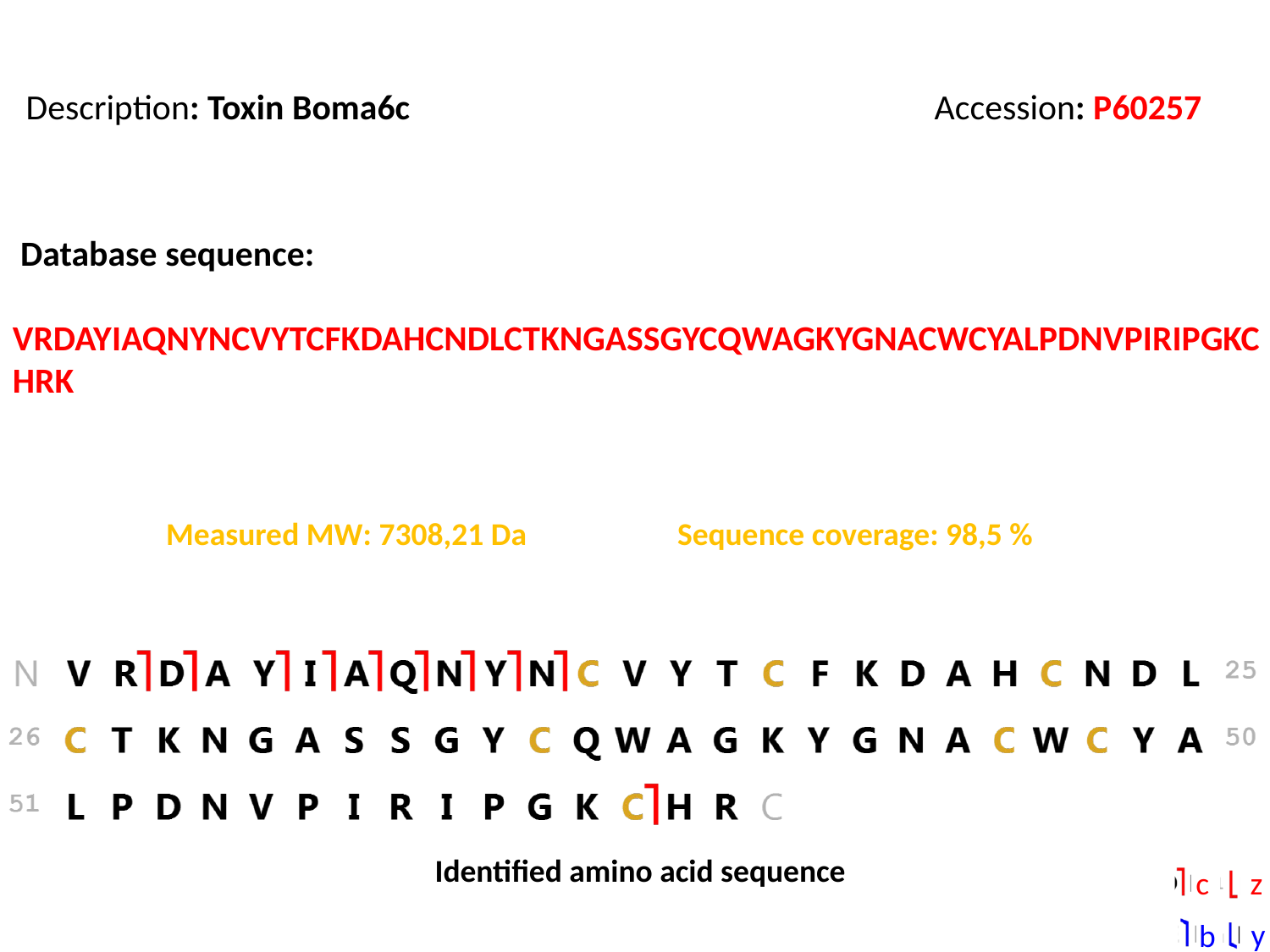

Description: Toxin Boma6c		 Accession: P60257
 Database sequence:
VRDAYIAQNYNCVYTCFKDAHCNDLCTKNGASSGYCQWAGKYGNACWCYALPDNVPIRIPGKCHRK
Measured MW: 7308,21 Da Sequence coverage: 98,5 %
Identified amino acid sequence
c
z
y
b

## Slide 46
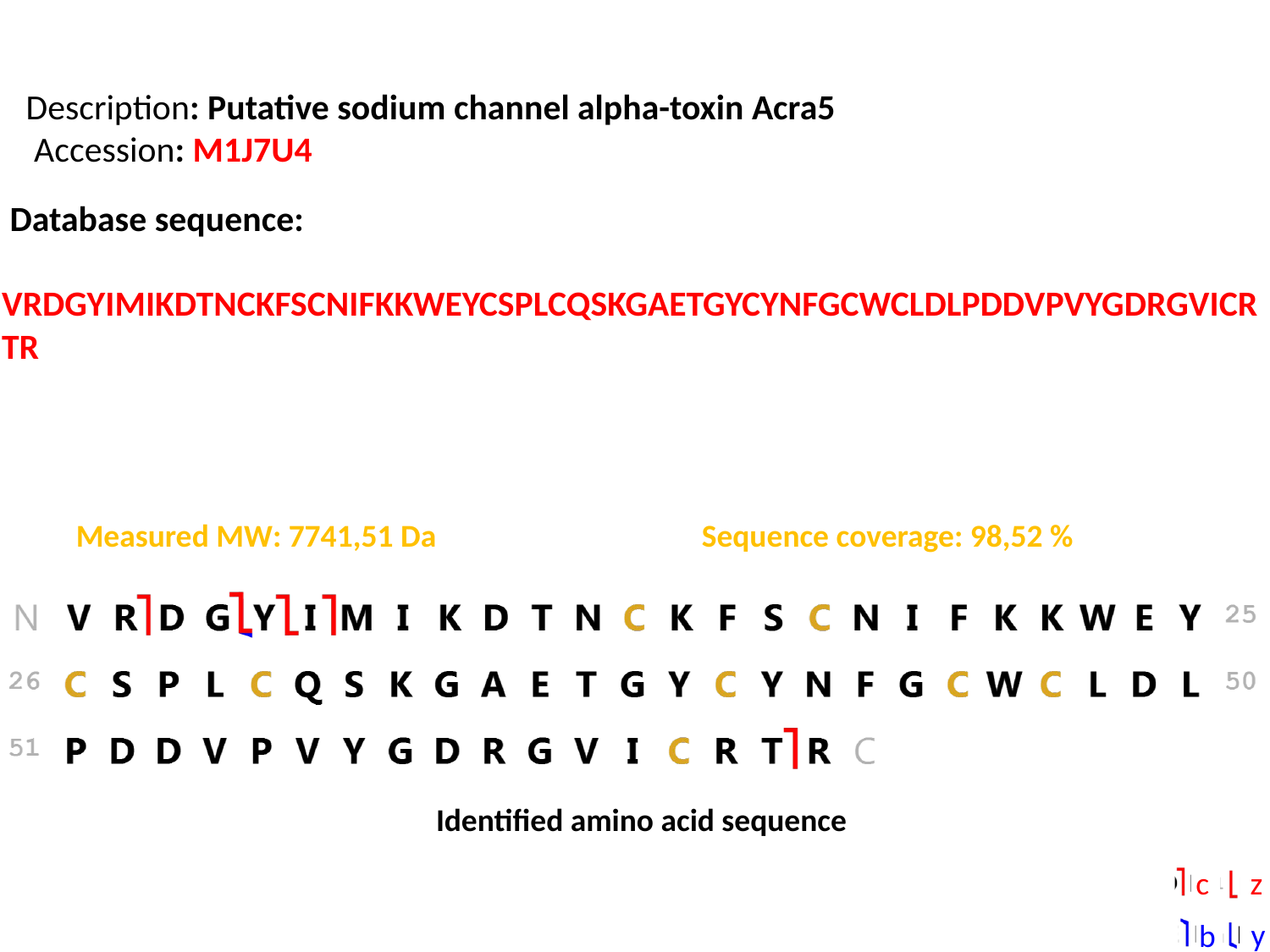

Description: Putative sodium channel alpha-toxin Acra5
 Accession: M1J7U4
 Database sequence:
VRDGYIMIKDTNCKFSCNIFKKWEYCSPLCQSKGAETGYCYNFGCWCLDLPDDVPVYGDRGVICRTR
Measured MW: 7741,51 Da Sequence coverage: 98,52 %
Identified amino acid sequence
c
z
y
b

## Slide 47
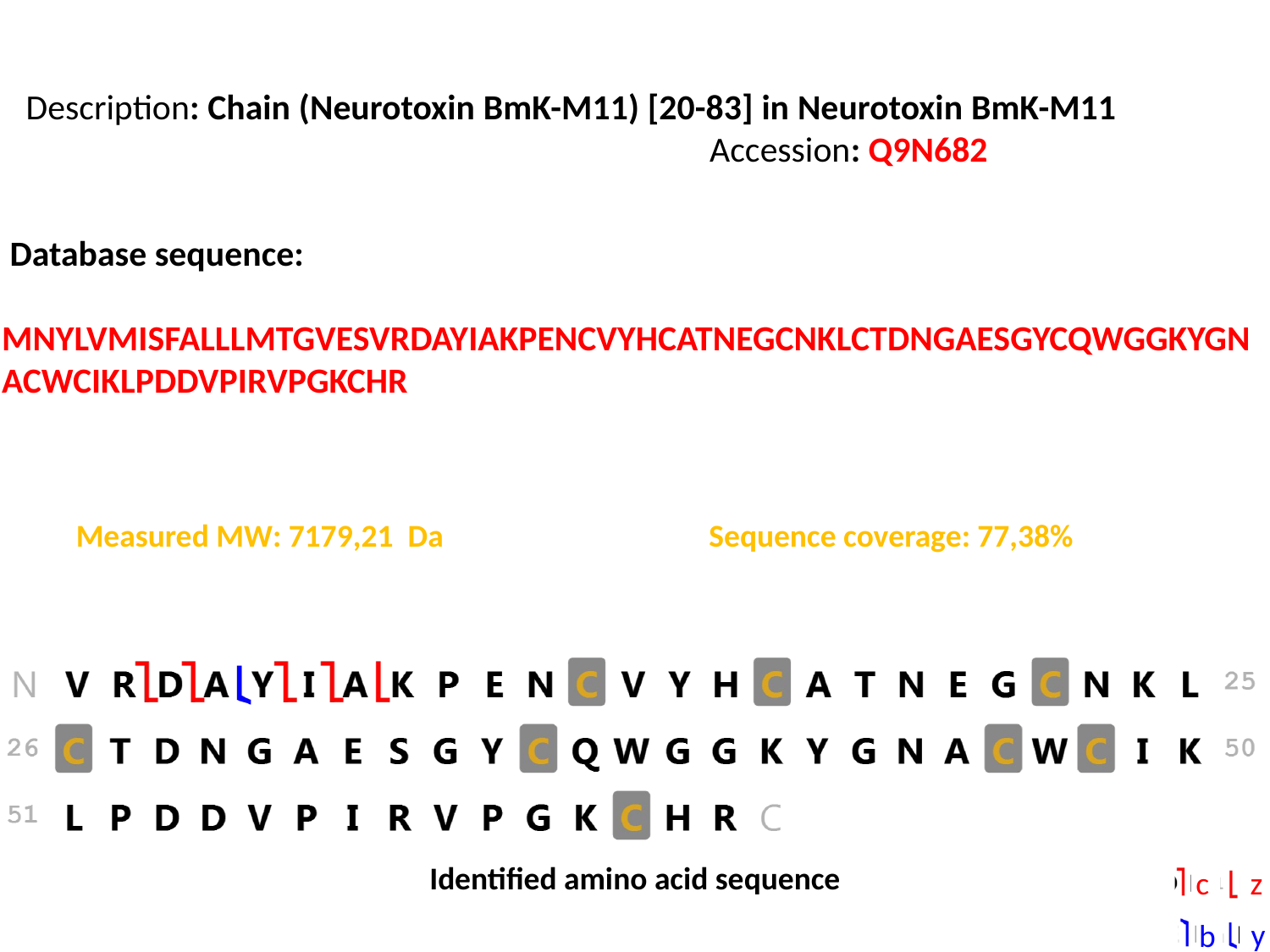

Description: Chain (Neurotoxin BmK-M11) [20-83] in Neurotoxin BmK-M11	 Accession: Q9N682
 Database sequence:
MNYLVMISFALLLMTGVESVRDAYIAKPENCVYHCATNEGCNKLCTDNGAESGYCQWGGKYGNACWCIKLPDDVPIRVPGKCHR
Measured MW: 7179,21 Da Sequence coverage: 77,38%
Identified amino acid sequence
c
z
y
b

## Slide 48
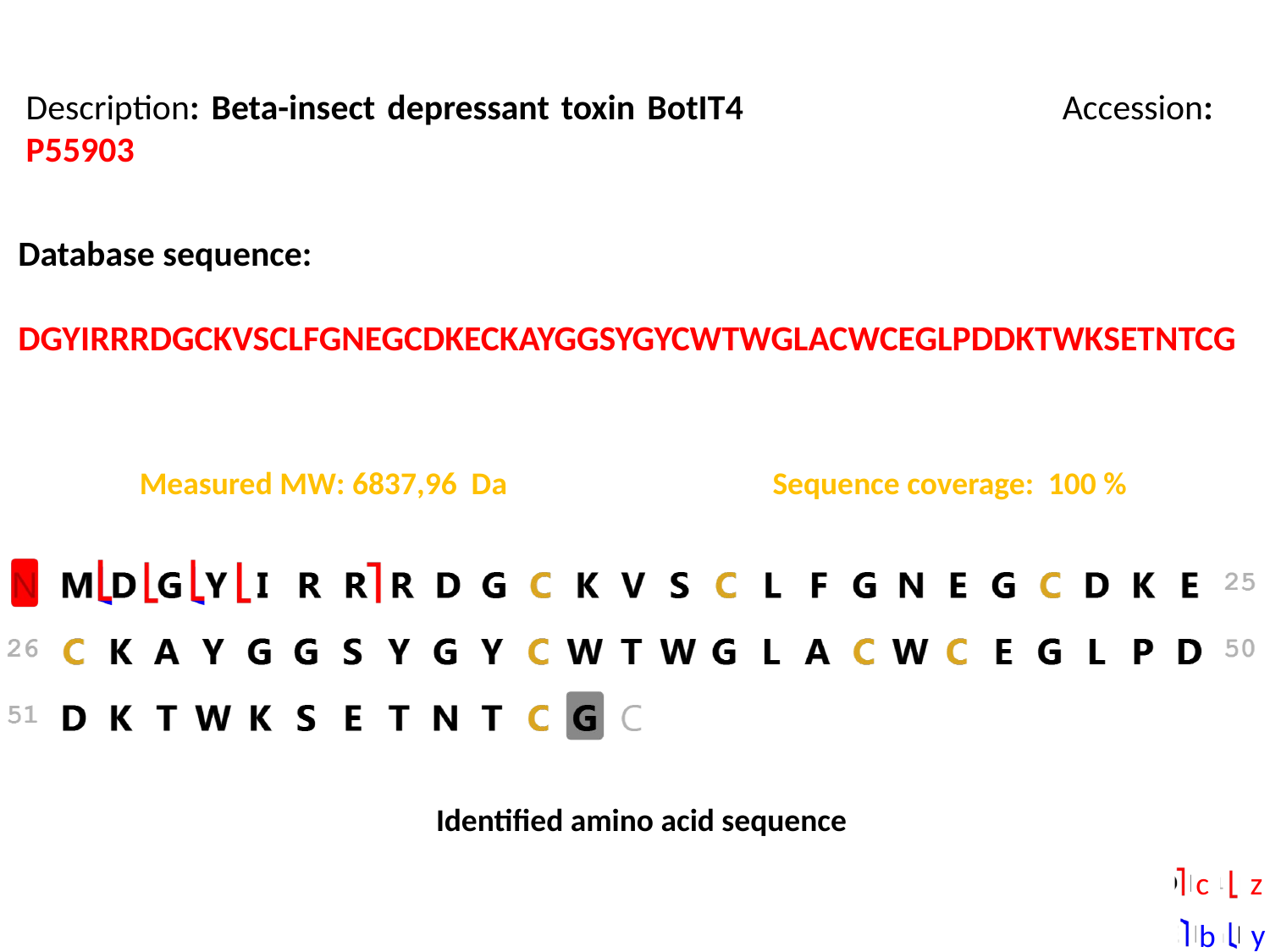

Description: Beta-insect depressant toxin BotIT4 Accession: P55903
 Database sequence:
 DGYIRRRDGCKVSCLFGNEGCDKECKAYGGSYGYCWTWGLACWCEGLPDDKTWKSETNTCG
Measured MW: 6837,96 Da Sequence coverage: 100 %
Identified amino acid sequence
c
z
y
b

## Slide 49
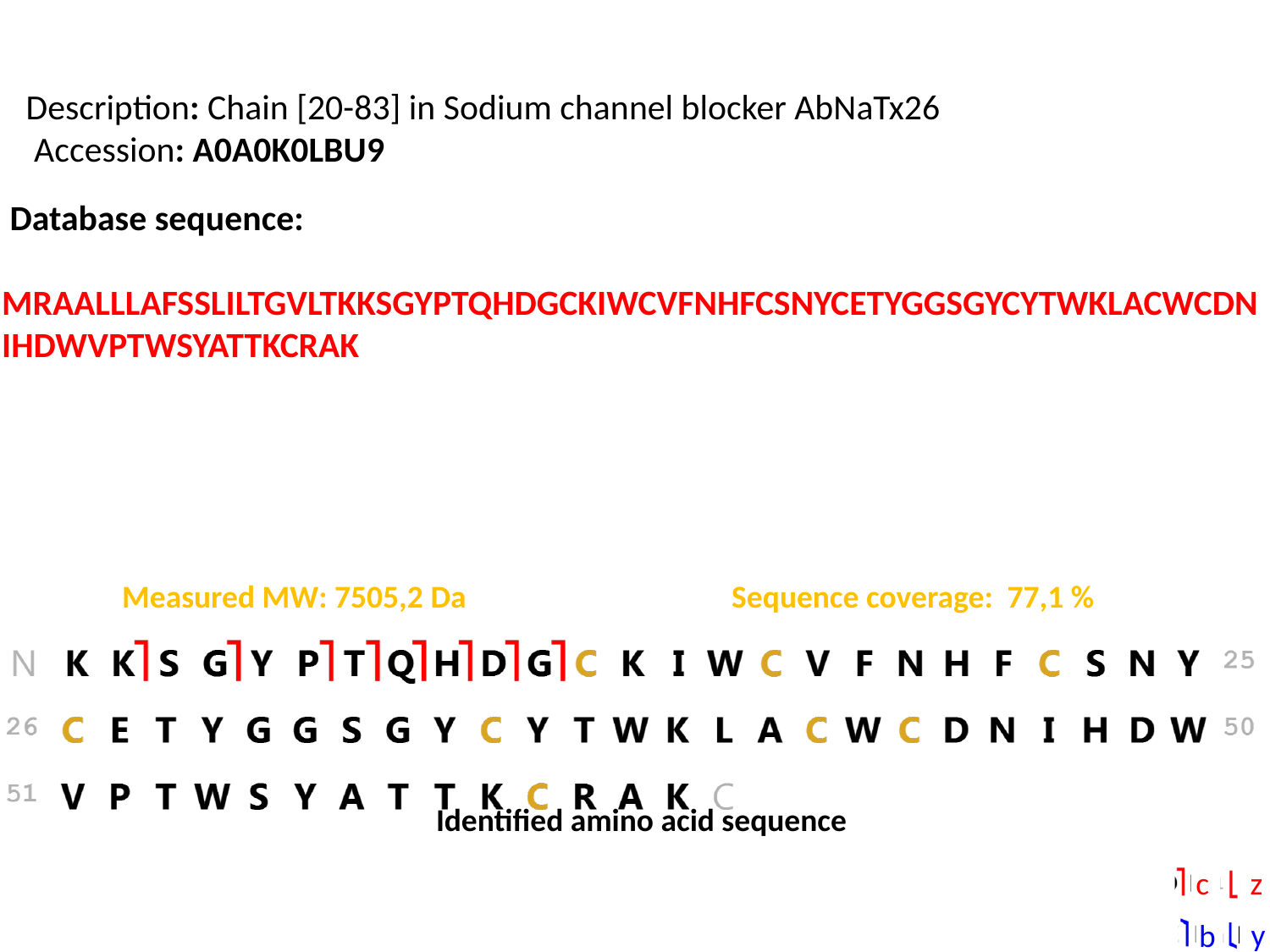

Description: Chain [20-83] in Sodium channel blocker AbNaTx26
 Accession: A0A0K0LBU9
 Database sequence:
MRAALLLAFSSLILTGVLTKKSGYPTQHDGCKIWCVFNHFCSNYCETYGGSGYCYTWKLACWCDNIHDWVPTWSYATTKCRAK
Measured MW: 7505,2 Da Sequence coverage: 77,1 %
Identified amino acid sequence
c
z
y
b

## Slide 50
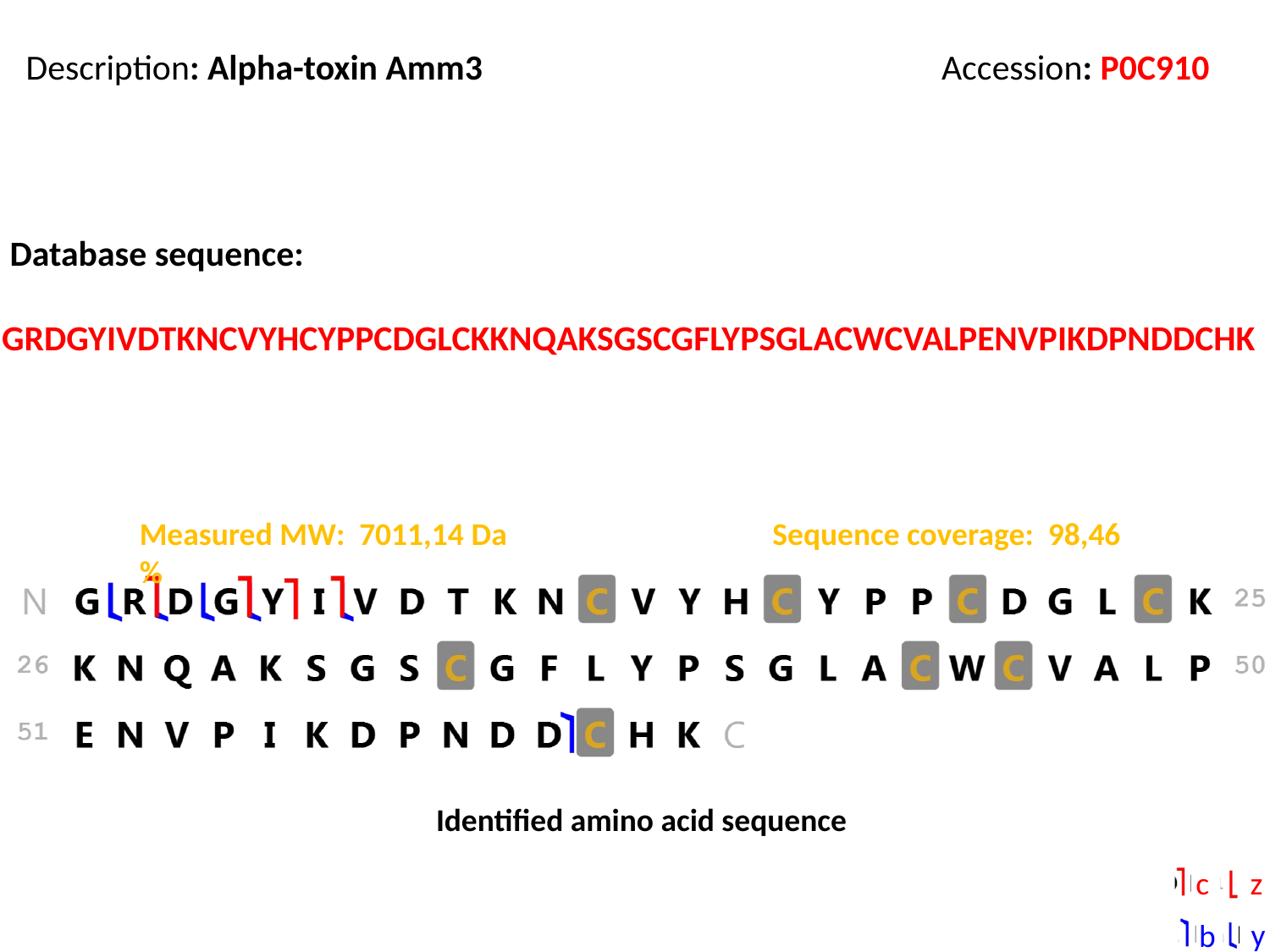

Description: Alpha-toxin Amm3 Accession: P0C910
 Database sequence:
GRDGYIVDTKNCVYHCYPPCDGLCKKNQAKSGSCGFLYPSGLACWCVALPENVPIKDPNDDCHK
Measured MW: 7011,14 Da Sequence coverage: 98,46 %
Identified amino acid sequence
c
z
y
b

## Slide 51
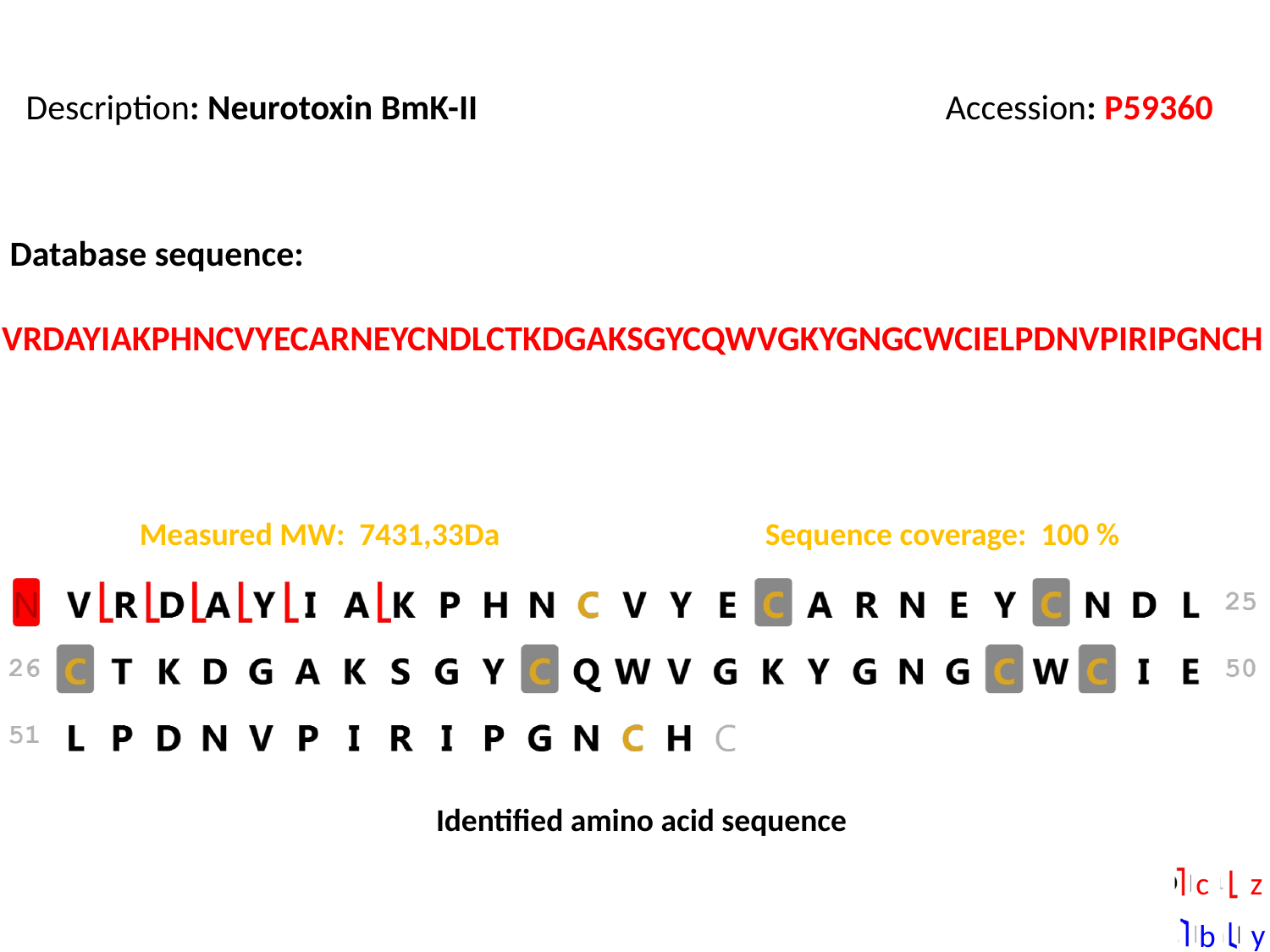

Description: Neurotoxin BmK-II Accession: P59360
 Database sequence:
VRDAYIAKPHNCVYECARNEYCNDLCTKDGAKSGYCQWVGKYGNGCWCIELPDNVPIRIPGNCH
Measured MW: 7431,33Da Sequence coverage: 100 %
Identified amino acid sequence
c
z
y
b

## Slide 52
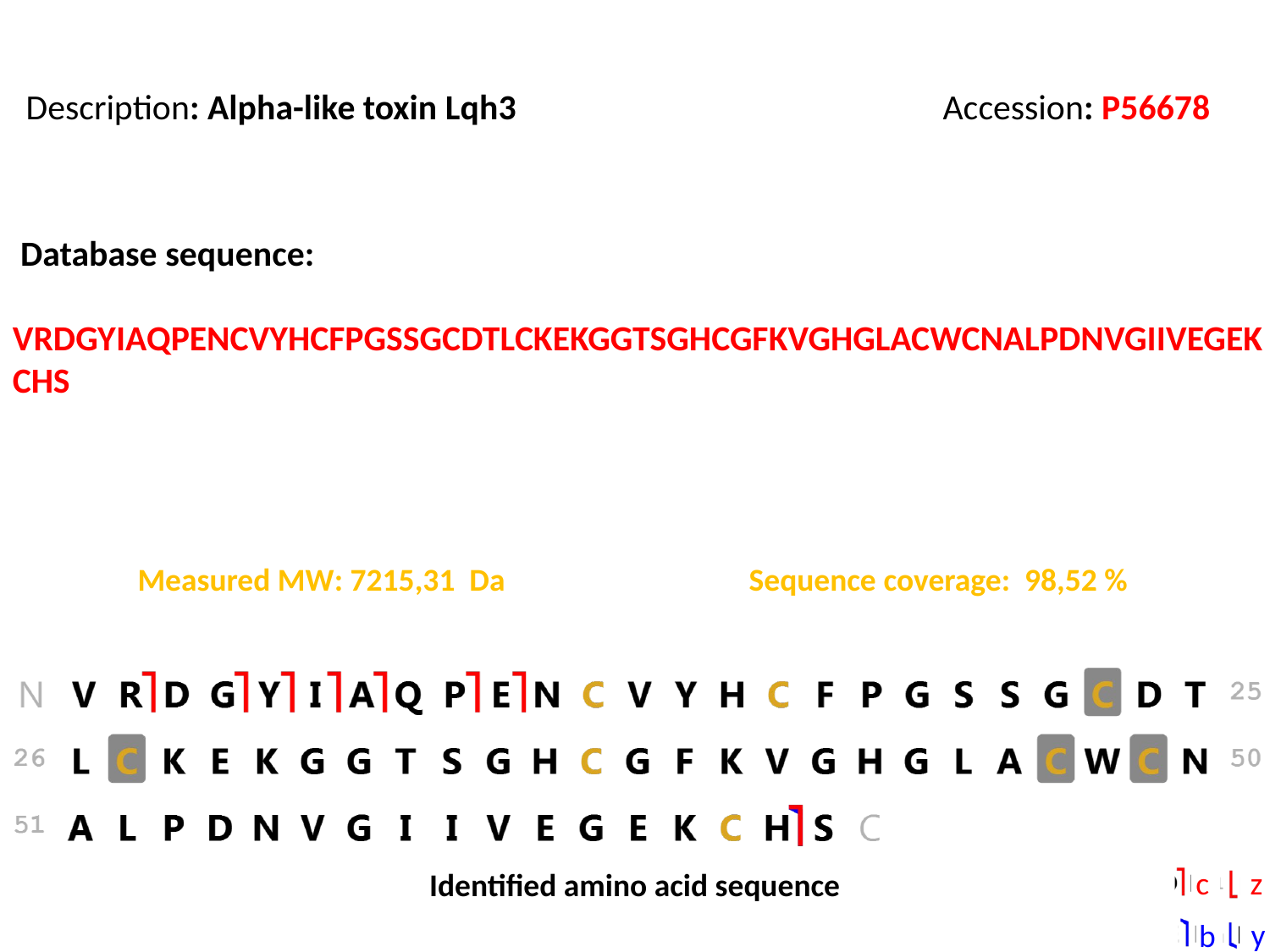

Description: Alpha-like toxin Lqh3 Accession: P56678
 Database sequence:
VRDGYIAQPENCVYHCFPGSSGCDTLCKEKGGTSGHCGFKVGHGLACWCNALPDNVGIIVEGEKCHS
Measured MW: 7215,31 Da Sequence coverage: 98,52 %
c
z
y
b
Identified amino acid sequence

## Slide 53
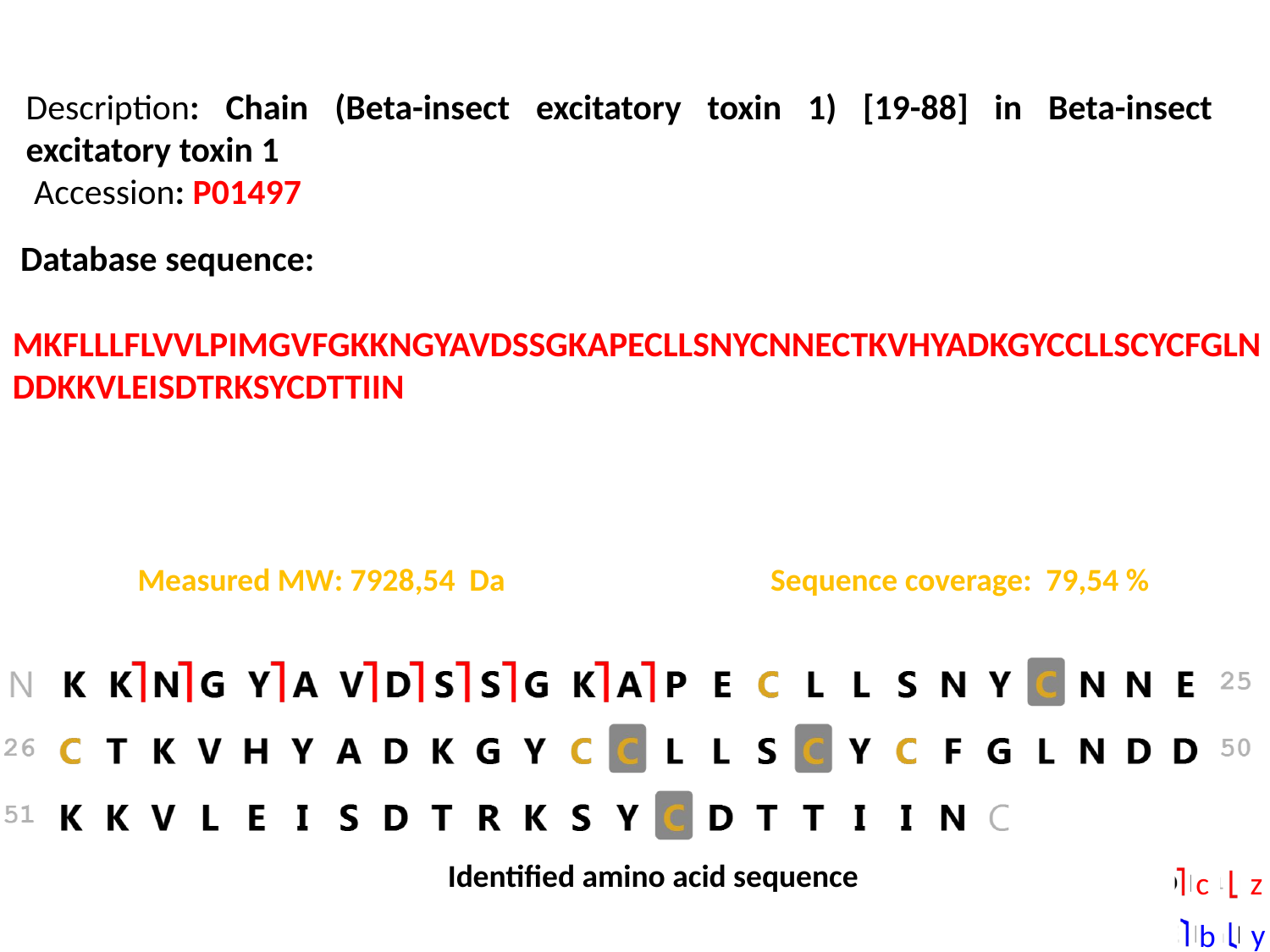

Description: Chain (Beta-insect excitatory toxin 1) [19-88] in Beta-insect excitatory toxin 1
 Accession: P01497
 Database sequence:
MKFLLLFLVVLPIMGVFGKKNGYAVDSSGKAPECLLSNYCNNECTKVHYADKGYCCLLSCYCFGLNDDKKVLEISDTRKSYCDTTIIN
Measured MW: 7928,54 Da Sequence coverage: 79,54 %
Identified amino acid sequence
c
z
y
b

## Slide 54
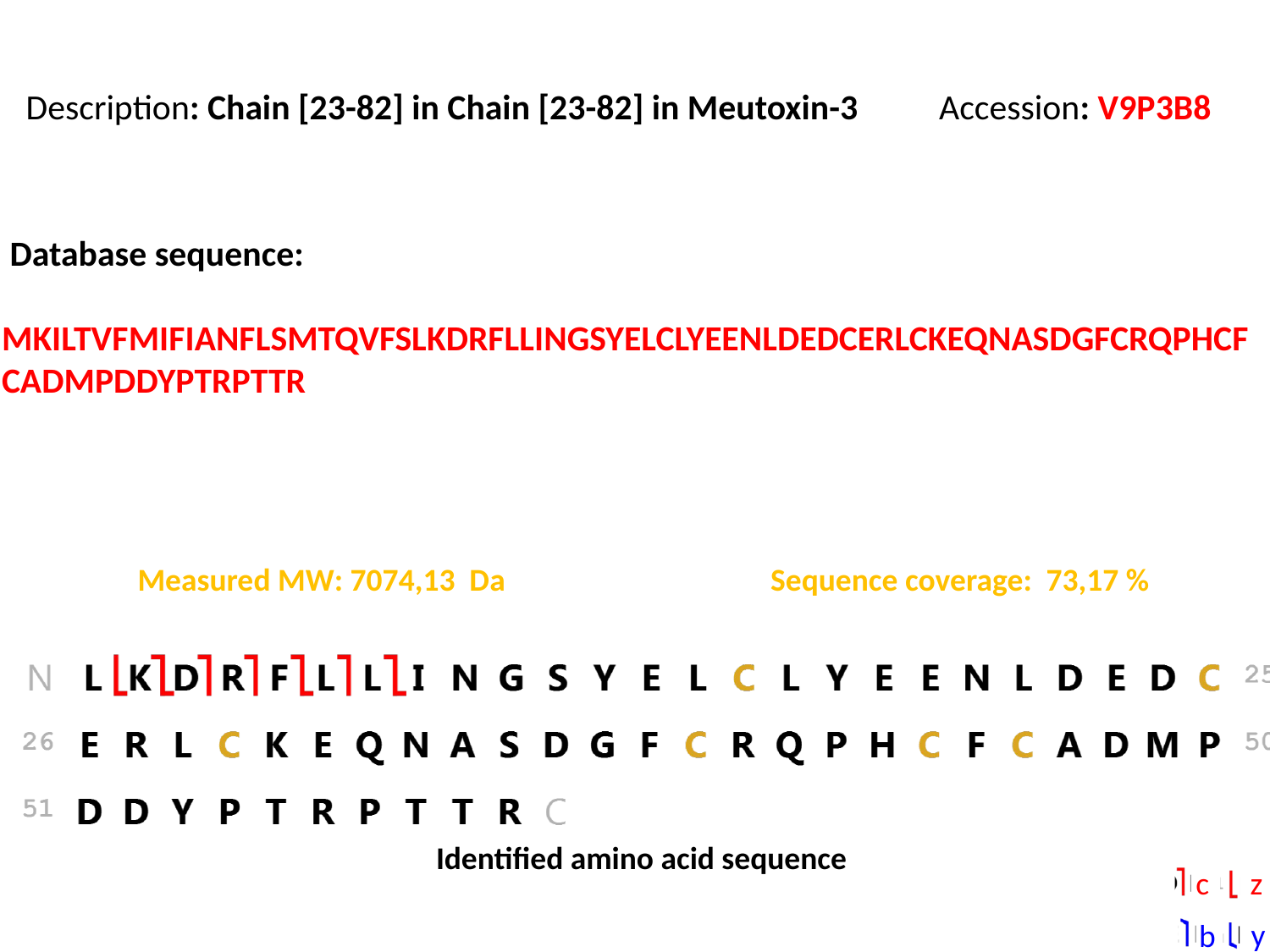

Description: Chain [23-82] in Chain [23-82] in Meutoxin-3 Accession: V9P3B8
 Database sequence:
MKILTVFMIFIANFLSMTQVFSLKDRFLLINGSYELCLYEENLDEDCERLCKEQNASDGFCRQPHCFCADMPDDYPTRPTTR
Measured MW: 7074,13 Da Sequence coverage: 73,17 %
Identified amino acid sequence
c
z
y
b

## Slide 55
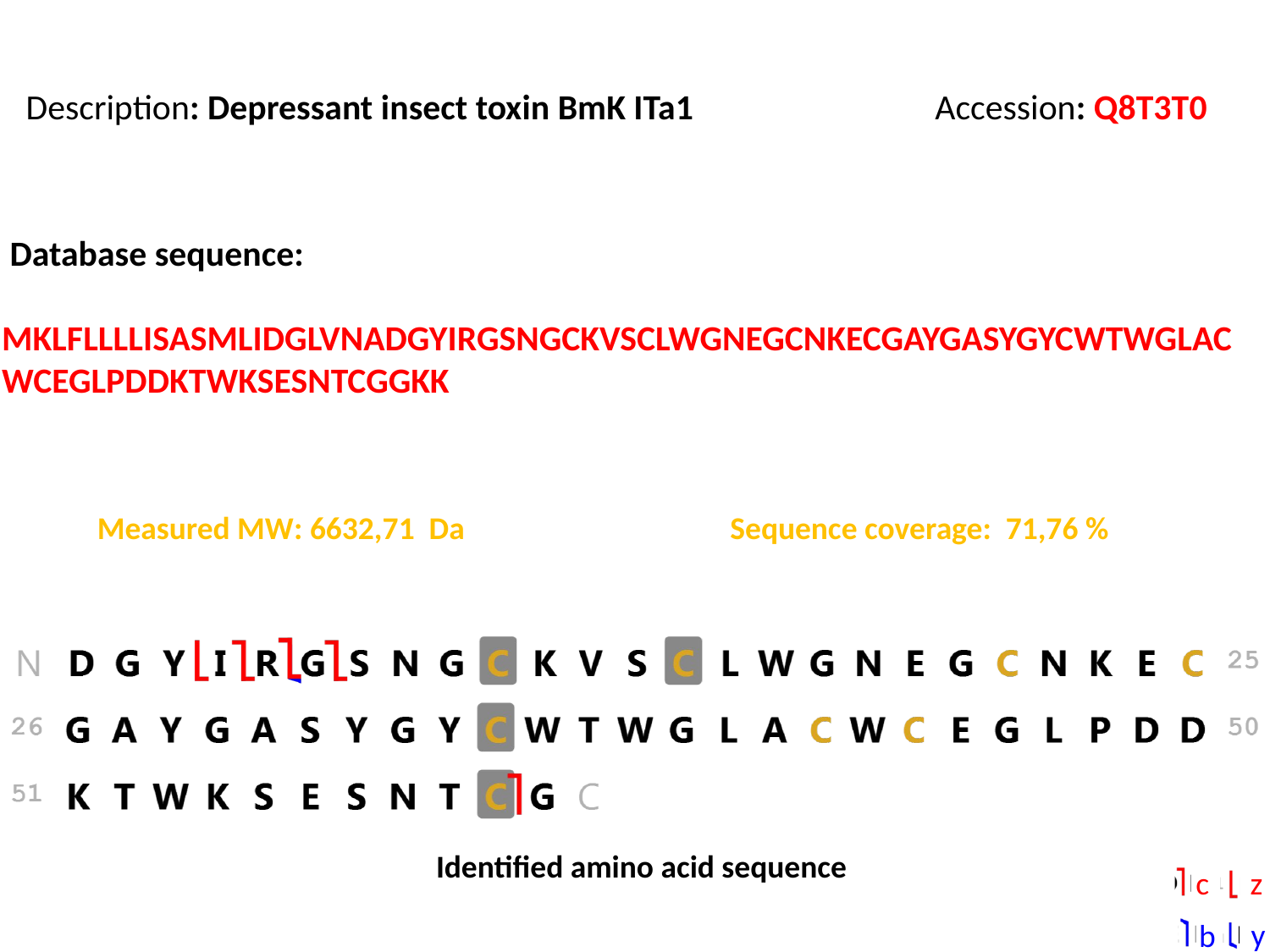

Description: Depressant insect toxin BmK ITa1 Accession: Q8T3T0
 Database sequence:
MKLFLLLLISASMLIDGLVNADGYIRGSNGCKVSCLWGNEGCNKECGAYGASYGYCWTWGLACWCEGLPDDKTWKSESNTCGGKK
Measured MW: 6632,71 Da Sequence coverage: 71,76 %
Identified amino acid sequence
c
z
y
b

## Slide 56
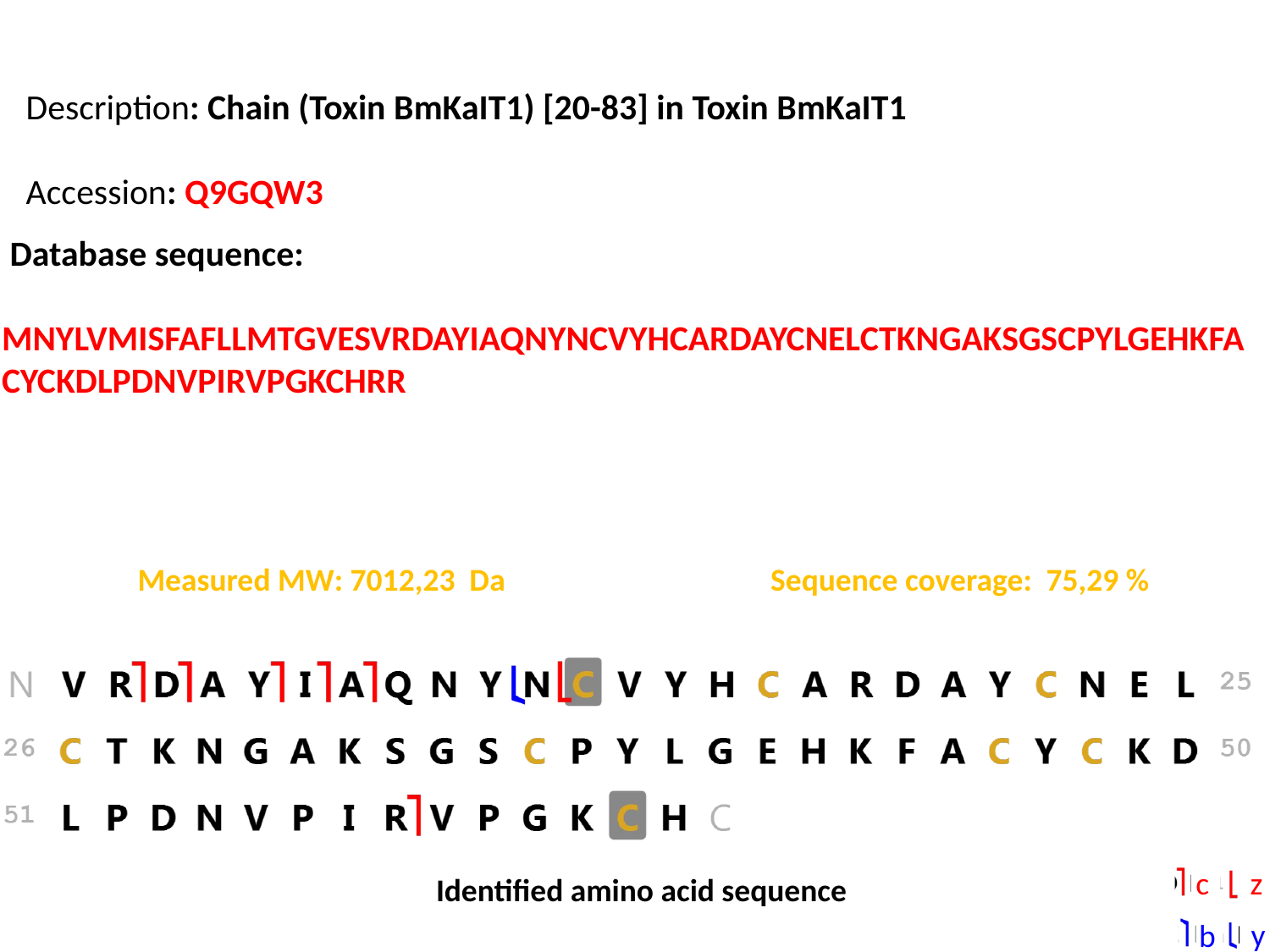

Description: Chain (Toxin BmKaIT1) [20-83] in Toxin BmKaIT1
Accession: Q9GQW3
 Database sequence:
MNYLVMISFAFLLMTGVESVRDAYIAQNYNCVYHCARDAYCNELCTKNGAKSGSCPYLGEHKFACYCKDLPDNVPIRVPGKCHRR
Measured MW: 7012,23 Da Sequence coverage: 75,29 %
c
z
y
b
Identified amino acid sequence

## Slide 57
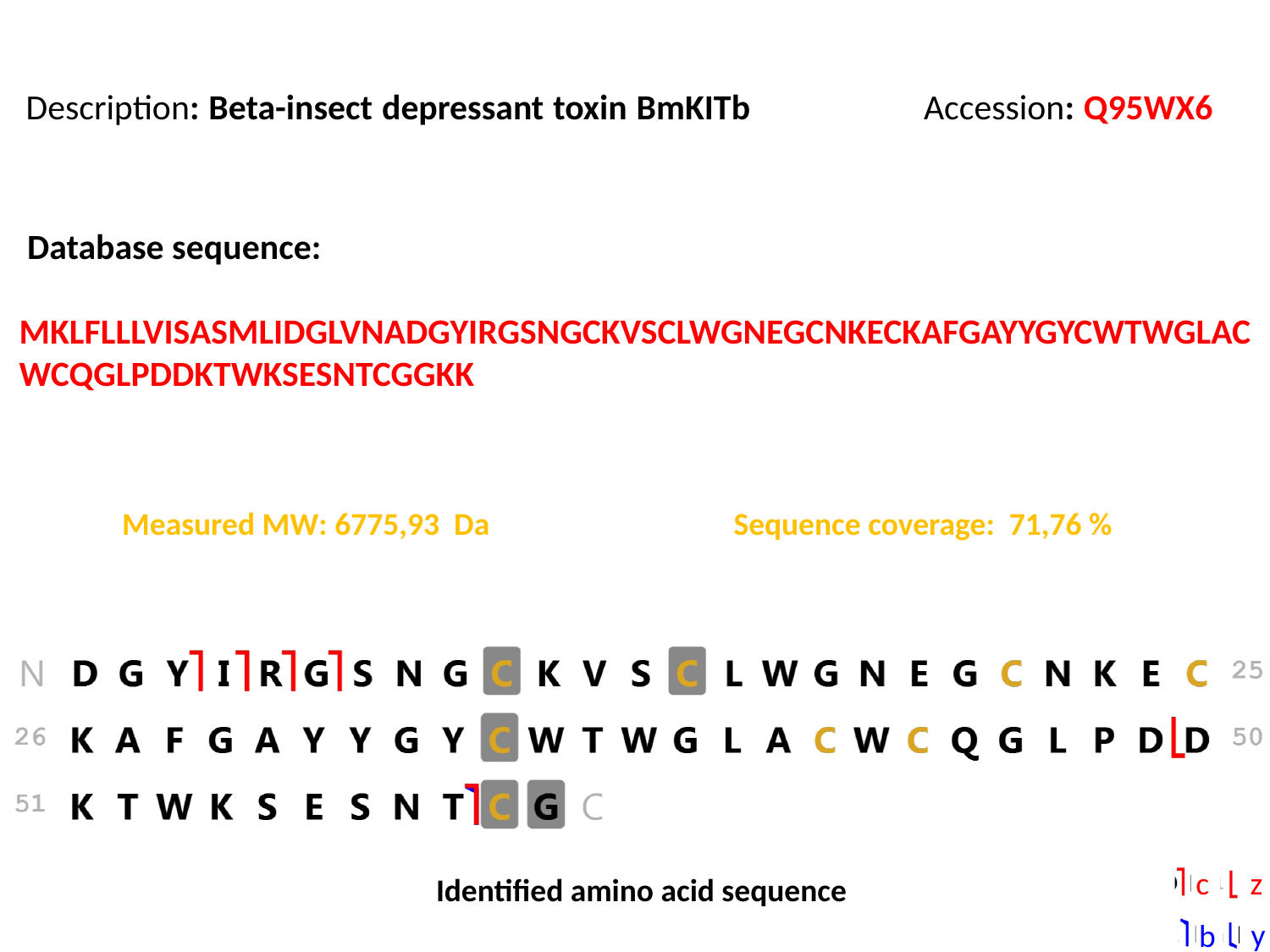

Description: Beta-insect depressant toxin BmKITb Accession: Q95WX6
 Database sequence:
MKLFLLLVISASMLIDGLVNADGYIRGSNGCKVSCLWGNEGCNKECKAFGAYYGYCWTWGLACWCQGLPDDKTWKSESNTCGGKK
Measured MW: 6775,93 Da Sequence coverage: 71,76 %
c
z
y
b
Identified amino acid sequence

## Slide 58
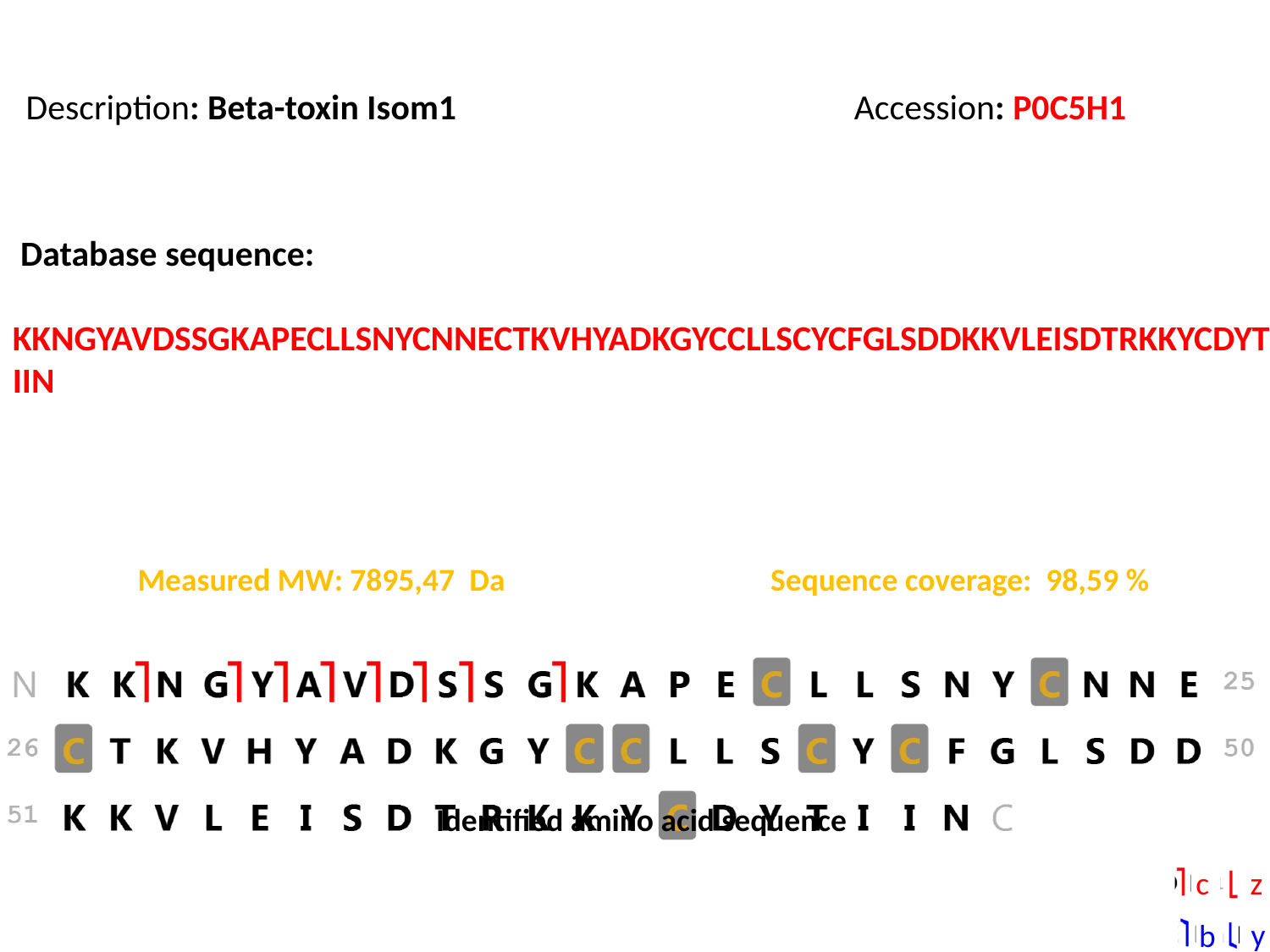

Description: Beta-toxin Isom1		 Accession: P0C5H1
 Database sequence:
KKNGYAVDSSGKAPECLLSNYCNNECTKVHYADKGYCCLLSCYCFGLSDDKKVLEISDTRKKYCDYTIIN
Measured MW: 7895,47 Da Sequence coverage: 98,59 %
Identified amino acid sequence
c
z
y
b

## Slide 59
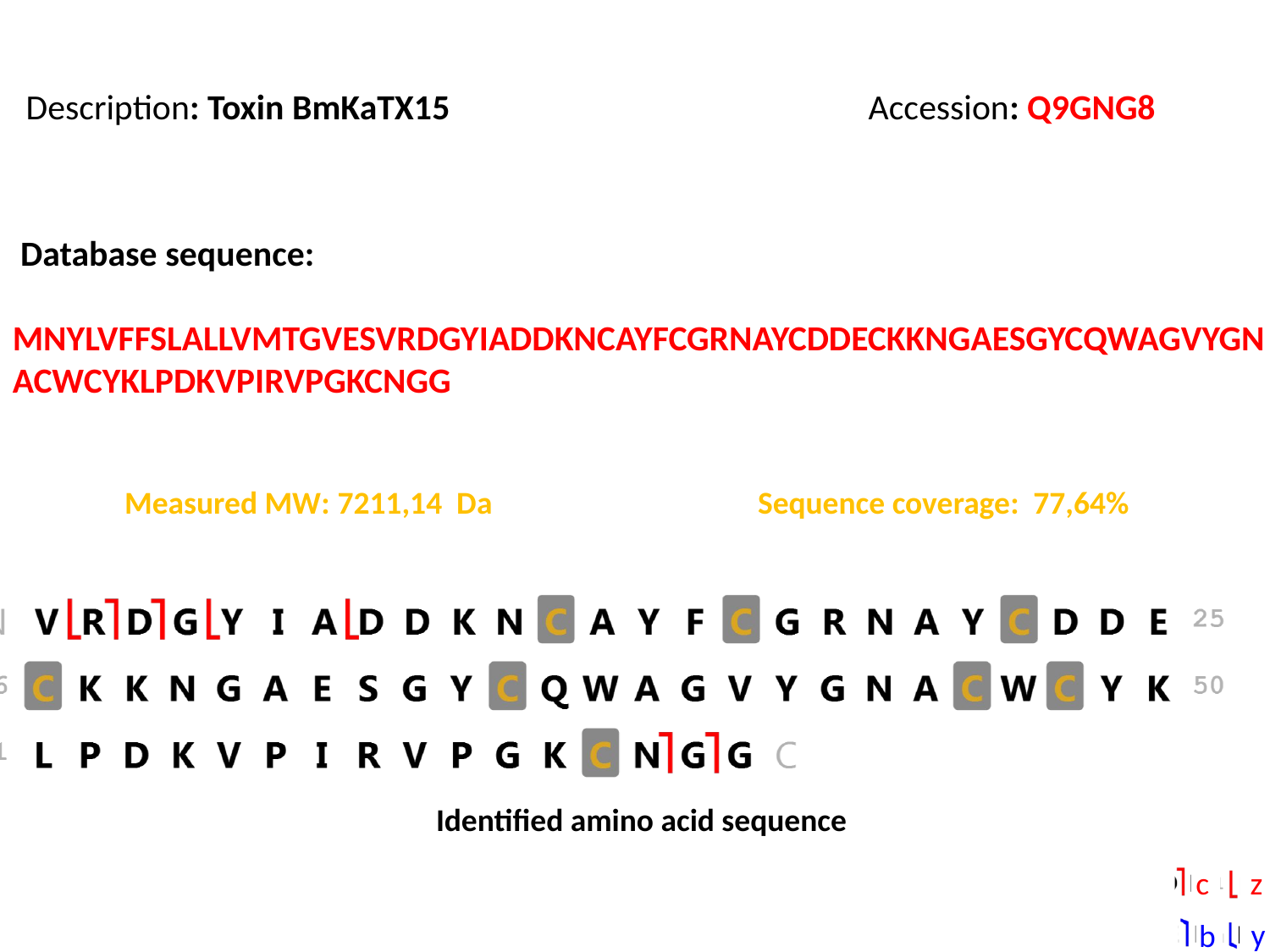

Description: Toxin BmKaTX15 Accession: Q9GNG8
 Database sequence:
MNYLVFFSLALLVMTGVESVRDGYIADDKNCAYFCGRNAYCDDECKKNGAESGYCQWAGVYGNACWCYKLPDKVPIRVPGKCNGG
Measured MW: 7211,14 Da Sequence coverage: 77,64%
Identified amino acid sequence
c
z
y
b

## Slide 60
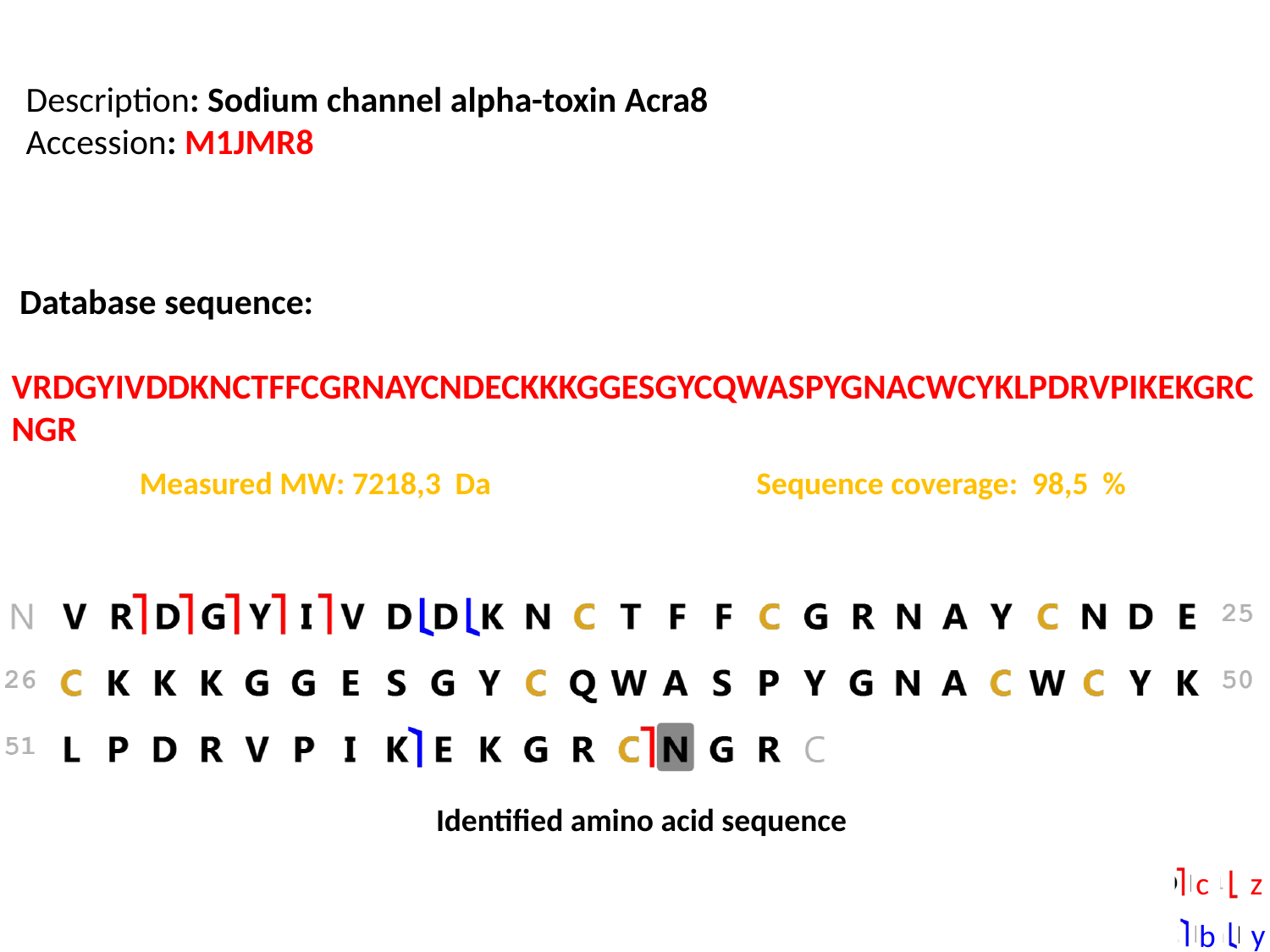

Description: Sodium channel alpha-toxin Acra8
Accession: M1JMR8
 Database sequence:
VRDGYIVDDKNCTFFCGRNAYCNDECKKKGGESGYCQWASPYGNACWCYKLPDRVPIKEKGRCNGR
Measured MW: 7218,3 Da Sequence coverage: 98,5 %
Identified amino acid sequence
c
z
y
b

## Slide 61
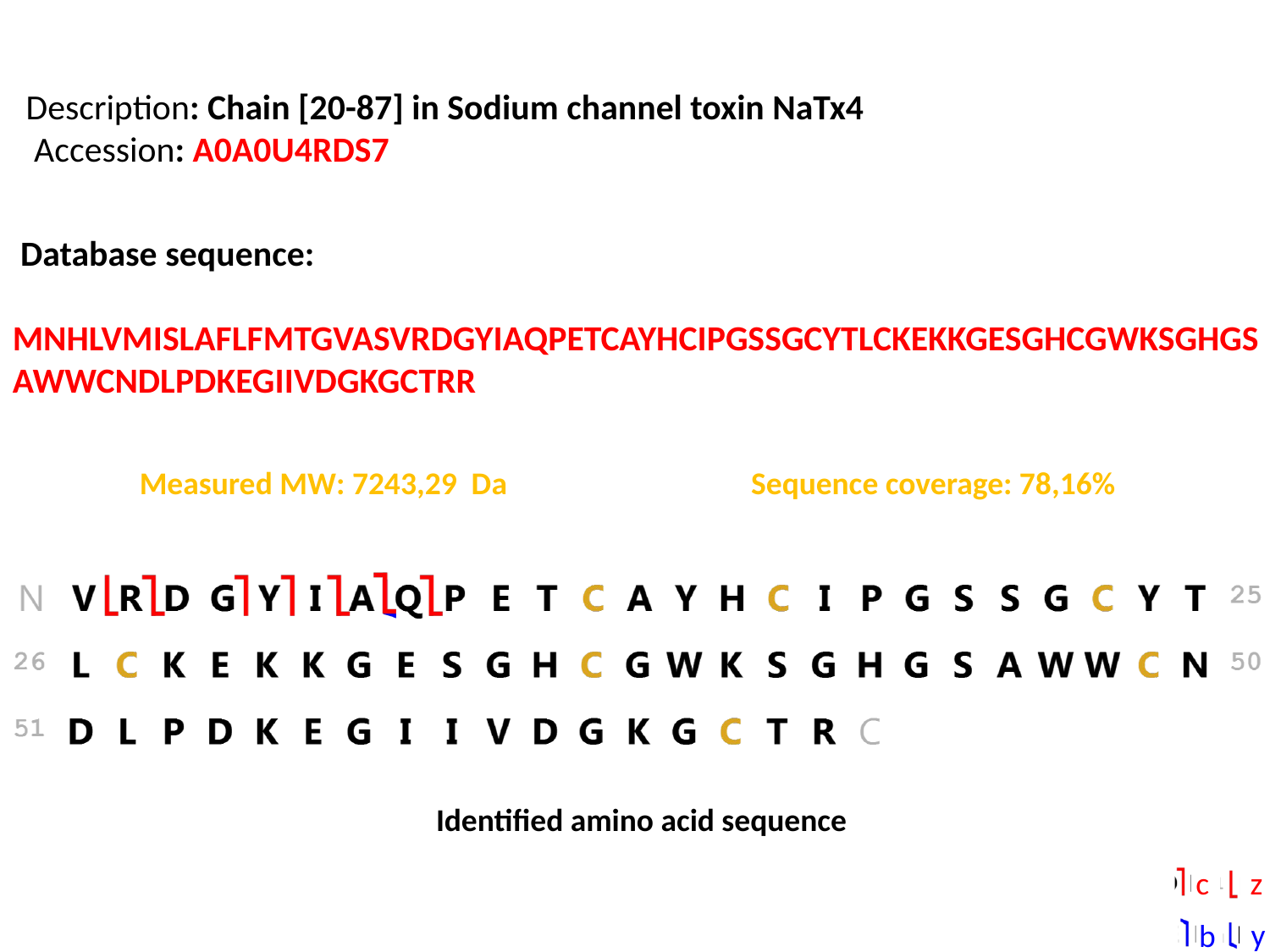

Description: Chain [20-87] in Sodium channel toxin NaTx4
 Accession: A0A0U4RDS7
 Database sequence:
MNHLVMISLAFLFMTGVASVRDGYIAQPETCAYHCIPGSSGCYTLCKEKKGESGHCGWKSGHGSAWWCNDLPDKEGIIVDGKGCTRR
Measured MW: 7243,29 Da Sequence coverage: 78,16%
Identified amino acid sequence
c
z
y
b

## Slide 62
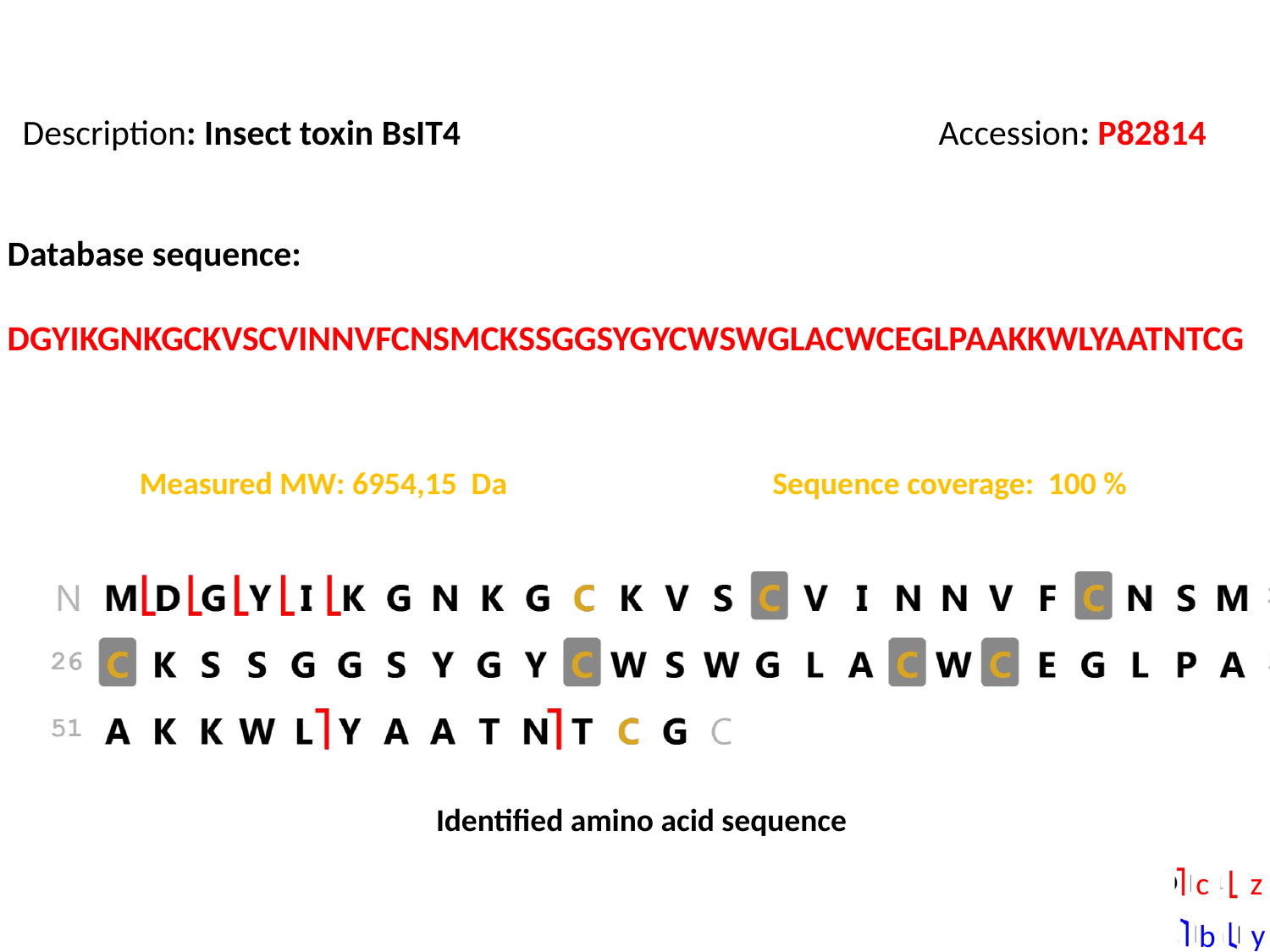

Description: Insect toxin BsIT4		 Accession: P82814
 Database sequence:
 DGYIKGNKGCKVSCVINNVFCNSMCKSSGGSYGYCWSWGLACWCEGLPAAKKWLYAATNTCG
Measured MW: 6954,15 Da Sequence coverage: 100 %
Identified amino acid sequence
c
z
y
b

## Slide 63
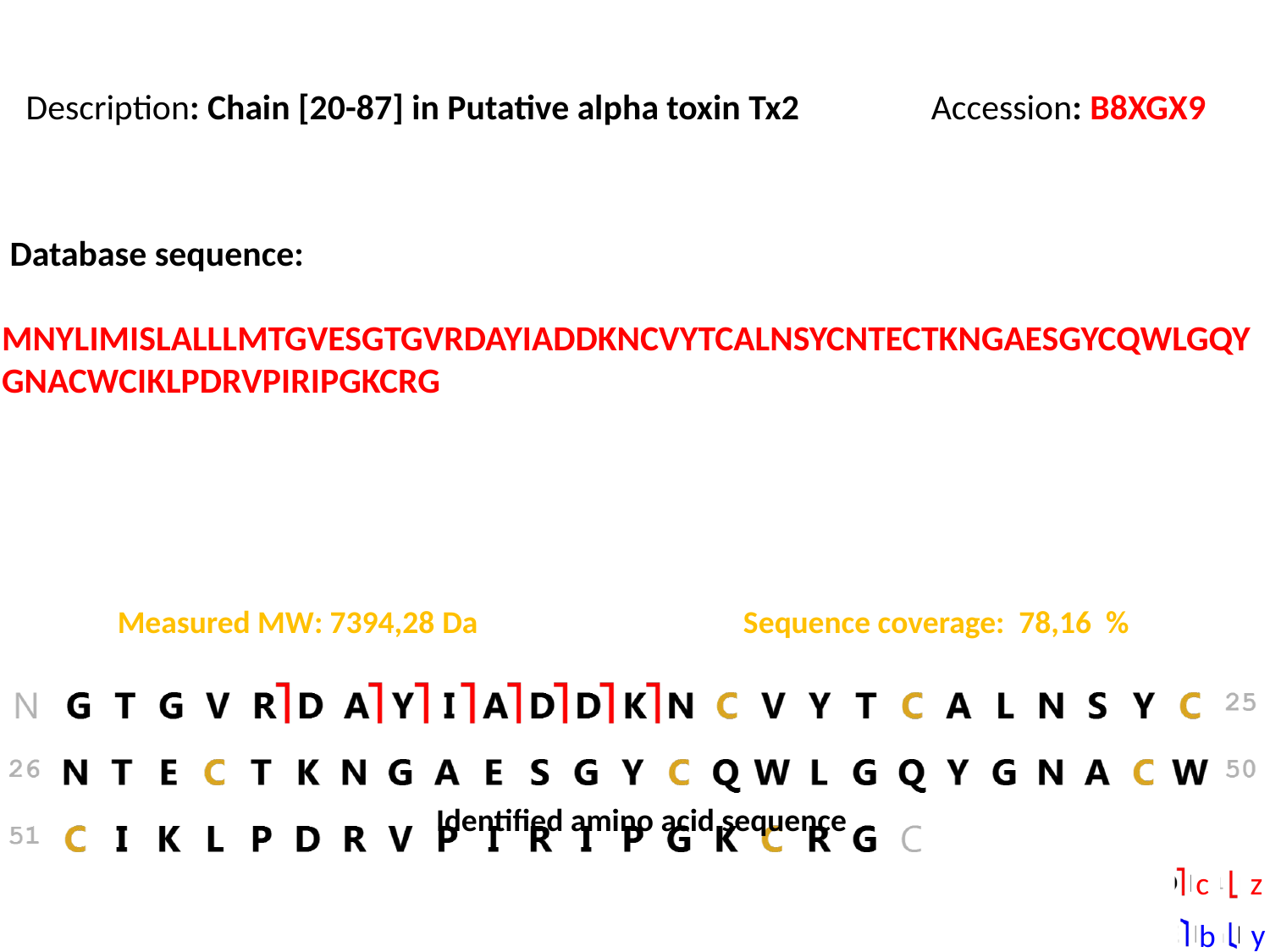

Description: Chain [20-87] in Putative alpha toxin Tx2	 Accession: B8XGX9
 Database sequence:
MNYLIMISLALLLMTGVESGTGVRDAYIADDKNCVYTCALNSYCNTECTKNGAESGYCQWLGQYGNACWCIKLPDRVPIRIPGKCRG
Measured MW: 7394,28 Da Sequence coverage: 78,16 %
Identified amino acid sequence
c
z
y
b

## Slide 64
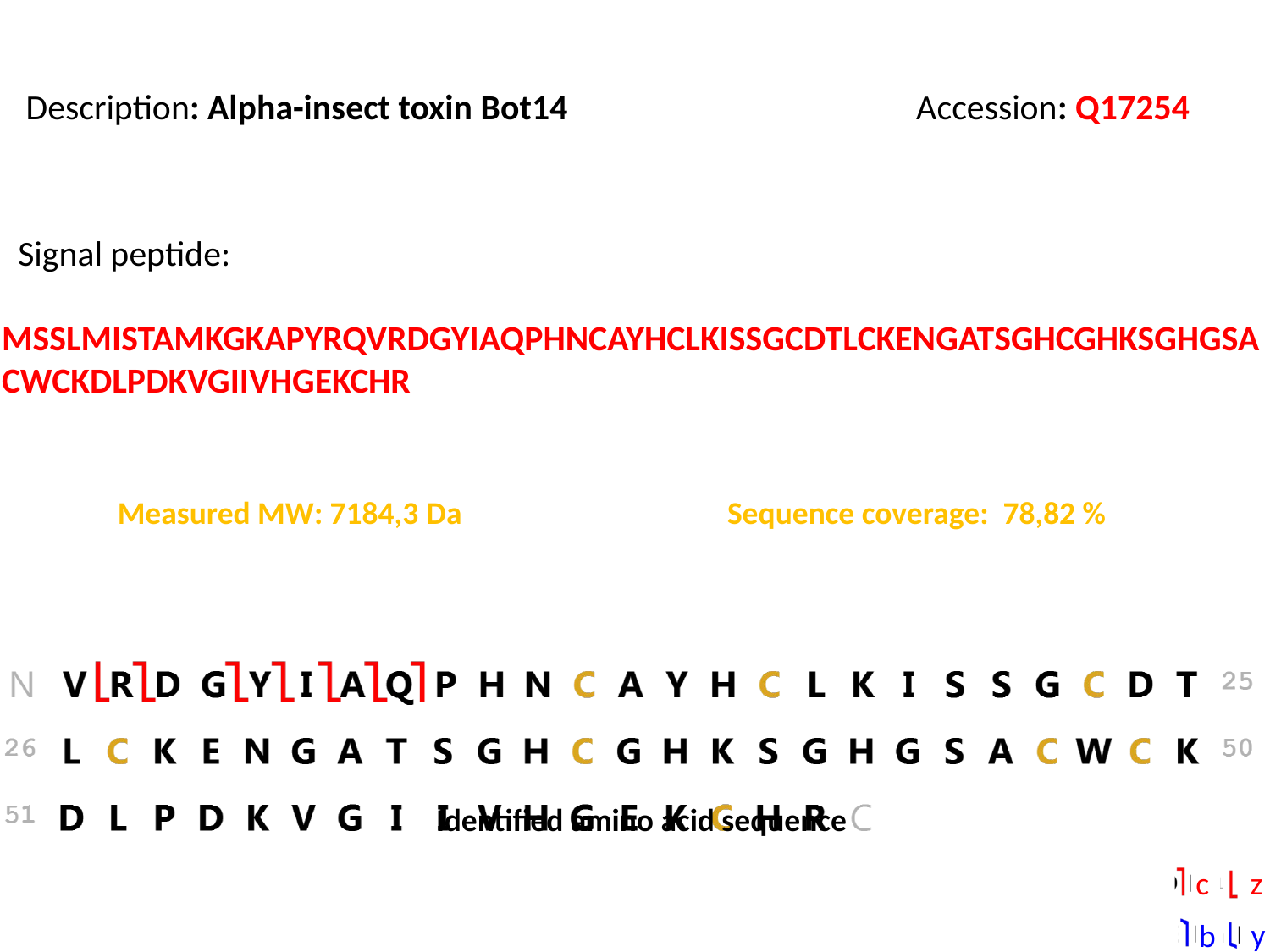

Description: Alpha-insect toxin Bot14		 Accession: Q17254
 Signal peptide:
MSSLMISTAMKGKAPYRQVRDGYIAQPHNCAYHCLKISSGCDTLCKENGATSGHCGHKSGHGSACWCKDLPDKVGIIVHGEKCHR
Measured MW: 7184,3 Da Sequence coverage: 78,82 %
Identified amino acid sequence
c
z
y
b

## Slide 65
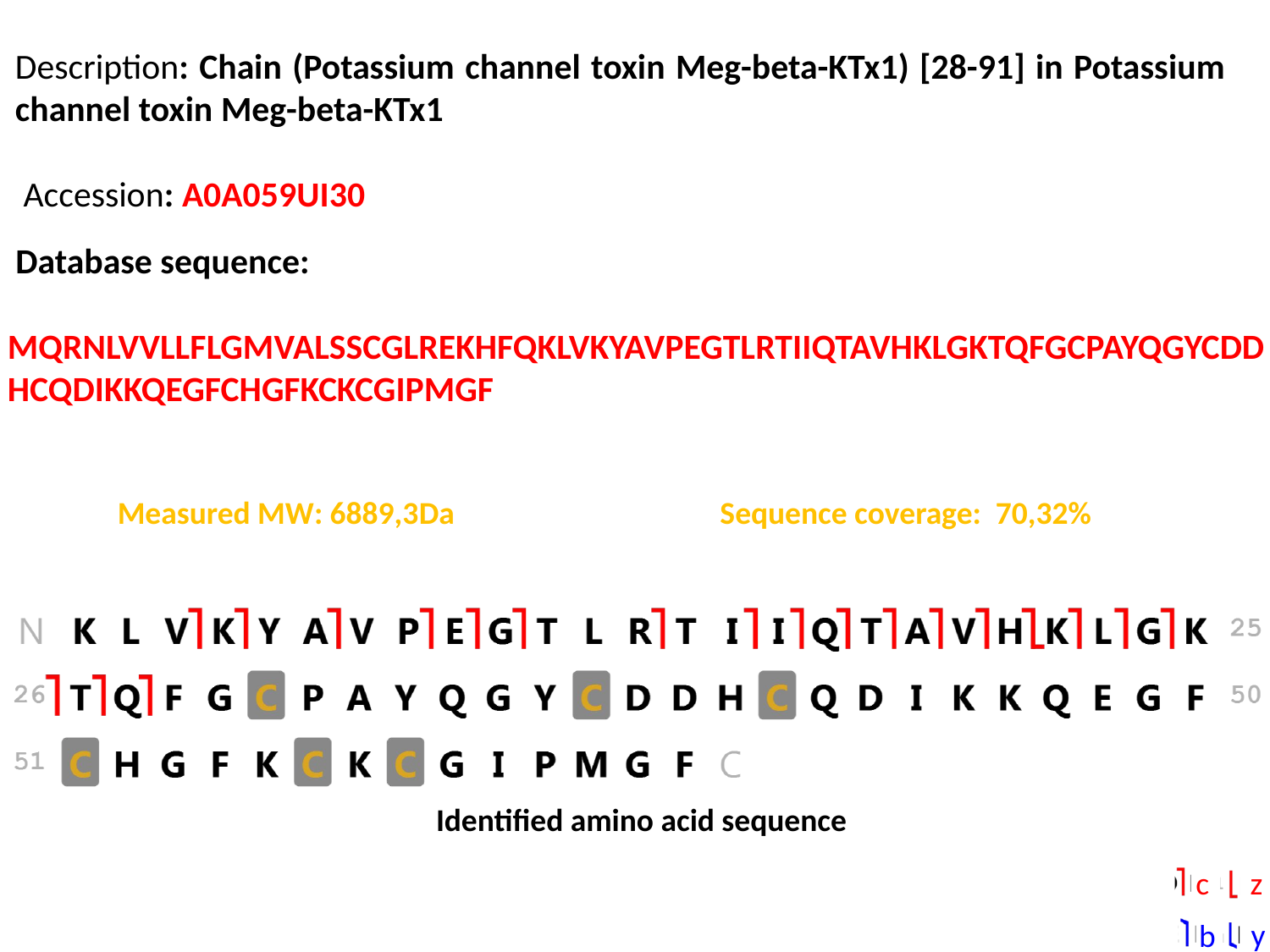

Description: Chain (Potassium channel toxin Meg-beta-KTx1) [28-91] in Potassium channel toxin Meg-beta-KTx1
 Accession: A0A059UI30
 Database sequence:
MQRNLVVLLFLGMVALSSCGLREKHFQKLVKYAVPEGTLRTIIQTAVHKLGKTQFGCPAYQGYCDDHCQDIKKQEGFCHGFKCKCGIPMGF
Measured MW: 6889,3Da Sequence coverage: 70,32%
Identified amino acid sequence
c
z
y
b

## Slide 66
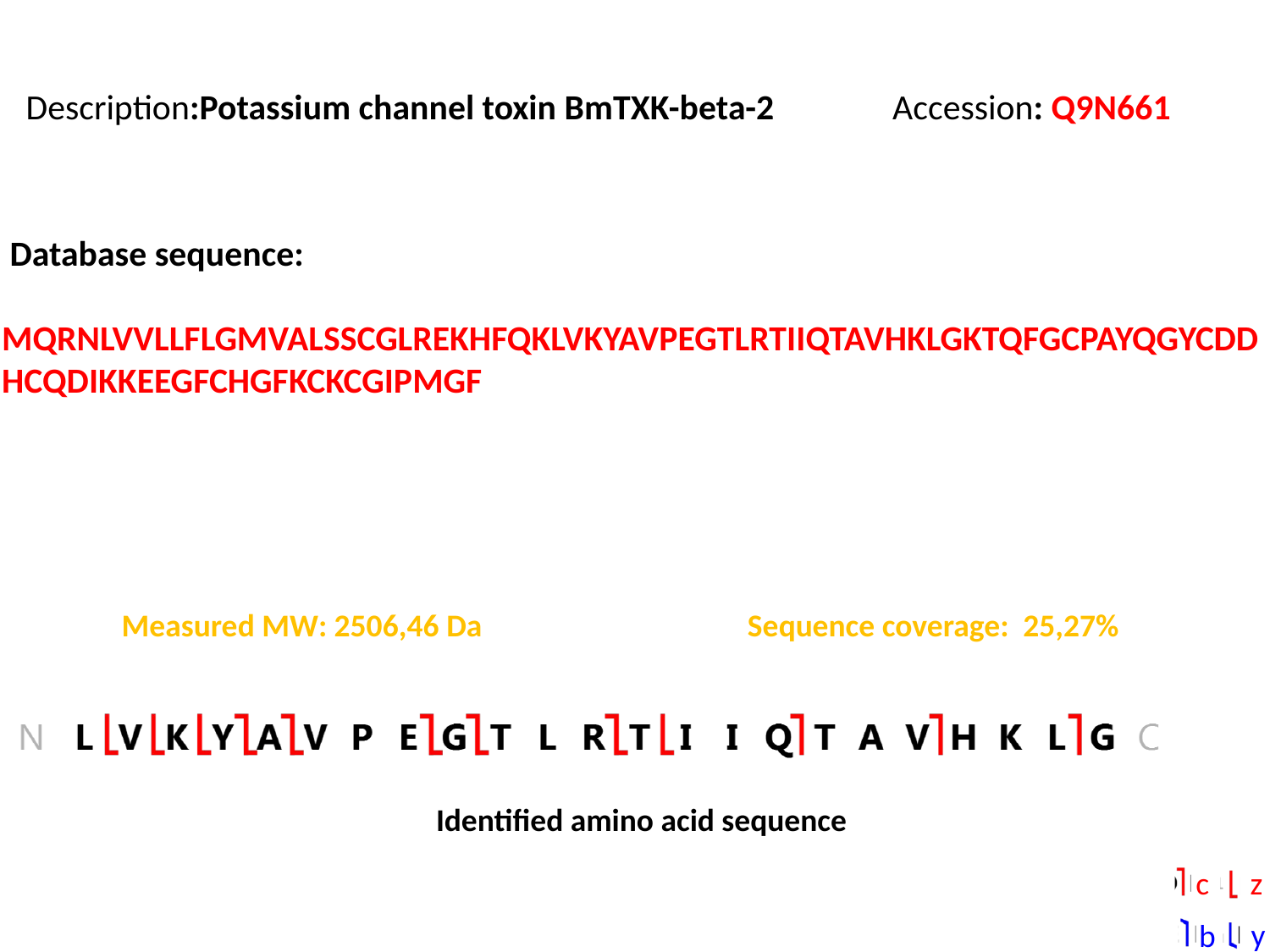

Description:Potassium channel toxin BmTXK-beta-2	 Accession: Q9N661
 Database sequence:
MQRNLVVLLFLGMVALSSCGLREKHFQKLVKYAVPEGTLRTIIQTAVHKLGKTQFGCPAYQGYCDDHCQDIKKEEGFCHGFKCKCGIPMGF
Measured MW: 2506,46 Da Sequence coverage: 25,27%
Identified amino acid sequence
c
z
y
b

## Slide 67
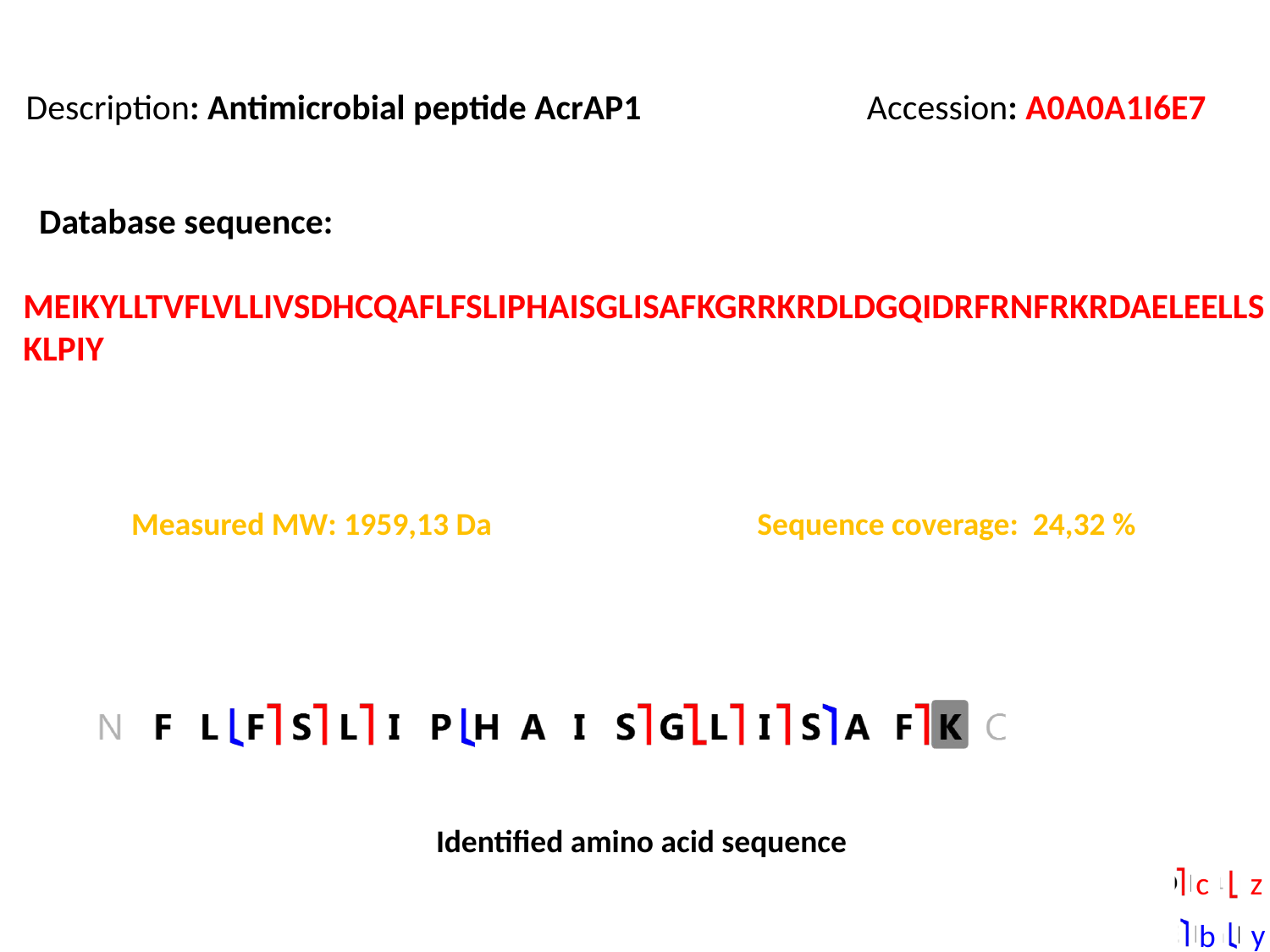

Description: Antimicrobial peptide AcrAP1 Accession: A0A0A1I6E7
 Database sequence:
MEIKYLLTVFLVLLIVSDHCQAFLFSLIPHAISGLISAFKGRRKRDLDGQIDRFRNFRKRDAELEELLSKLPIY
Measured MW: 1959,13 Da Sequence coverage: 24,32 %
Identified amino acid sequence
c
z
y
b

## Slide 68
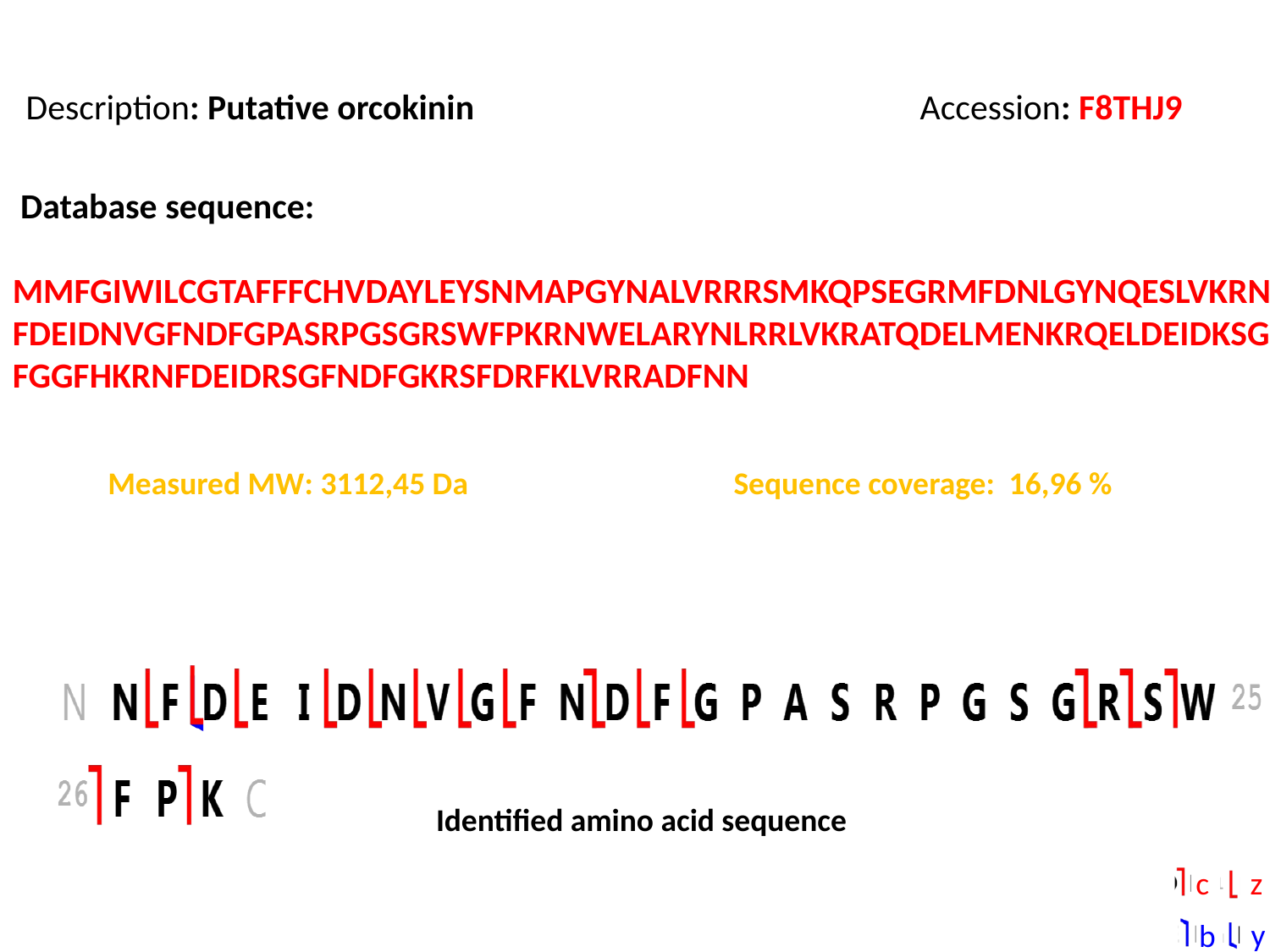

Description: Putative orcokinin	 Accession: F8THJ9
 Database sequence:
MMFGIWILCGTAFFFCHVDAYLEYSNMAPGYNALVRRRSMKQPSEGRMFDNLGYNQESLVKRNFDEIDNVGFNDFGPASRPGSGRSWFPKRNWELARYNLRRLVKRATQDELMENKRQELDEIDKSGFGGFHKRNFDEIDRSGFNDFGKRSFDRFKLVRRADFNN
Measured MW: 3112,45 Da Sequence coverage: 16,96 %
Identified amino acid sequence
c
z
y
b

## Slide 69
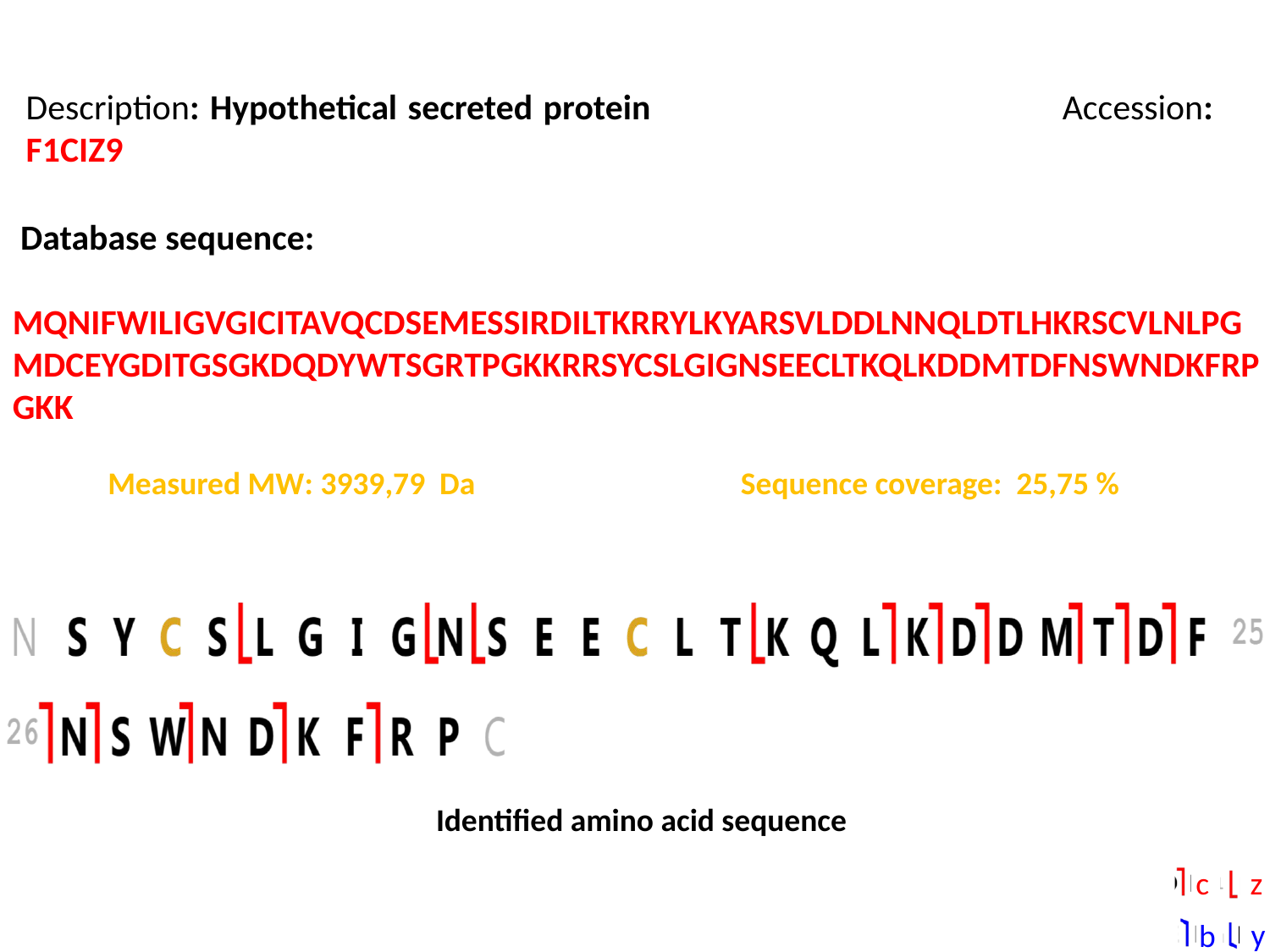

Description: Hypothetical secreted protein Accession: F1CIZ9
 Database sequence:
MQNIFWILIGVGICITAVQCDSEMESSIRDILTKRRYLKYARSVLDDLNNQLDTLHKRSCVLNLPGMDCEYGDITGSGKDQDYWTSGRTPGKKRRSYCSLGIGNSEECLTKQLKDDMTDFNSWNDKFRPGKK
Measured MW: 3939,79 Da Sequence coverage: 25,75 %
Identified amino acid sequence
c
z
y
b
